# Supplementary material for: Two FAM134B isoforms differentially regulate ER dynamics during myogenesis
Source: EMBO J. 2025 Jan 6;44(4):1039–73. doi: 10.1038/s44318-024-00356-2 (PMC11832904; doi:10.1038/s44318-024-00356-2)
Supplement: Supplementary file 1 — Appendix [file 44318_2024_356_MOESM1_ESM.pdf]

Appendix

Two FAM134B isoforms differentially regulate ER form and dynamics during myogenesis

Viviana Buonomo, Kateryna Lohachova, Alessio Reggio, Sara Cano-Franco, Michele Cillo, Lucia Santorelli, Rossella Venditti, Elena Polishchuk, Ivana Peluso, Lorene Brunello, Carmine Cirillo, Sara Petrosino, Malan Silva, Rossella De Cegli, Sabrina Di Bartolomeo, Cesare Gargioli, Paolo Swuec, Mirko Cortese, Alexandra Stolz, Ramachandra M. Bhaskara, Paolo Grumati

Table of Contents:

Appendix Figure S1…… Page 2-3
Appendix Figure S2…… Page 4-5

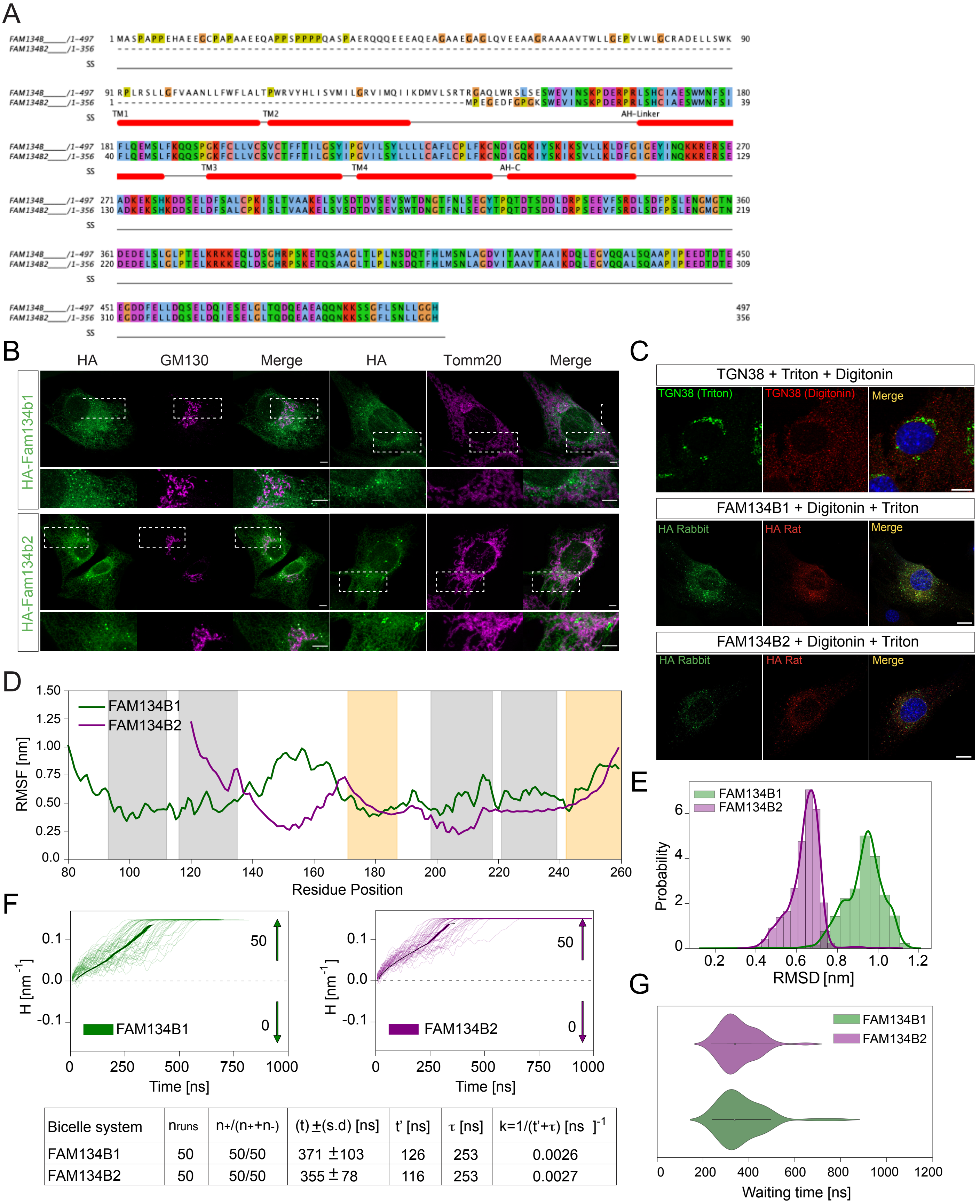

Bicelle system	n_{runs}	$n_+/(n_++n_-)$	$\langle t \rangle \pm$(s.d) [ns]	t' [ns]	τ [ns]	k=1/(t'+τ) [ns]$^{-1}$
FAM134B1	50	50/50	371 ±103	126	253	0.0026
FAM134B2	50	50/50	355 ± 78	116	253	0.0027

Appendix Figure S1

Topology and LIR domain characterization of FAM134B2

A) Sequence alignment of FAM134B1 and FAM134B2. Helical hairpins (TM1/2 and TM3/4) and amphipathic helices (AH) are highlighted in red. **B**) Confocal images of C2C12 cells overexpressing HA-FAM134B1 and HA-FAM134B2 following 24h of doxycycline induction and stained for HA and endogenous proteins GM130 for Golgi and Tomm20 for mitochondria. Scale bar: 20µm. Inset Scale bar: 5µm. **C**) Confocal images of C2C12 cells overexpressing HA-FAM134B1 or HA-FAM134B2, following 24h of doxycycline induction, stained for HA. TGN38 was endogenously stained using a specific antibody. Scale bar: 20µm. **D**) Root-mean-square fluctuations around the average mainchain residue positions (backbone beads) for FAM134B (green) and FAM134B2 (purple). The x-axis shows residue positions corresponding to the FAM134B1–RHD structure, with aligned and topologically equivalent positions of FAM134B2. TM regions (grey) and AH segments (yellow) are shaded. **E**) Pairwise RMSD distributions comparing all the conformations sampled by FAM134B1 (green) and FAM134B2 (purple) in coarse-grained MD simulations. **F**) Mean curvature time traces for bicelles containing either FAM134B1 or FAM134B2 showing bicelle-to-vesicle transitions. The table presents the summary of computations describing the kinetics of bicelle-to-vesicle transitions for FAM134B and FAM134B2. The # of replicates (n_{runs}), # of bicelles that transitioned into closed vesicles by curving along the upper/lower leaflets (n_+/n_-), mean, with average waiting times with standard deviation, $\langle t \rangle \pm s.d.$ times for the single Poisson process (t'), lag time (τ), and rate of vesiculation (k) are shown. **G**) Violin plots represent the distribution of total waiting times for bicelle-to-vesicle transitions.

Source Data are available online for this figure. Imaging was replicated in three independent experiments.

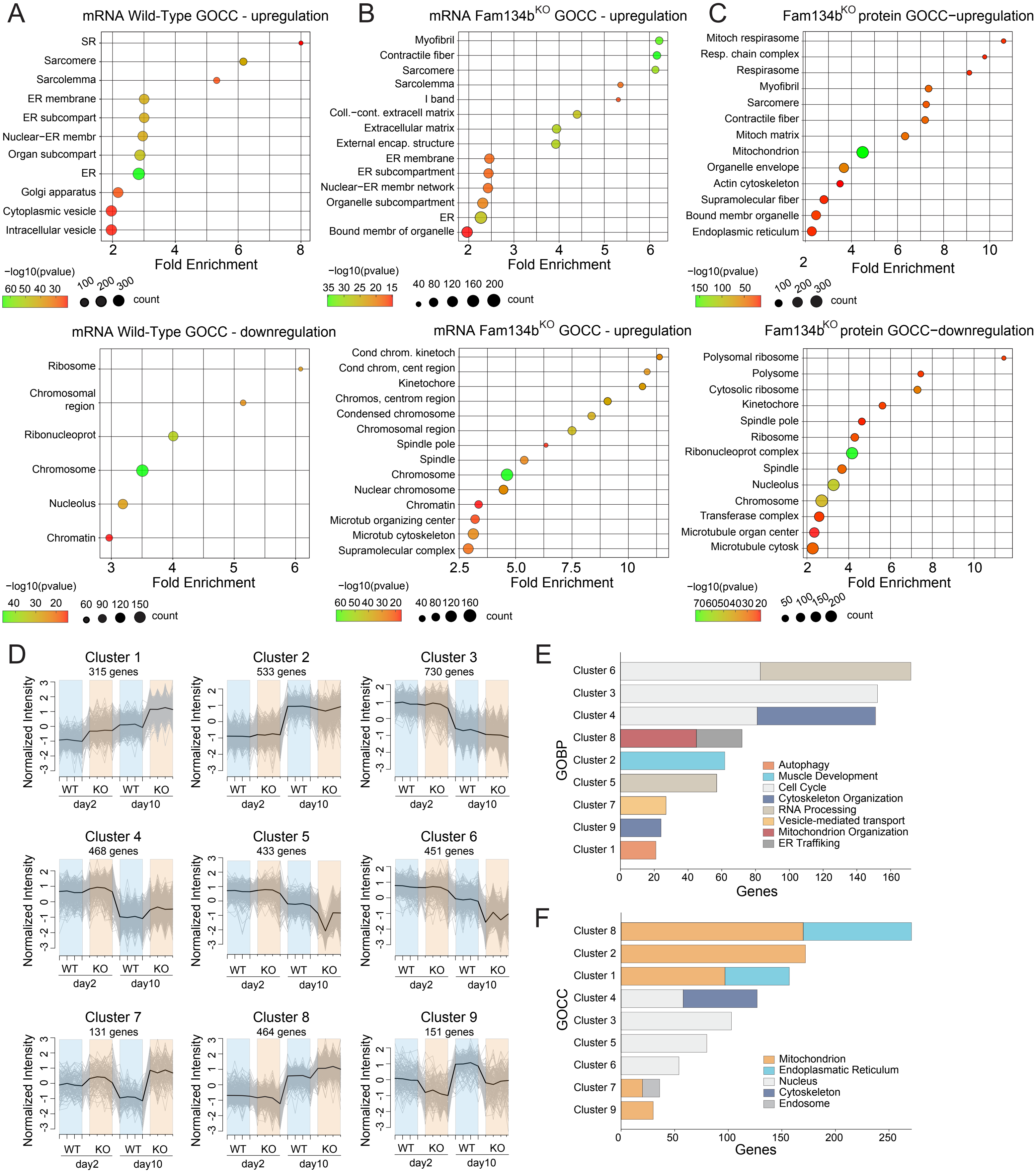

Appendix Figure S2

Lack of Fam134b influences the proteome and transcriptome of myotubes

A, **B**) GOCC terms and frequencies of up- and down-regulated genes in transcriptome of wild type (A) and Fam134bKO (B) C2C12 myoblasts and myotubes. **C**) GOCC terms and frequencies of up- and down-regulated genes identified in the proteomic analysis of Fam134bKO C2C12 myoblasts and myotubes. **D**) Profile plots of LFQ intensities for protein clusters identified by the ANOVA test from the significantly deregulated proteins in WT and Fam134bKO C2C12 myoblasts and respective myotubes. **E**,**F**) Frequency and distribution of GOBP (F) and GOCC (G) terms associated with proteins identified in the clusters reported in panel F.
