# Supplementary figures and images for: Two FAM134B isoforms differentially regulate ER dynamics during myogenesis (part 1 of 2)
Source: EMBO J. 2025 Jan 6;44(4):1039–73. doi: 10.1038/s44318-024-00356-2 (PMC11832904; doi:10.1038/s44318-024-00356-2)

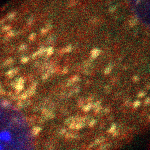

Supplement: Supplementary file 15 — Source data Fig. 1 [file 44318_2024_356_MOESM15_ESM.zip › Figure 1/Fig 1I Myotubes Inset.tif]

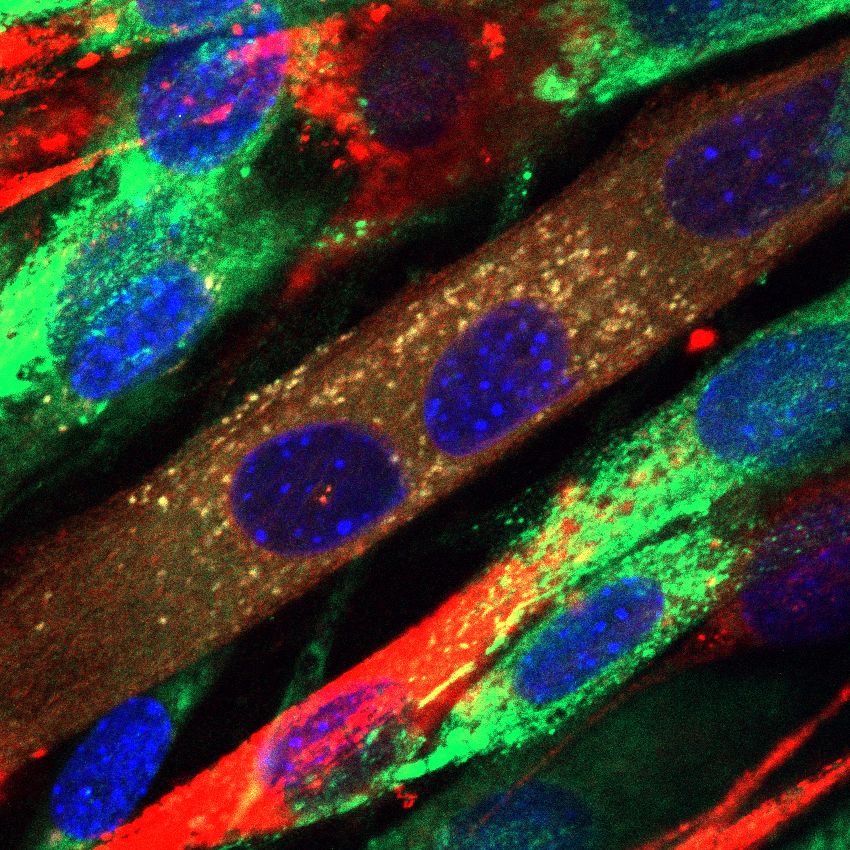

Supplement: Supplementary file 15 — Source data Fig. 1 [file 44318_2024_356_MOESM15_ESM.zip › Figure 1/Fig 1I Myotubes.tif]

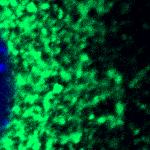

Supplement: Supplementary file 15 — Source data Fig. 1 [file 44318_2024_356_MOESM15_ESM.zip › Figure 1/Fig 1I Inset Myoblasts (GFP).tif]

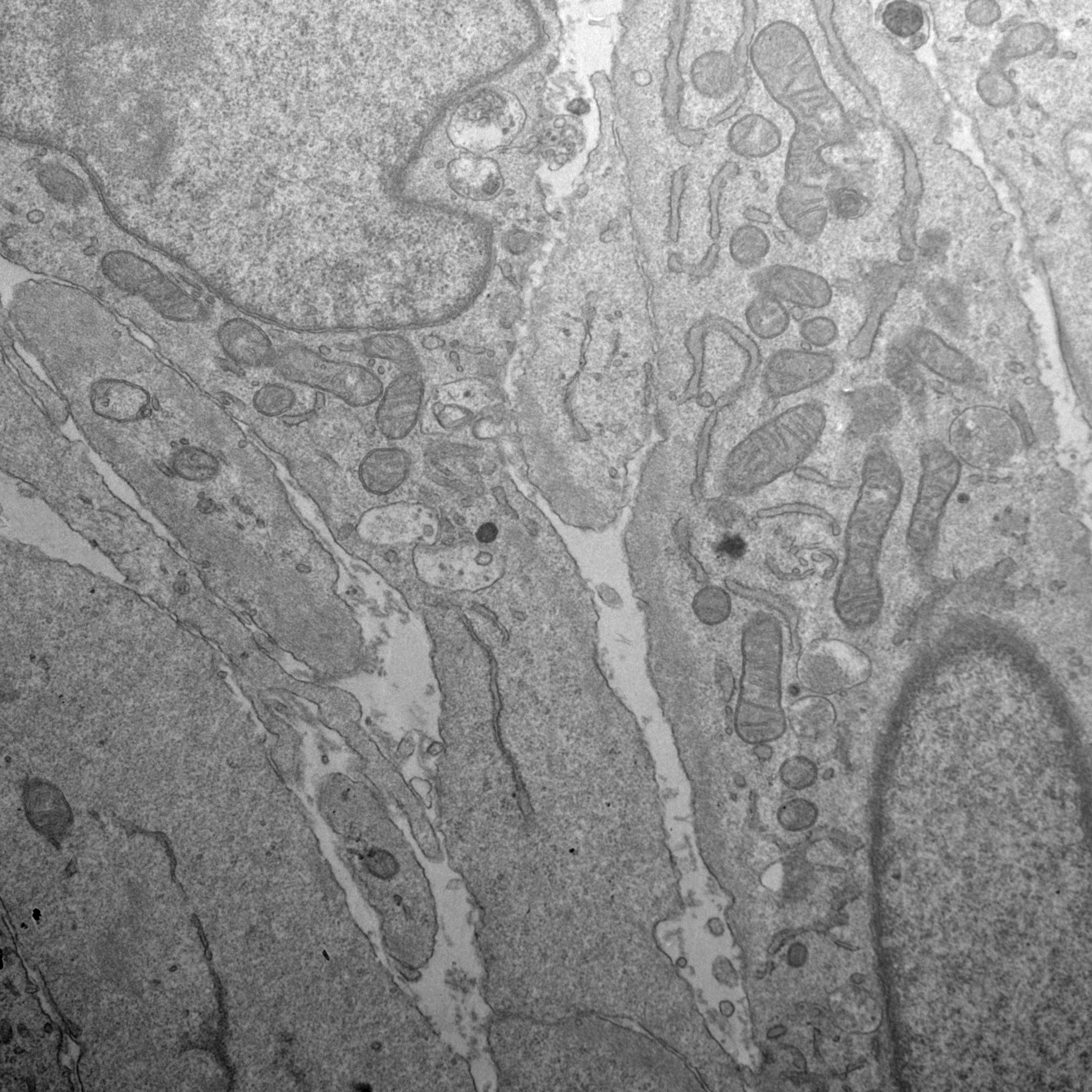

Supplement: Supplementary file 15 — Source data Fig. 1 [file 44318_2024_356_MOESM15_ESM.zip › Figure 1/Fig 1J Myotubes 10d.tif]

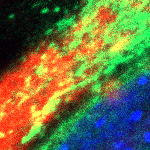

Supplement: Supplementary file 15 — Source data Fig. 1 [file 44318_2024_356_MOESM15_ESM.zip › Figure 1/Fig 1I Myotubes Inset2.tif]

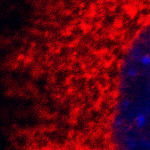

Supplement: Supplementary file 15 — Source data Fig. 1 [file 44318_2024_356_MOESM15_ESM.zip › Figure 1/Fig 1I Inset Myoblasts (RFP).tif]

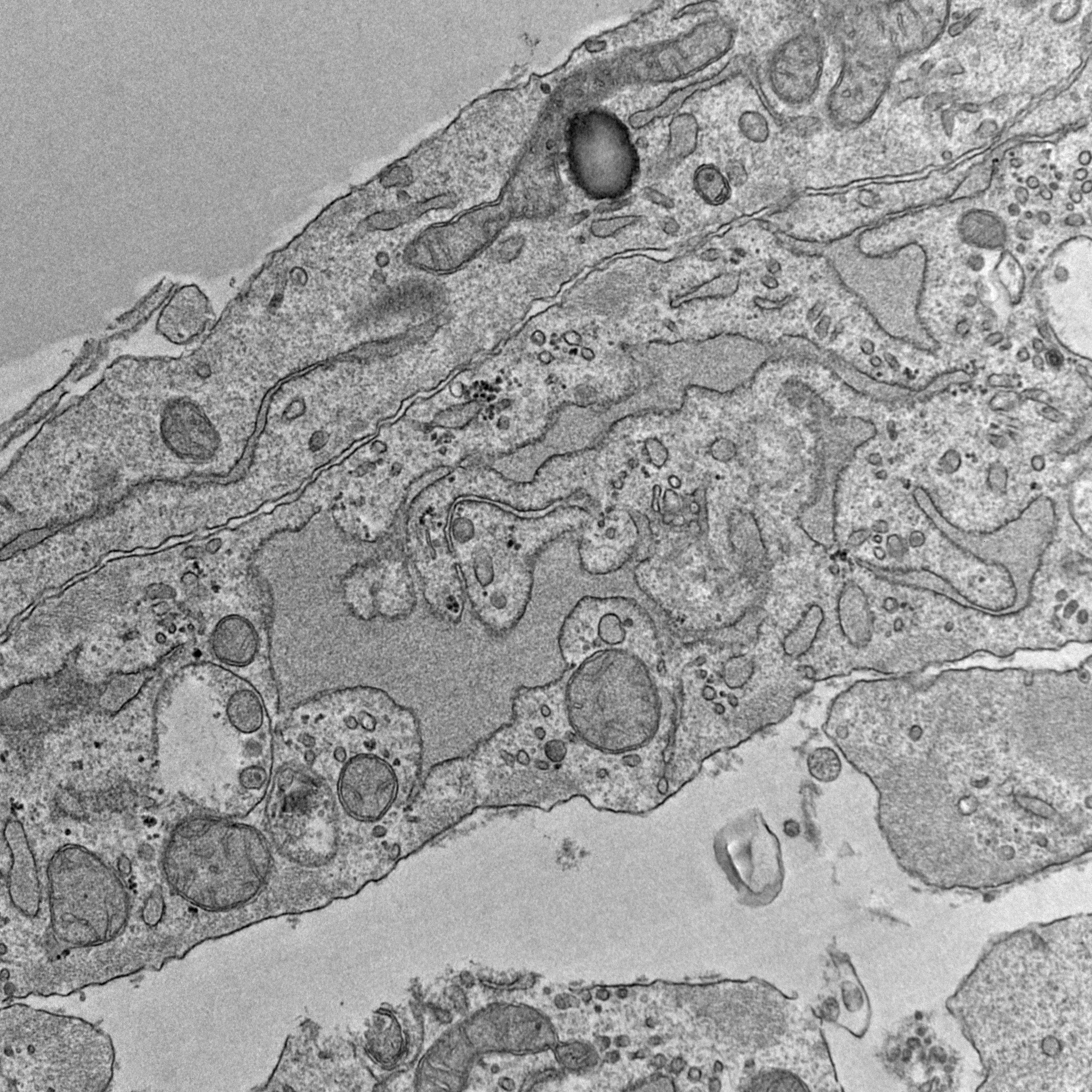

Supplement: Supplementary file 15 — Source data Fig. 1 [file 44318_2024_356_MOESM15_ESM.zip › Figure 1/Fig 1J Myotubes 7d.tif]

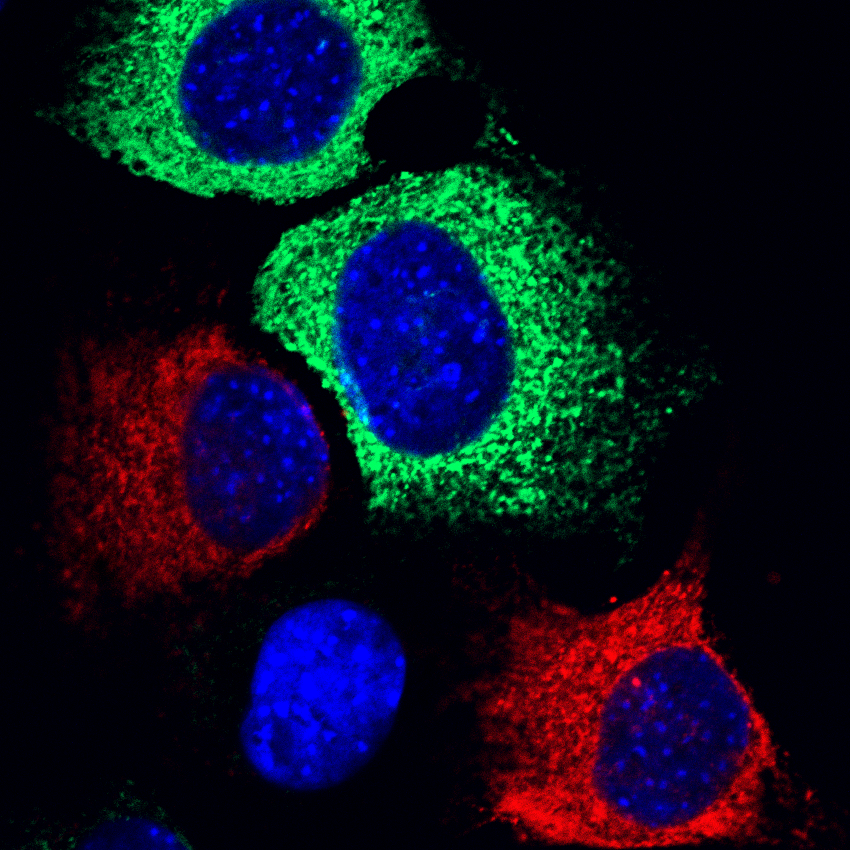

Supplement: Supplementary file 15 — Source data Fig. 1 [file 44318_2024_356_MOESM15_ESM.zip › Figure 1/Fig 1I Myoblasts.tif]

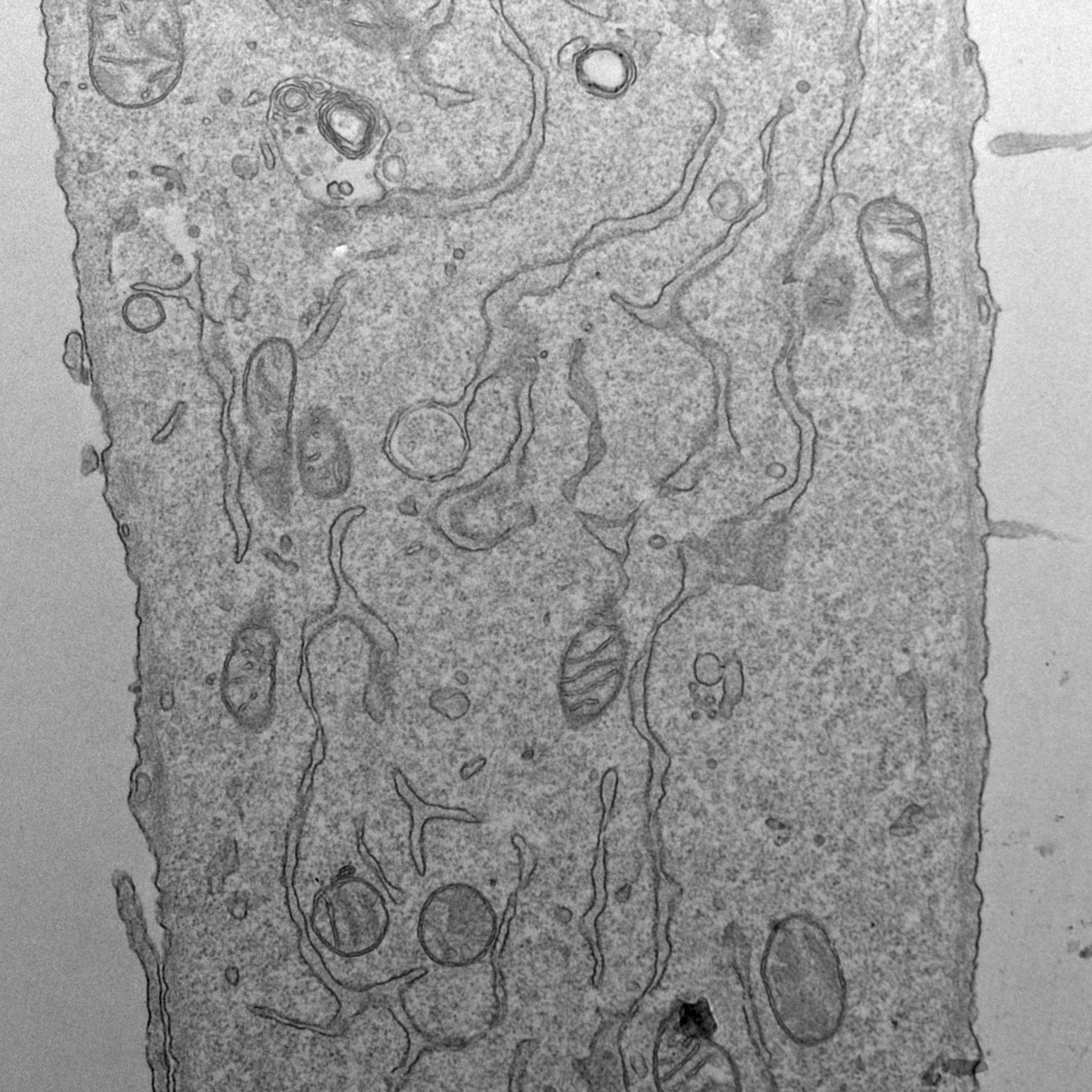

Supplement: Supplementary file 15 — Source data Fig. 1 [file 44318_2024_356_MOESM15_ESM.zip › Figure 1/Fig 1J Myoblasts.tif]

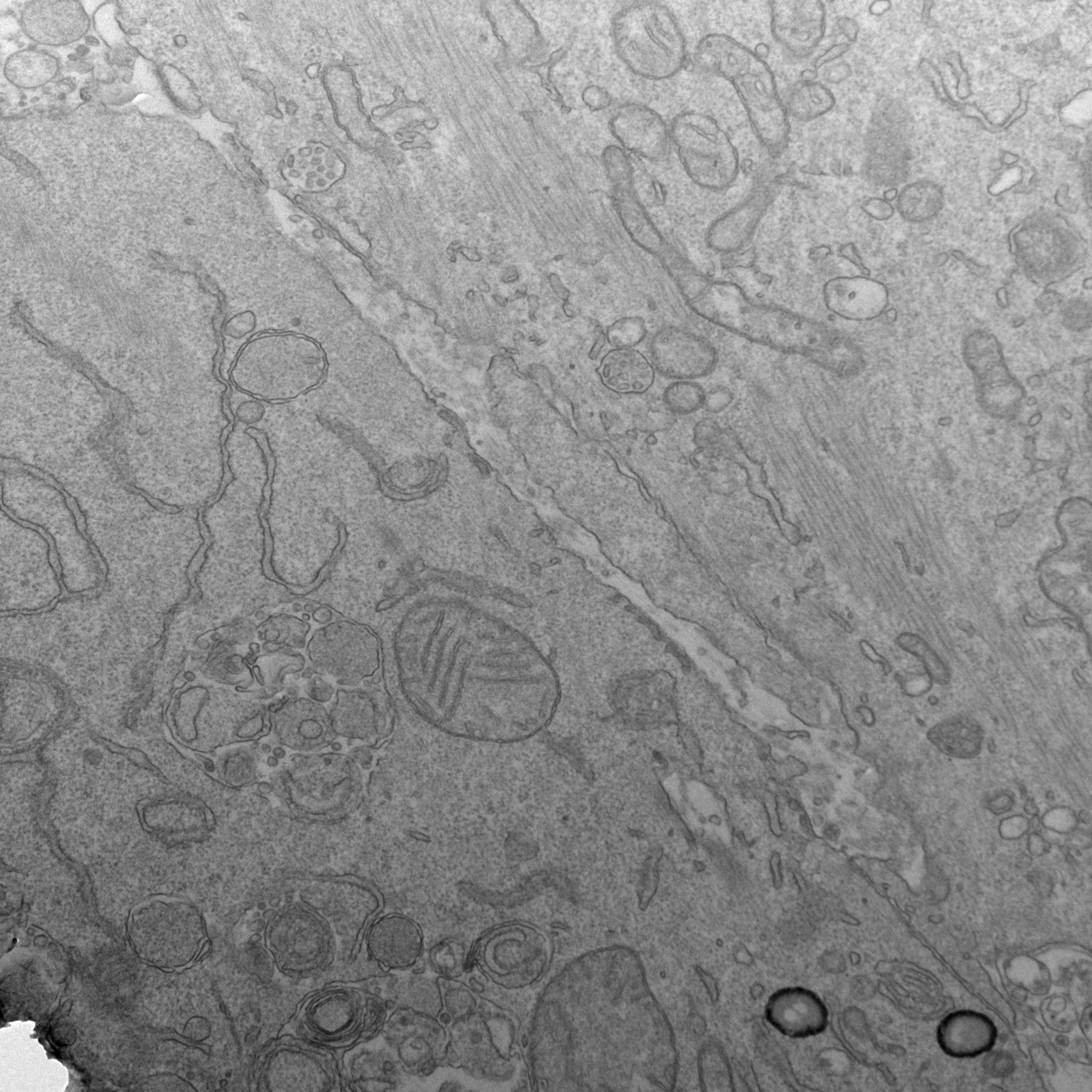

Supplement: Supplementary file 16 — Source data Fig. 2 [file 44318_2024_356_MOESM16_ESM.zip › Figure 2/Fig 2A Myotubes 10d.tif]

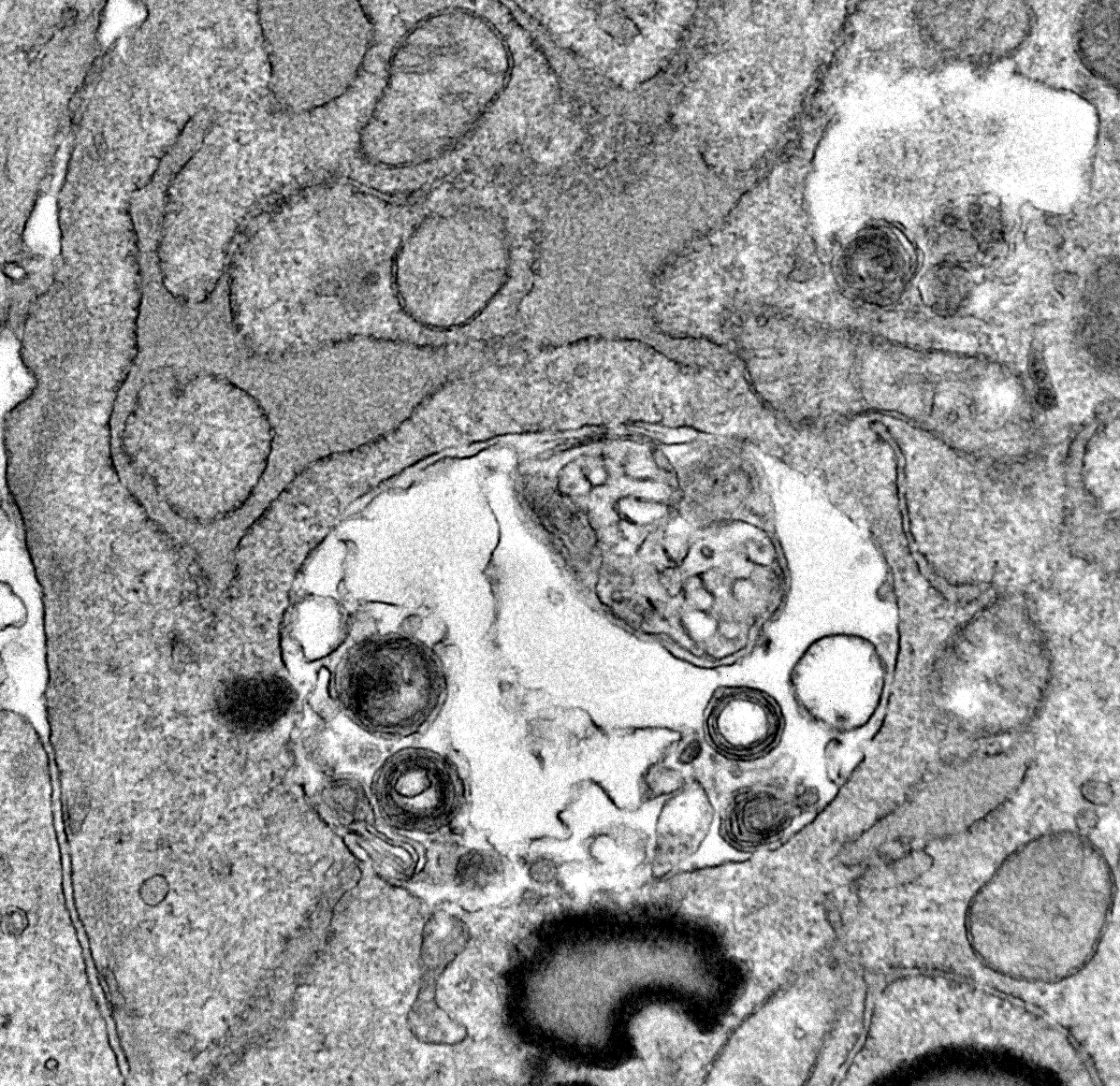

Supplement: Supplementary file 16 — Source data Fig. 2 [file 44318_2024_356_MOESM16_ESM.zip › Figure 2/Fig 2A Myotubes 7d crop.tif]

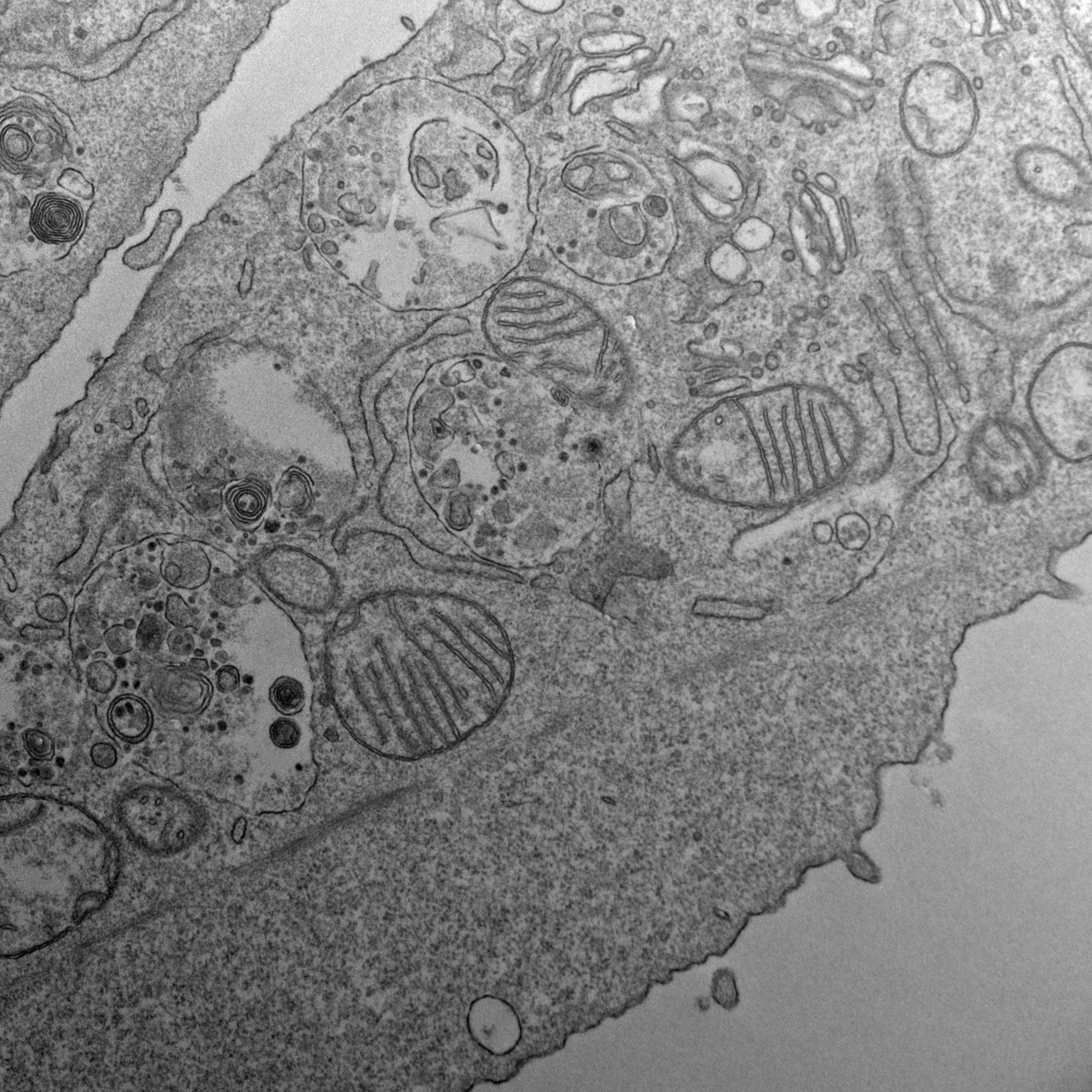

Supplement: Supplementary file 16 — Source data Fig. 2 [file 44318_2024_356_MOESM16_ESM.zip › Figure 2/Fig 2A Myoblasts.tif]

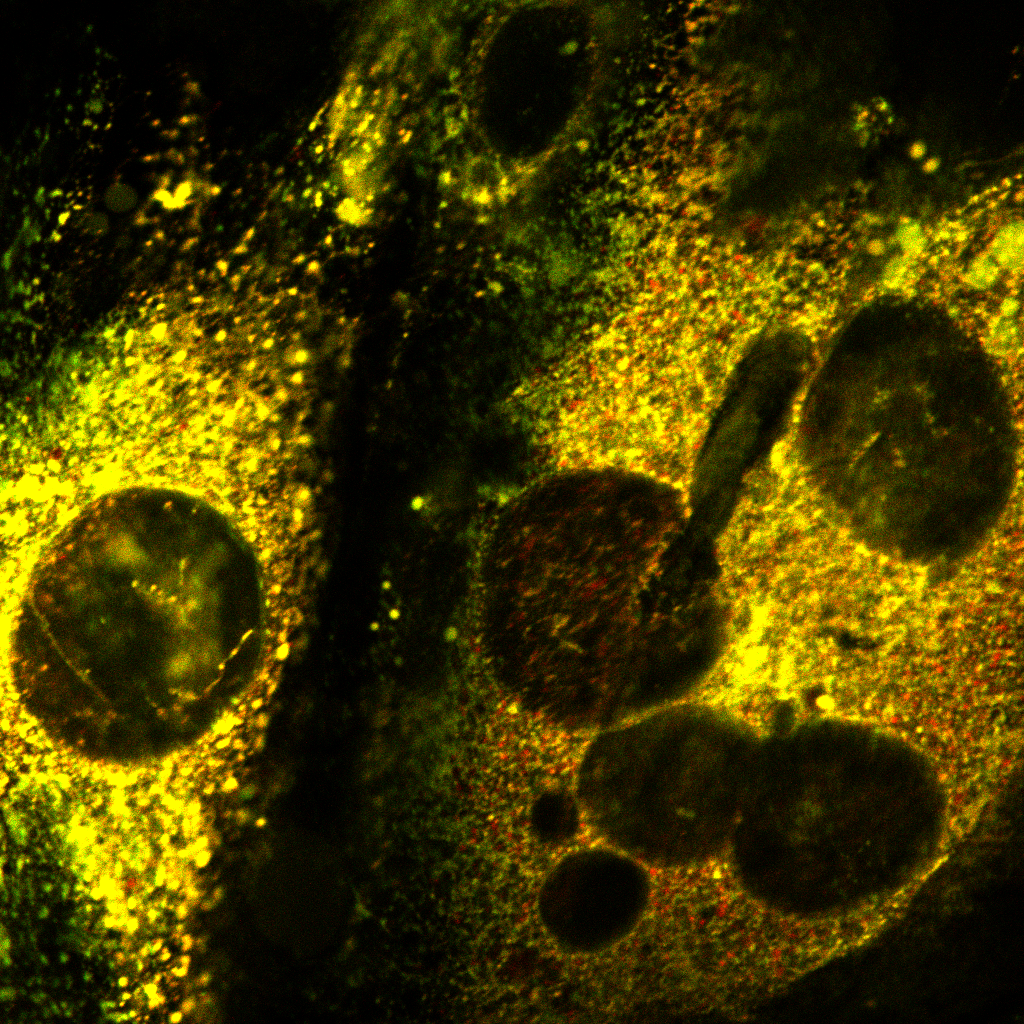

Supplement: Supplementary file 16 — Source data Fig. 2 [file 44318_2024_356_MOESM16_ESM.zip › Figure 2/Fig 2D (day5).tif]

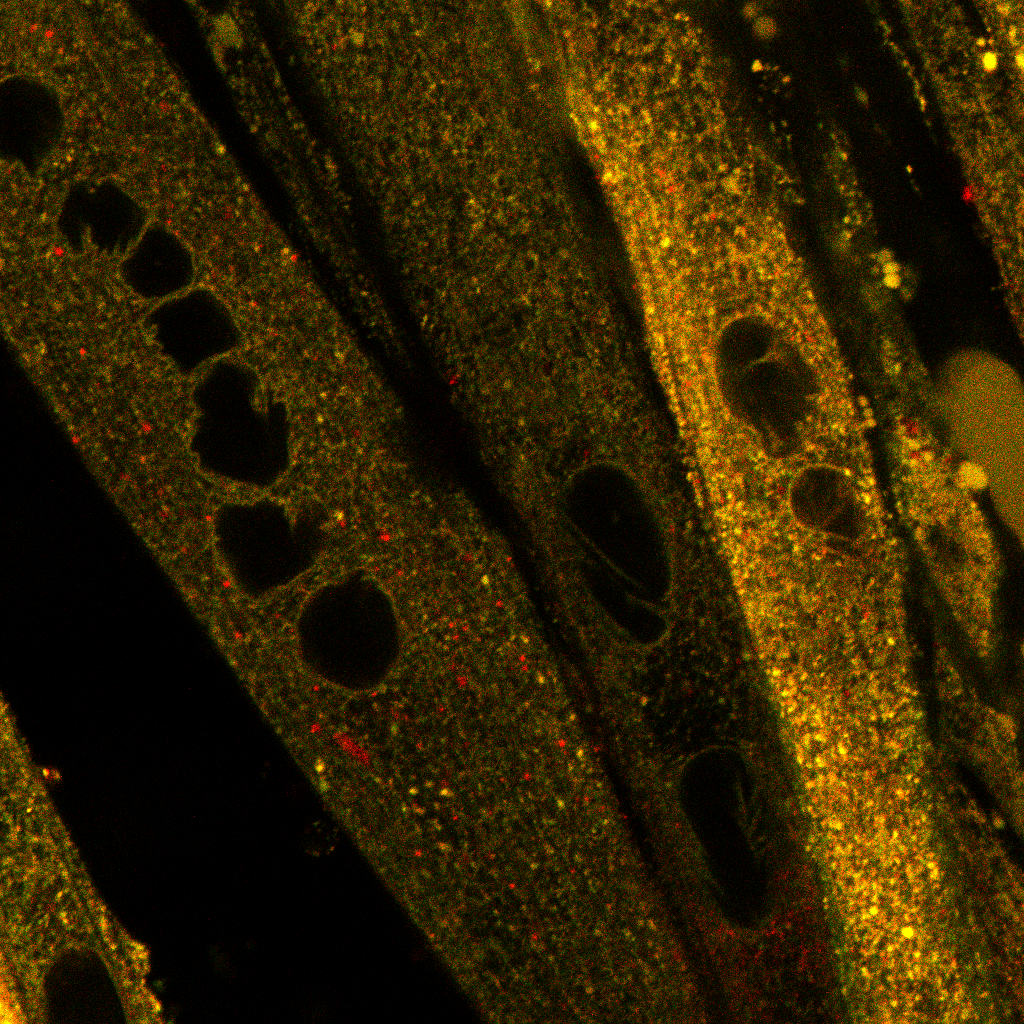

Supplement: Supplementary file 16 — Source data Fig. 2 [file 44318_2024_356_MOESM16_ESM.zip › Figure 2/Fig 2D (day10).tif]

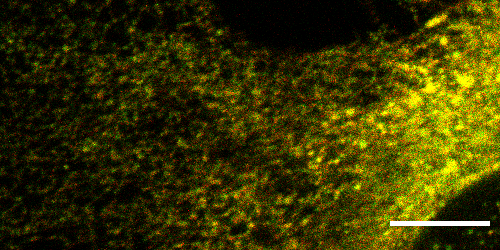

Supplement: Supplementary file 16 — Source data Fig. 2 [file 44318_2024_356_MOESM16_ESM.zip › Figure 2/Fig 2D (day1) inset.tif]

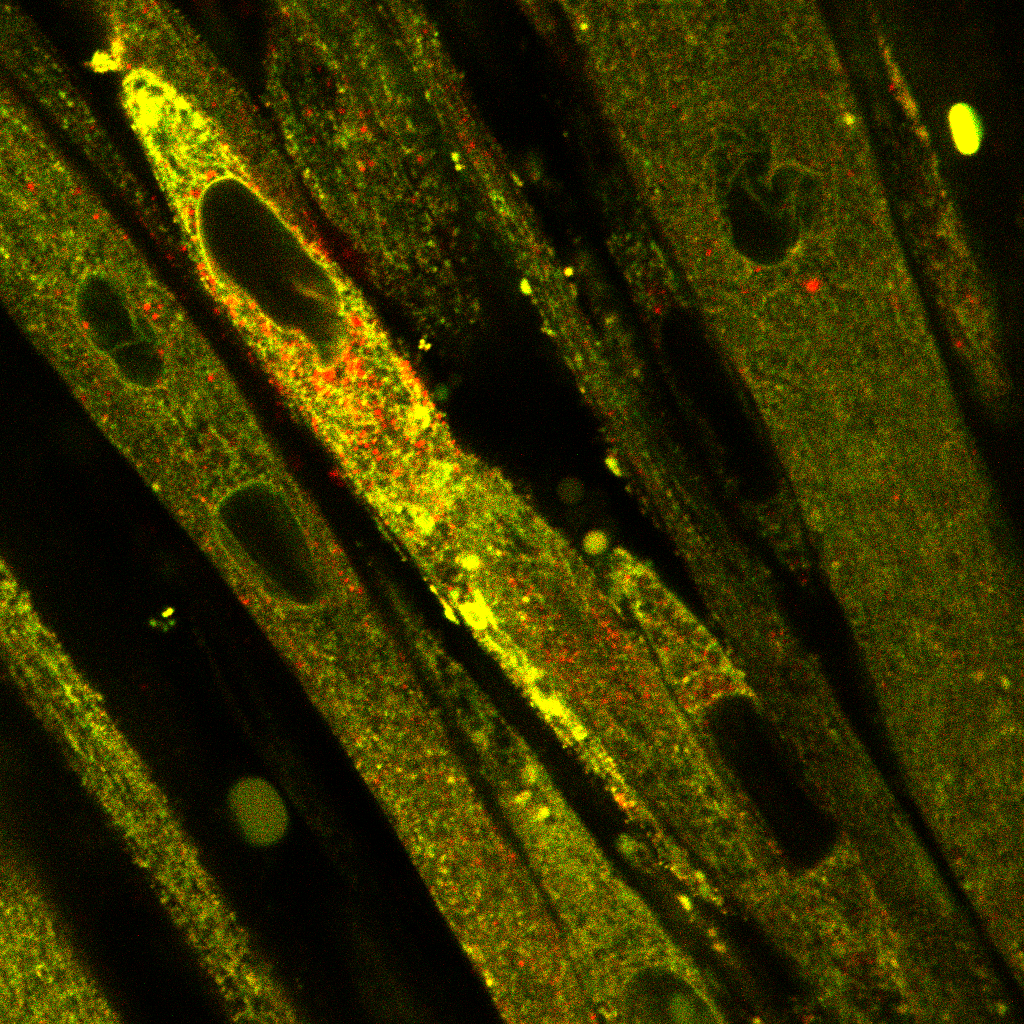

Supplement: Supplementary file 16 — Source data Fig. 2 [file 44318_2024_356_MOESM16_ESM.zip › Figure 2/Fig 2D (day7).tif]

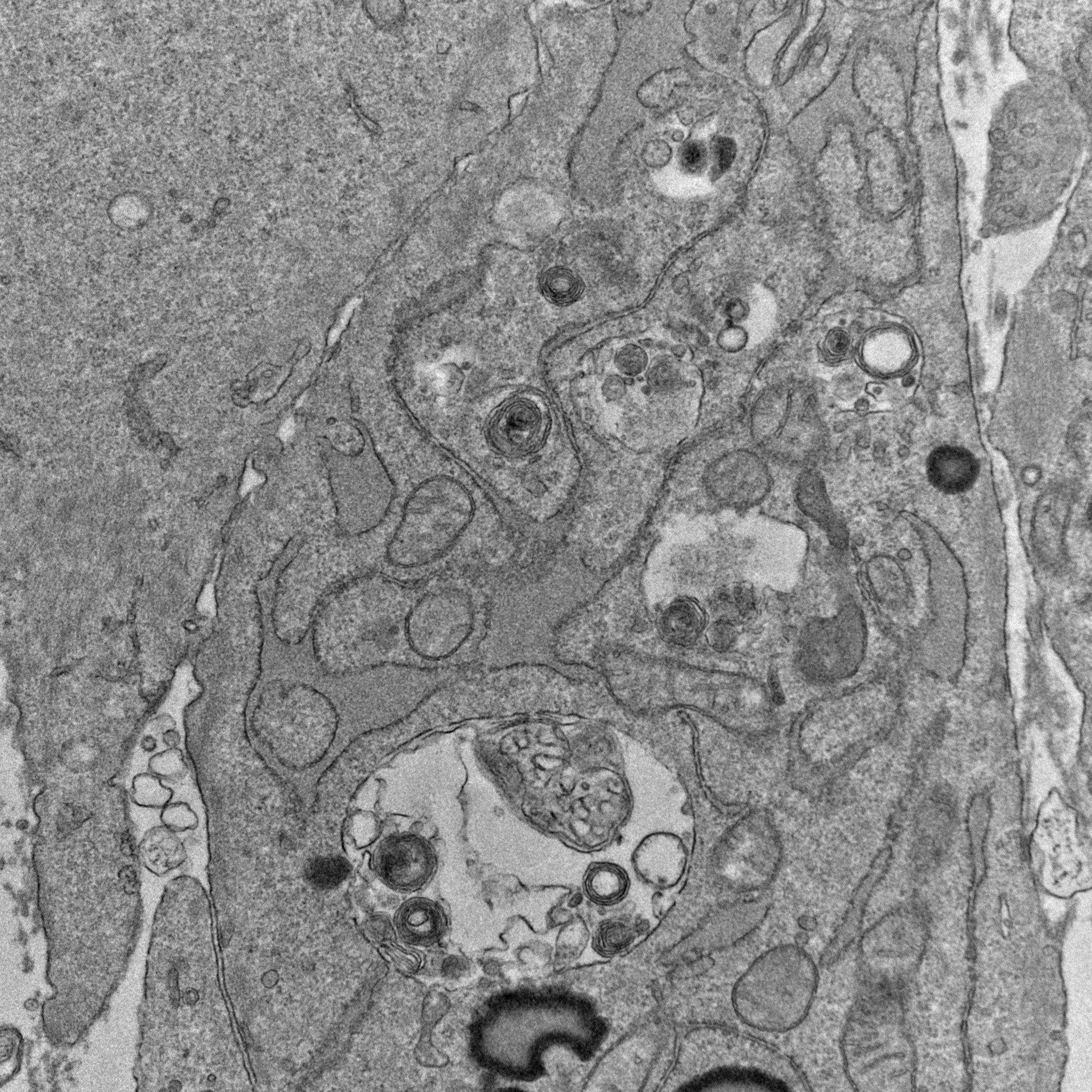

Supplement: Supplementary file 16 — Source data Fig. 2 [file 44318_2024_356_MOESM16_ESM.zip › Figure 2/Fig 2A Myotubes 7d.tif]

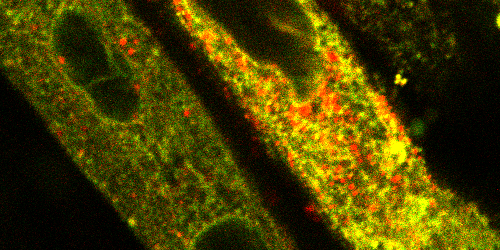

Supplement: Supplementary file 16 — Source data Fig. 2 [file 44318_2024_356_MOESM16_ESM.zip › Figure 2/Fig 2D (day7) inset.tif]

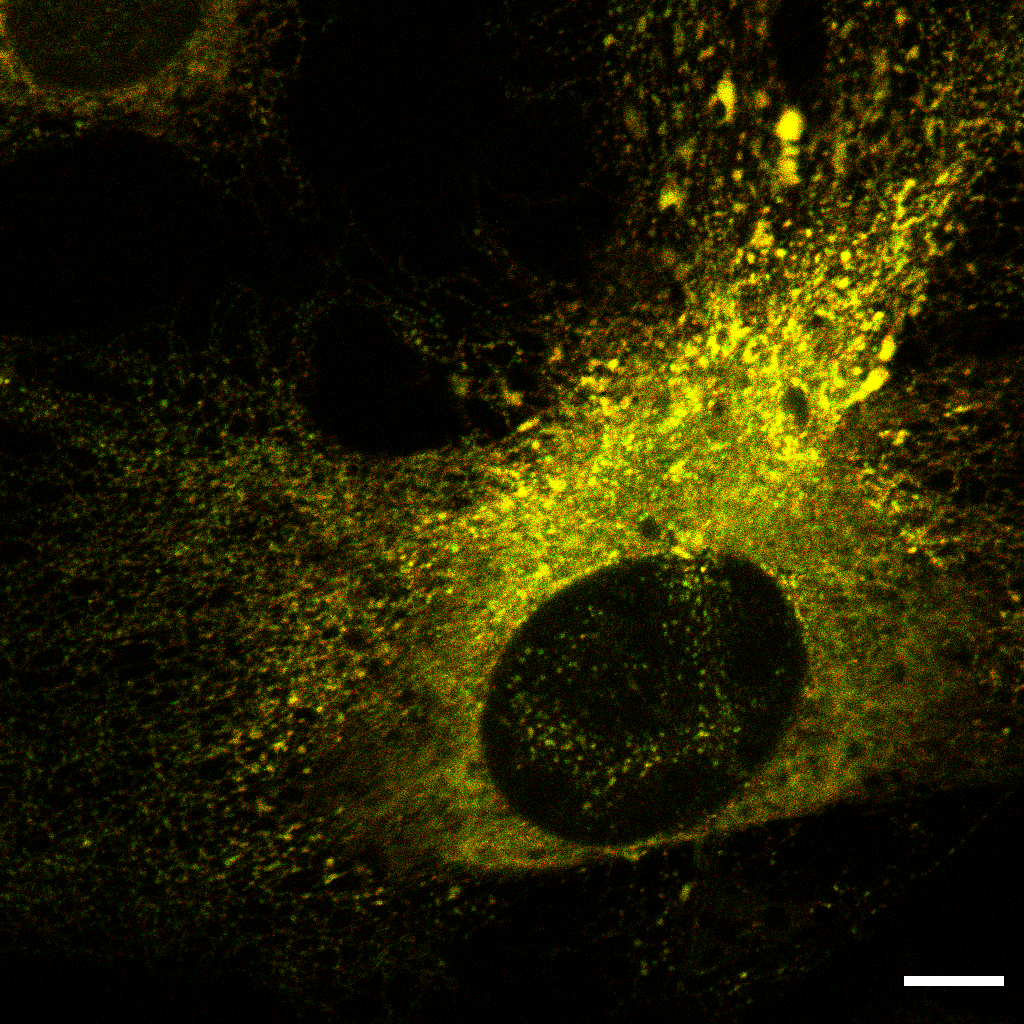

Supplement: Supplementary file 16 — Source data Fig. 2 [file 44318_2024_356_MOESM16_ESM.zip › Figure 2/Fig 2D (day1).tif]

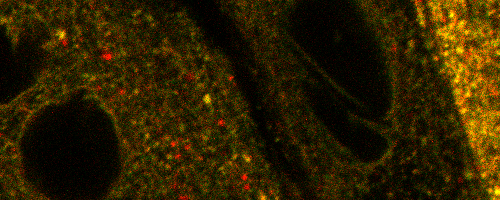

Supplement: Supplementary file 16 — Source data Fig. 2 [file 44318_2024_356_MOESM16_ESM.zip › Figure 2/Fig 2D (day10) inset.tif]

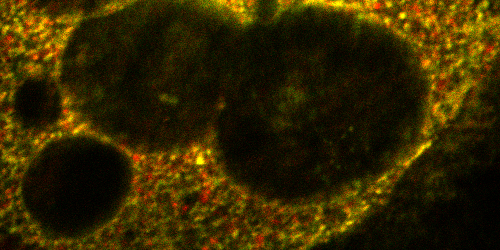

Supplement: Supplementary file 16 — Source data Fig. 2 [file 44318_2024_356_MOESM16_ESM.zip › Figure 2/Fig 2D (day5) inset.tif]

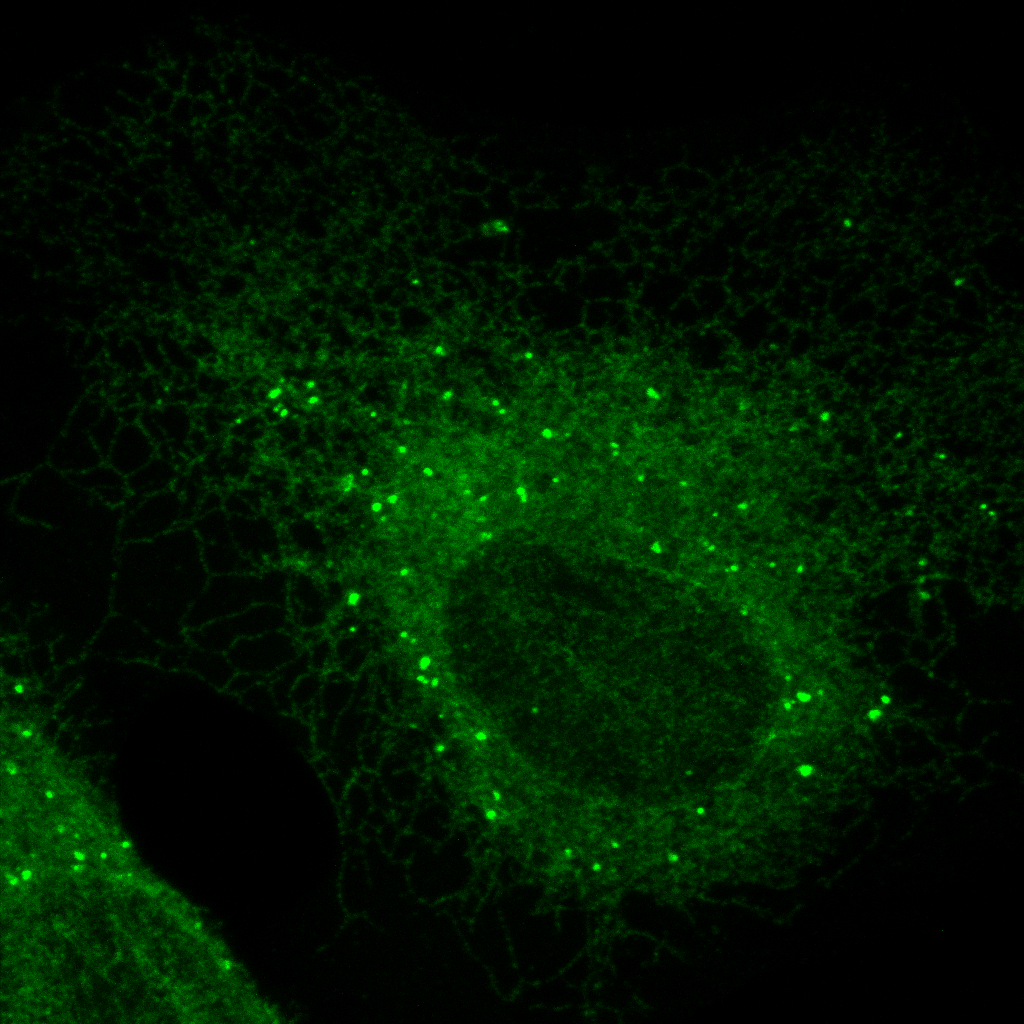

Supplement: Supplementary file 17 — Source data Fig. 3 [file 44318_2024_356_MOESM17_ESM.zip › Figure 3/Fig 3A Fam134b2 HA (CANX).tif]

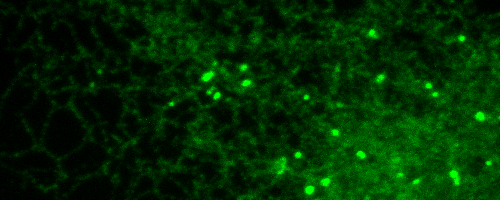

Supplement: Supplementary file 17 — Source data Fig. 3 [file 44318_2024_356_MOESM17_ESM.zip › Figure 3/Fig 3A Fam134b2 HA (CANX) crop.tif]

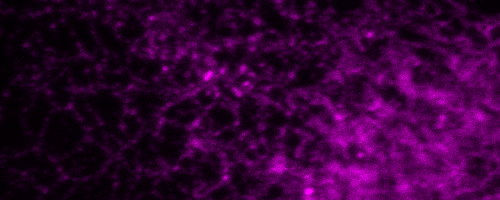

Supplement: Supplementary file 17 — Source data Fig. 3 [file 44318_2024_356_MOESM17_ESM.zip › Figure 3/Fig 3A Fam134b2 Calnexin crop.tif]

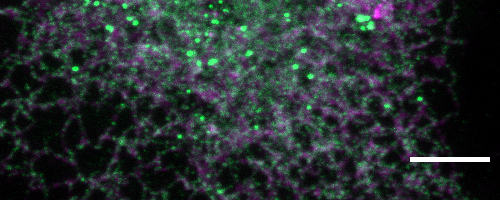

Supplement: Supplementary file 17 — Source data Fig. 3 [file 44318_2024_356_MOESM17_ESM.zip › Figure 3/Fig 3A Fam134b1 Merge HA-CANX crop.tif]

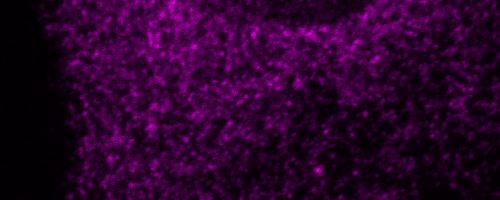

Supplement: Supplementary file 17 — Source data Fig. 3 [file 44318_2024_356_MOESM17_ESM.zip › Figure 3/Fig 3A Fam134b1 Reep5 crop.tif]

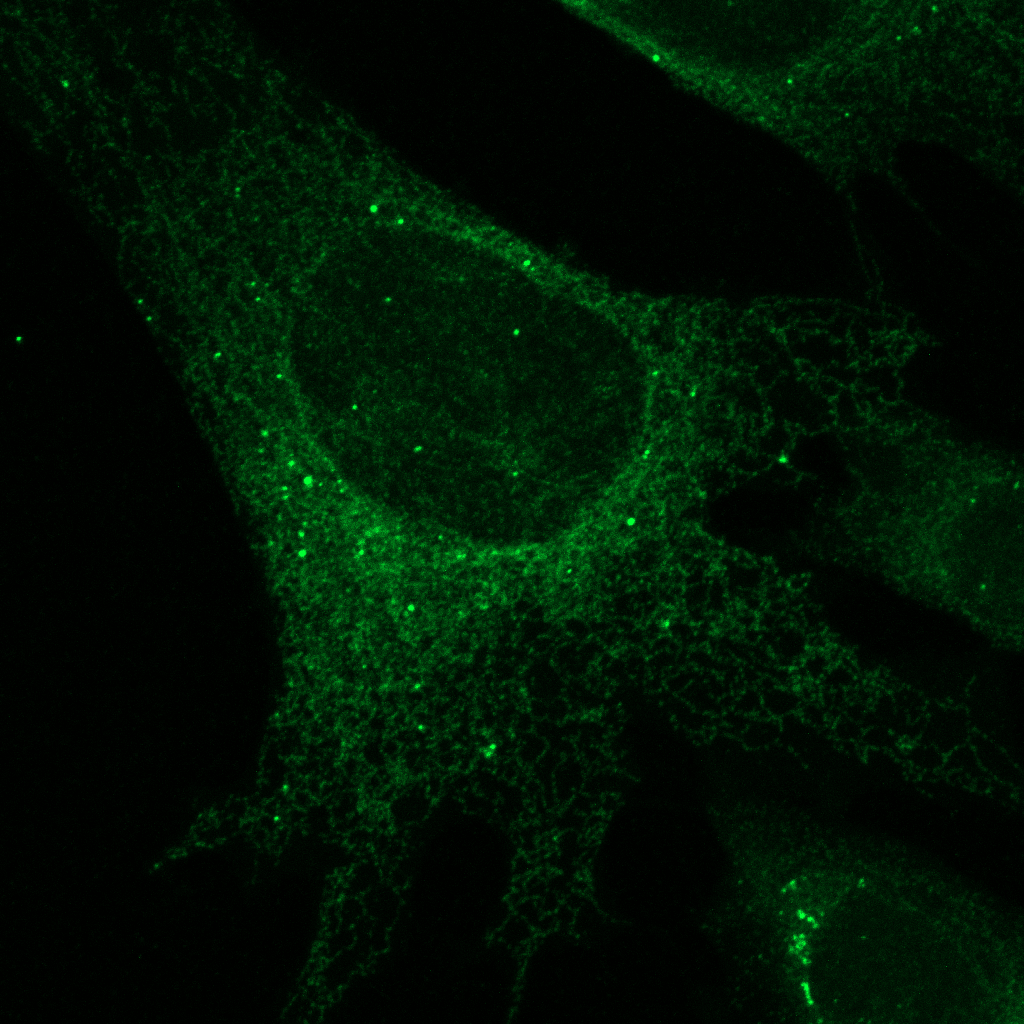

Supplement: Supplementary file 17 — Source data Fig. 3 [file 44318_2024_356_MOESM17_ESM.zip › Figure 3/Fig 3A Fam134b1 HA (Reep5).tif]

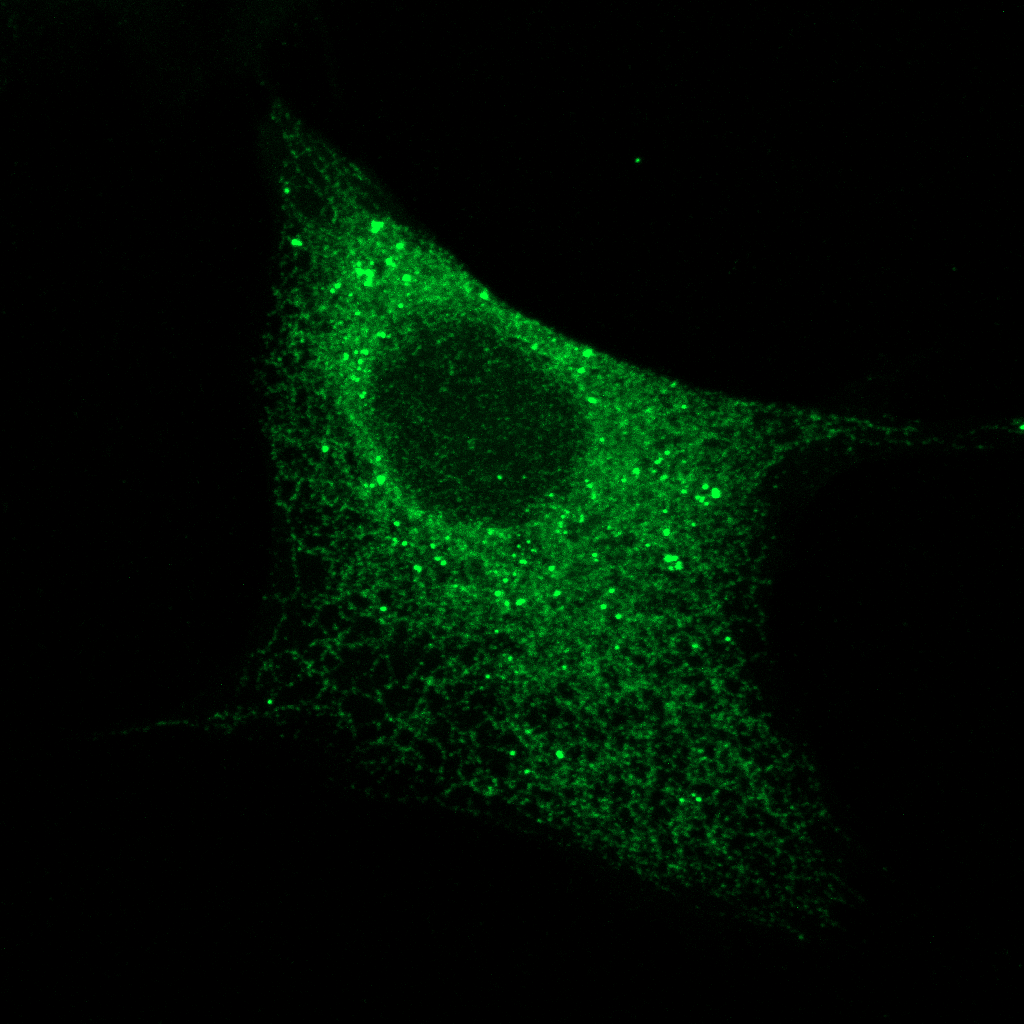

Supplement: Supplementary file 17 — Source data Fig. 3 [file 44318_2024_356_MOESM17_ESM.zip › Figure 3/Fig 3A Fam134b1 HA (CANX).tif]

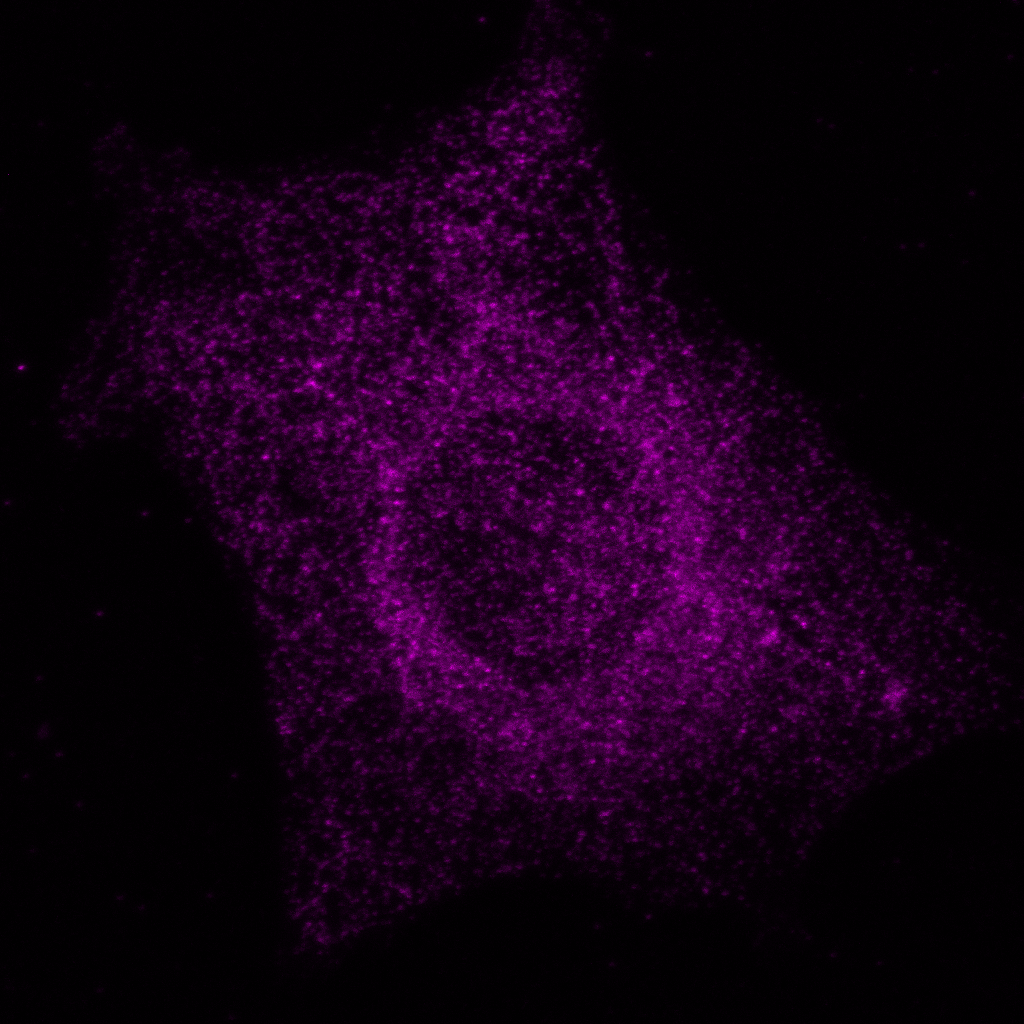

Supplement: Supplementary file 17 — Source data Fig. 3 [file 44318_2024_356_MOESM17_ESM.zip › Figure 3/Fig 3A Fam134b2 Reep5.tif]

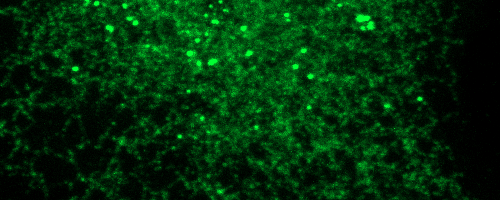

Supplement: Supplementary file 17 — Source data Fig. 3 [file 44318_2024_356_MOESM17_ESM.zip › Figure 3/Fig 3A Fam134b1 HA (CANX) crop.tif]

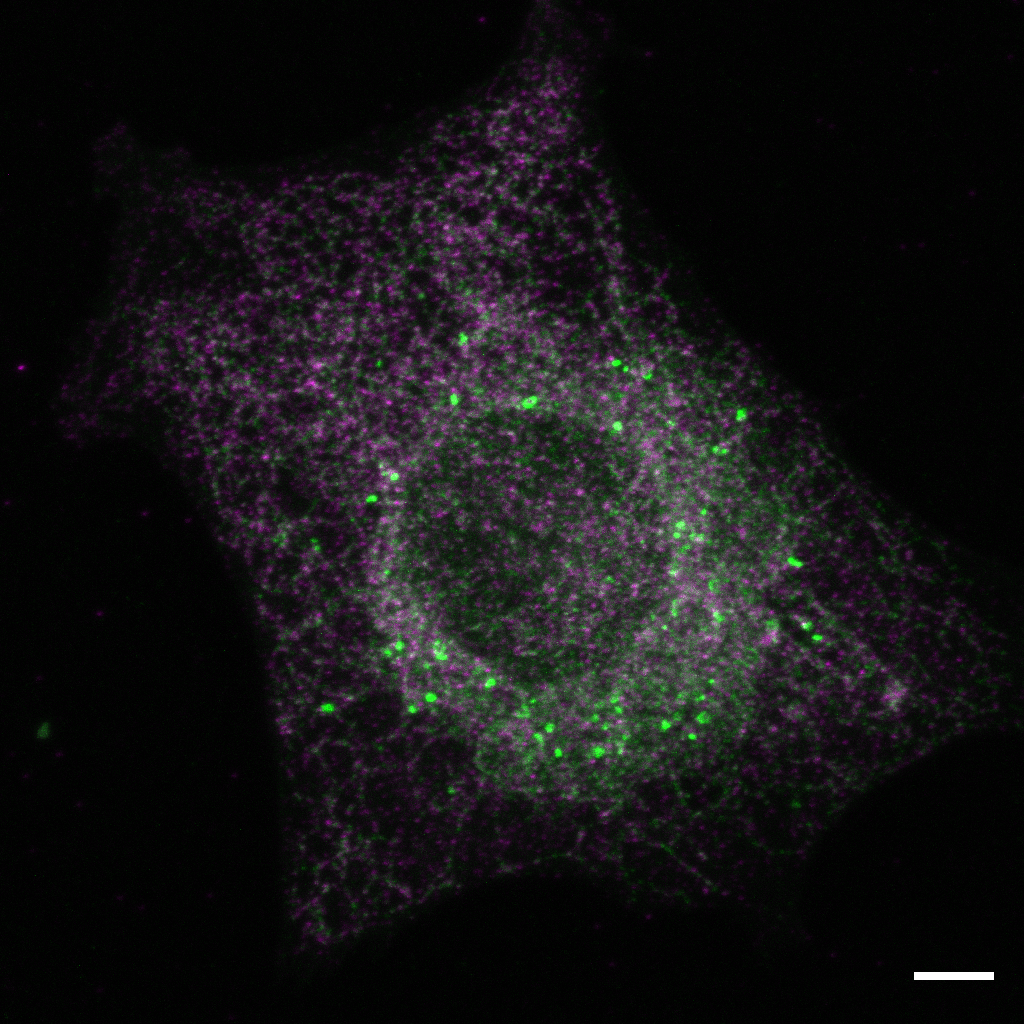

Supplement: Supplementary file 17 — Source data Fig. 3 [file 44318_2024_356_MOESM17_ESM.zip › Figure 3/Fig 3A Fam134b2 Merge Reep5-HA.tif]

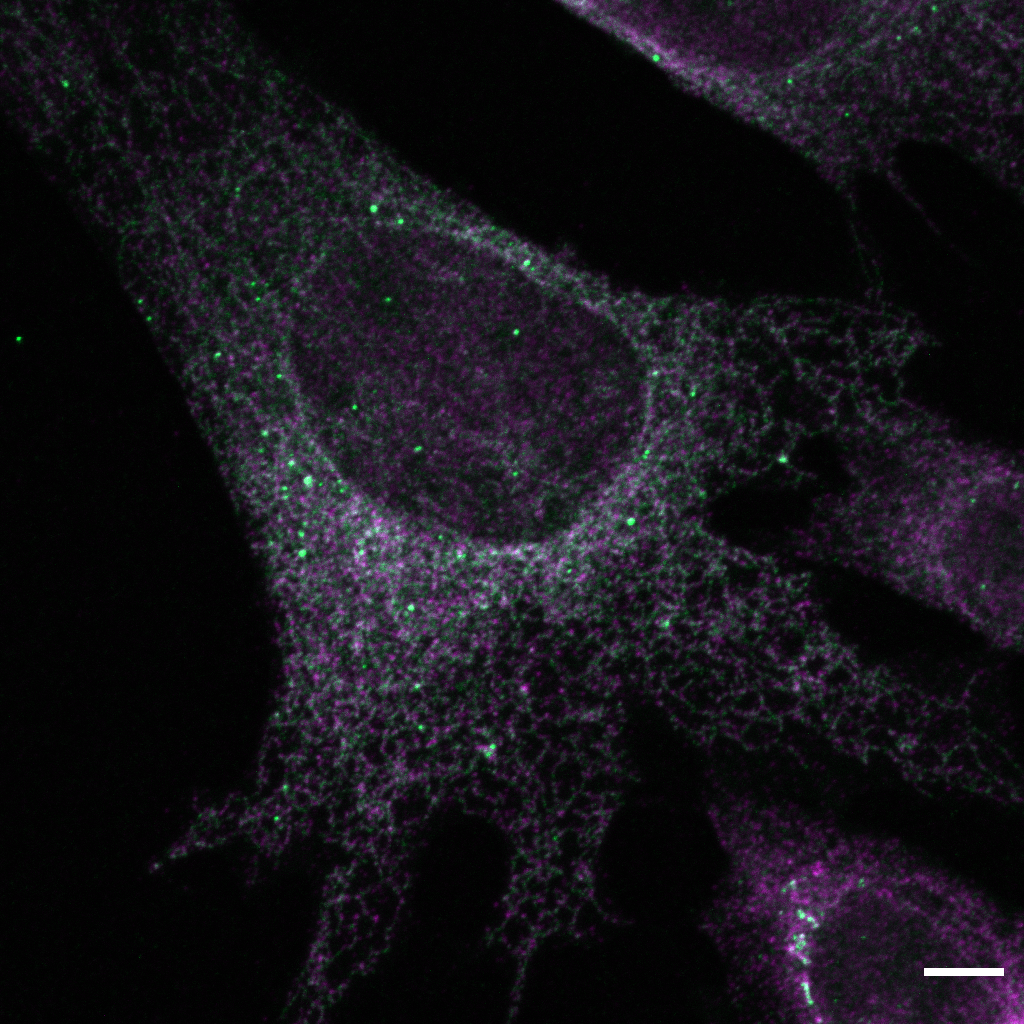

Supplement: Supplementary file 17 — Source data Fig. 3 [file 44318_2024_356_MOESM17_ESM.zip › Figure 3/Fig 3A Fam134b1 Merge HA-Reep5.tif]

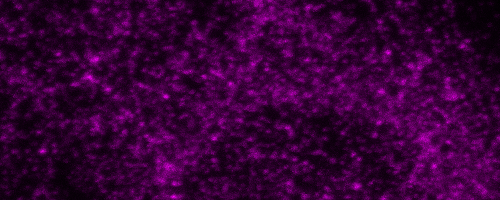

Supplement: Supplementary file 17 — Source data Fig. 3 [file 44318_2024_356_MOESM17_ESM.zip › Figure 3/Fig 3A Fam134b2 Reep5 crop.tif]

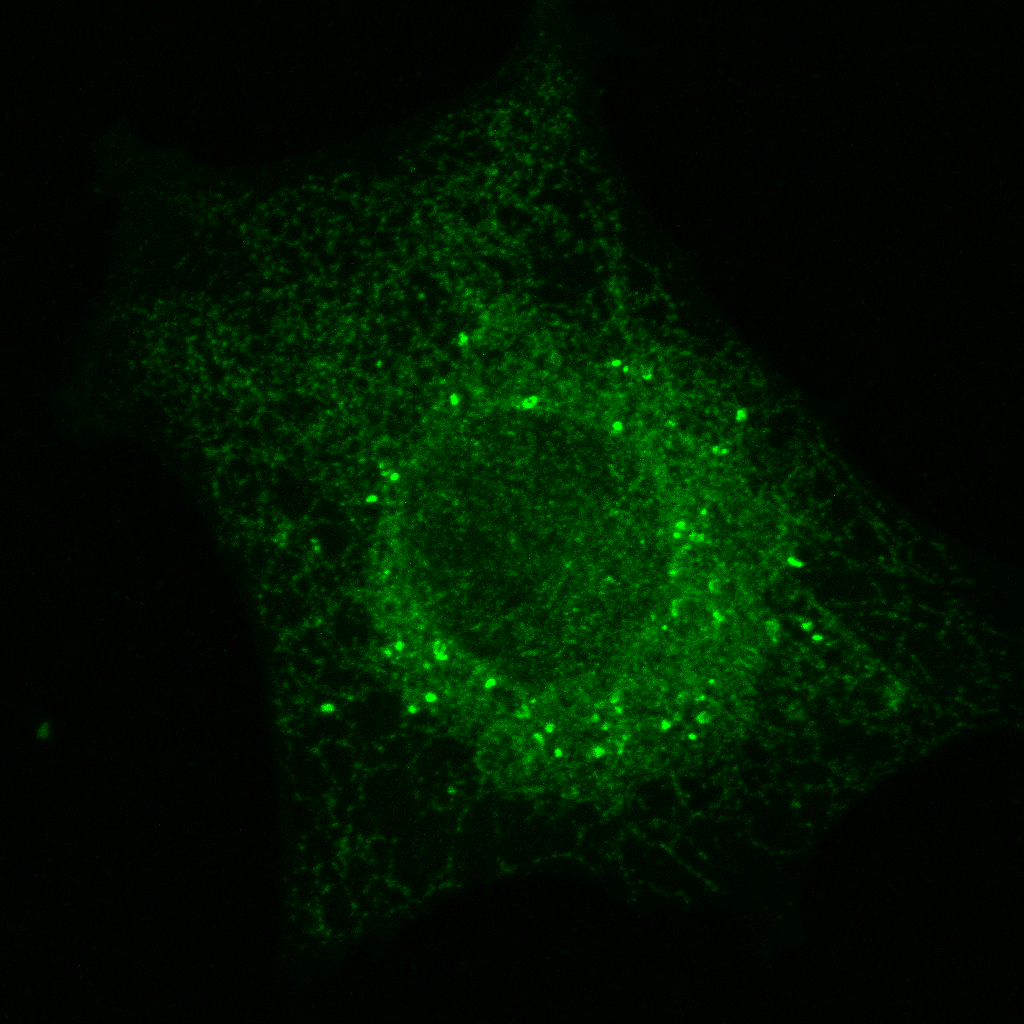

Supplement: Supplementary file 17 — Source data Fig. 3 [file 44318_2024_356_MOESM17_ESM.zip › Figure 3/Fig 3A Fam134b2 HA (Reep5).tif]

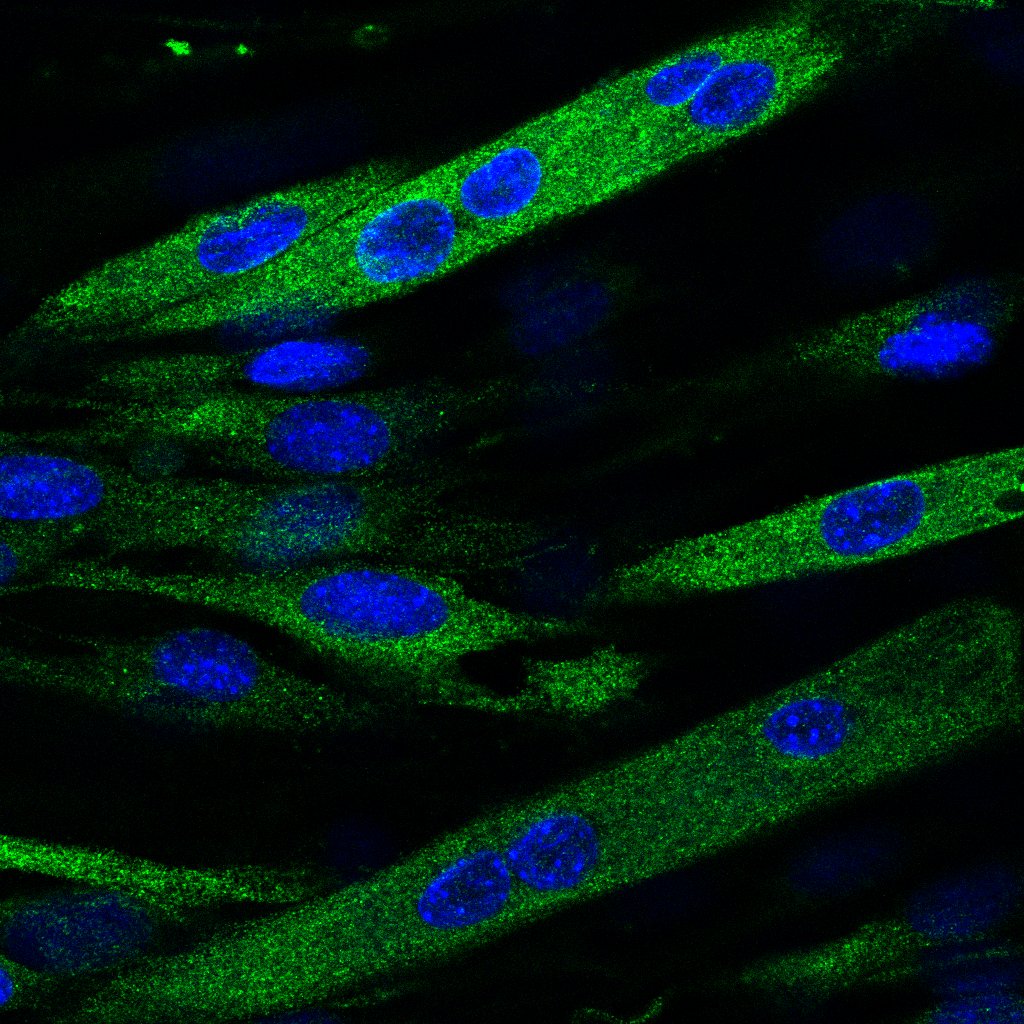

Supplement: Supplementary file 17 — Source data Fig. 3 [file 44318_2024_356_MOESM17_ESM.zip › Figure 3/Fig 3J KDEL.jpg]

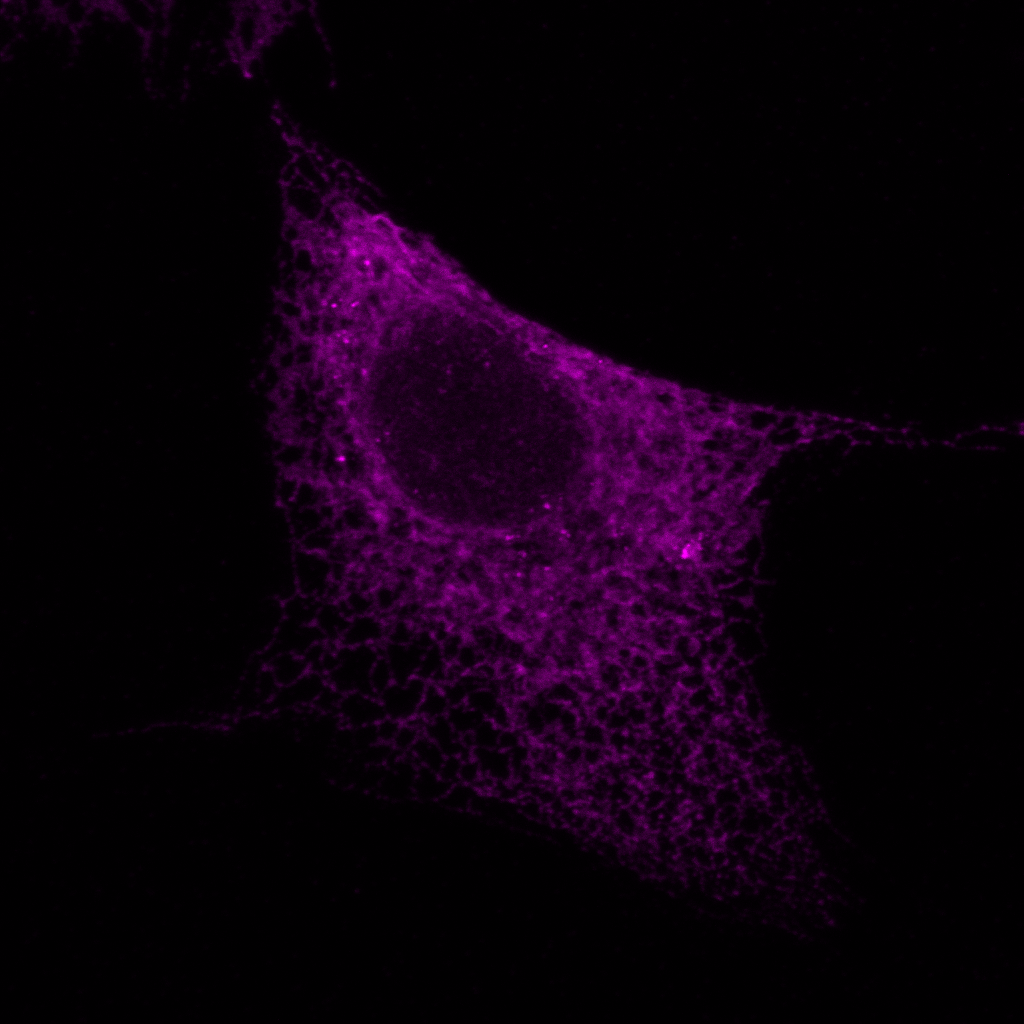

Supplement: Supplementary file 17 — Source data Fig. 3 [file 44318_2024_356_MOESM17_ESM.zip › Figure 3/Fig 3A Fam134b1 Calnexin.tif]

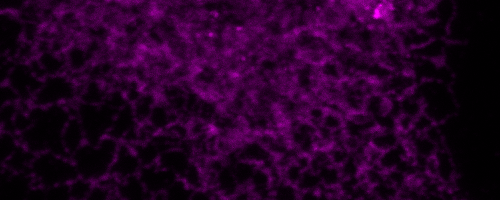

Supplement: Supplementary file 17 — Source data Fig. 3 [file 44318_2024_356_MOESM17_ESM.zip › Figure 3/Fig 3A Fam134b1 Calnexin crop.tif]

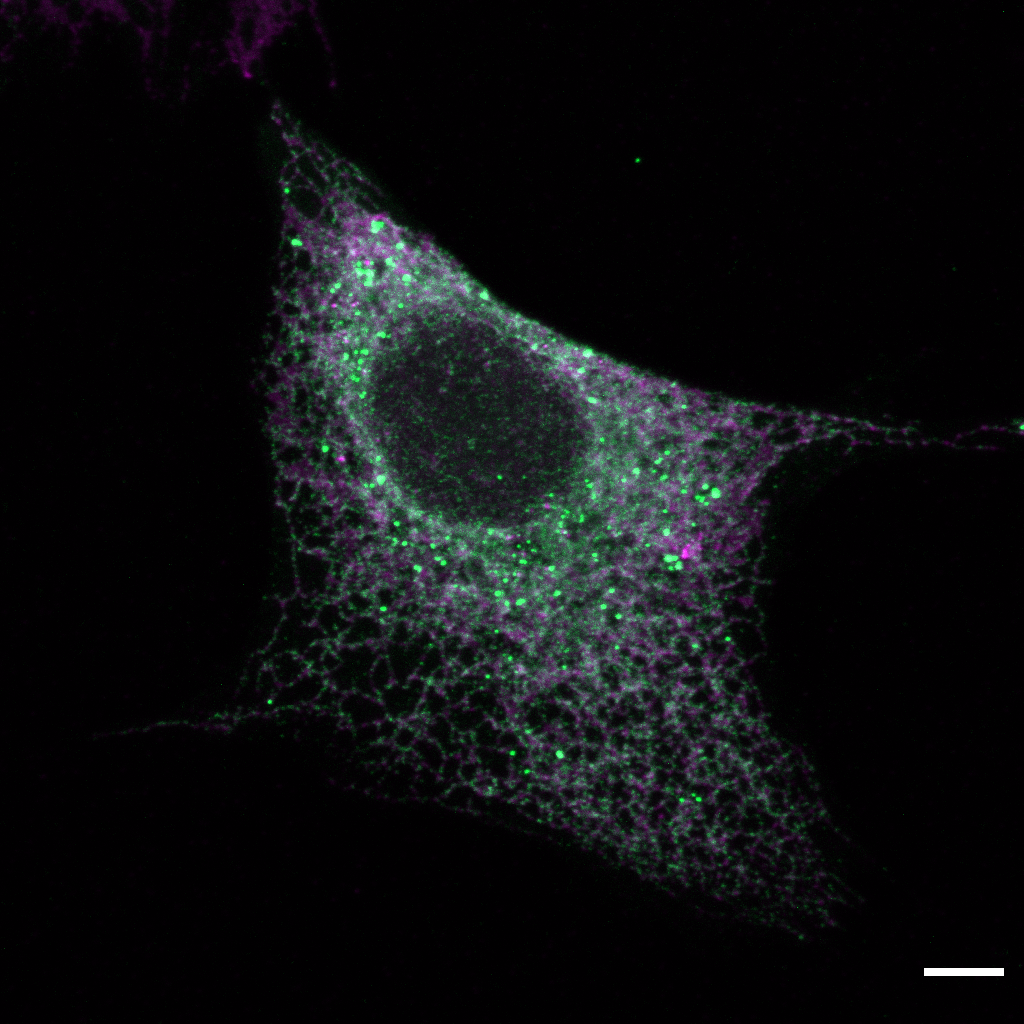

Supplement: Supplementary file 17 — Source data Fig. 3 [file 44318_2024_356_MOESM17_ESM.zip › Figure 3/Fig 3A Fam134b1 Merge HA-Calnexin.tif]

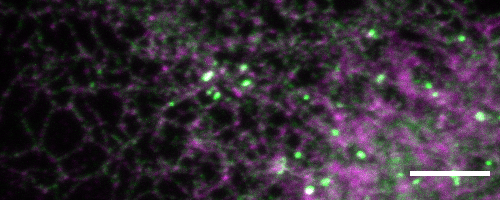

Supplement: Supplementary file 17 — Source data Fig. 3 [file 44318_2024_356_MOESM17_ESM.zip › Figure 3/Fig 3A Fam134b2 Merge HA-Calnexin crop.tif]

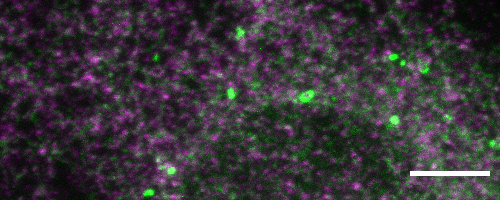

Supplement: Supplementary file 17 — Source data Fig. 3 [file 44318_2024_356_MOESM17_ESM.zip › Figure 3/Fig 3A Fam134b2 Merge HA-Reep5 crop.tif]

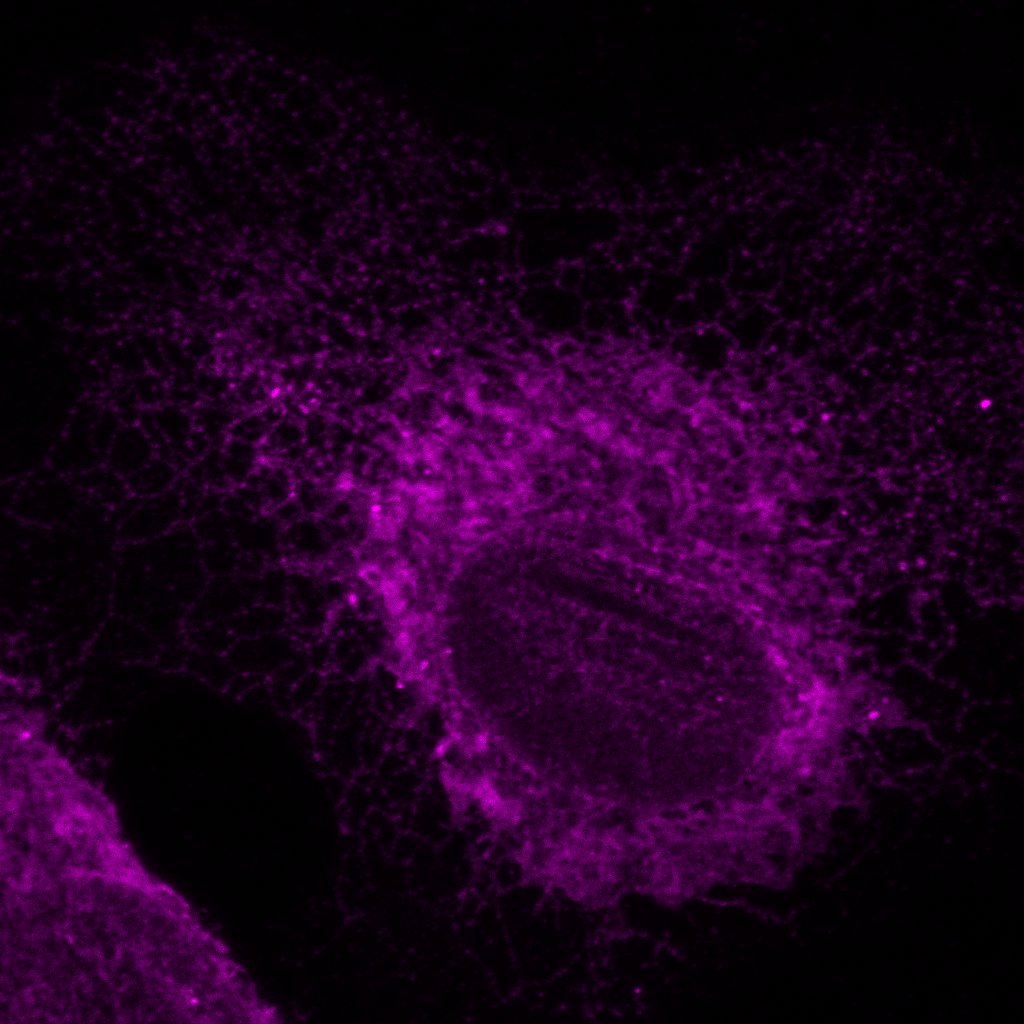

Supplement: Supplementary file 17 — Source data Fig. 3 [file 44318_2024_356_MOESM17_ESM.zip › Figure 3/Fig 3A Fam134b2 Calnexin.tif]

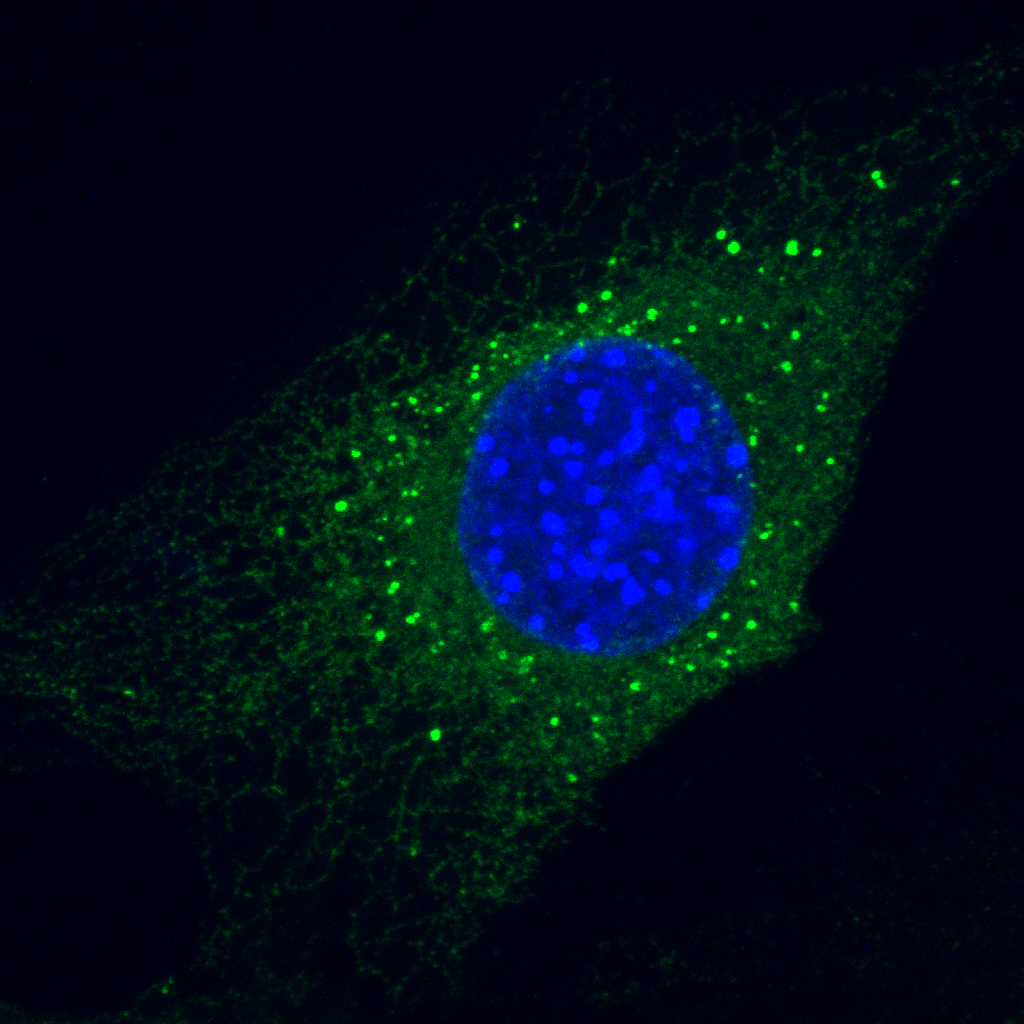

Supplement: Supplementary file 17 — Source data Fig. 3 [file 44318_2024_356_MOESM17_ESM.zip › Figure 3/Fig 3H Fam134b2 24h.tif]

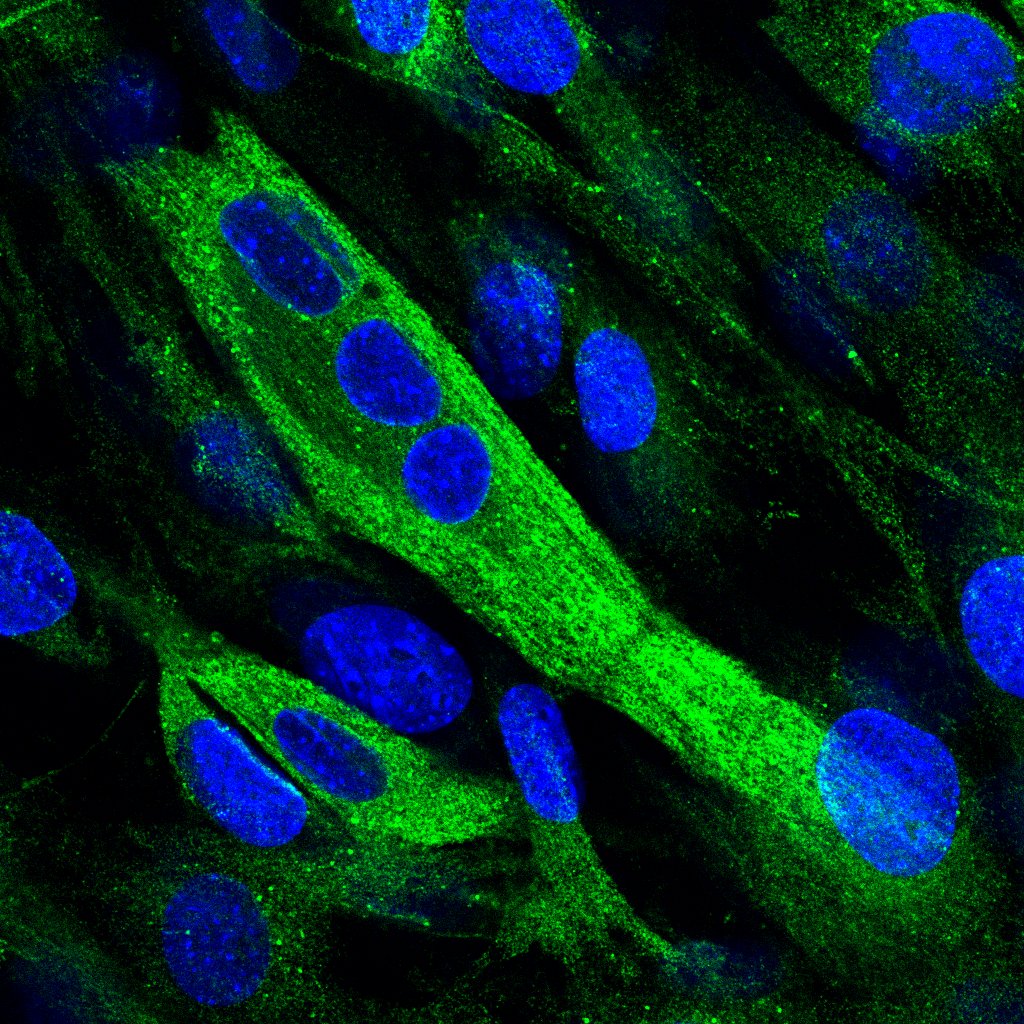

Supplement: Supplementary file 17 — Source data Fig. 3 [file 44318_2024_356_MOESM17_ESM.zip › Figure 3/Fig 3J Fam134b1.jpg]

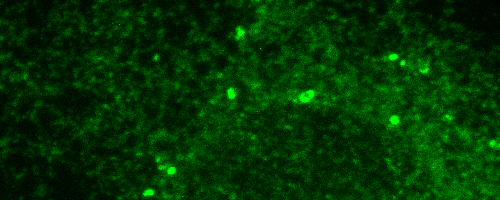

Supplement: Supplementary file 17 — Source data Fig. 3 [file 44318_2024_356_MOESM17_ESM.zip › Figure 3/Fig 3A Fam134b2 HA (Reep5) crop.tif]

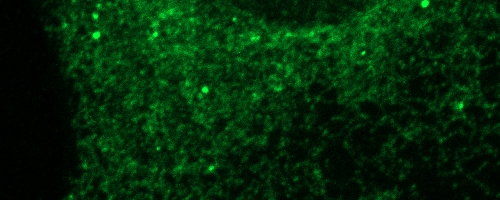

Supplement: Supplementary file 17 — Source data Fig. 3 [file 44318_2024_356_MOESM17_ESM.zip › Figure 3/Fig 3A Fam134b1 HA (Reep5) crop.tif]

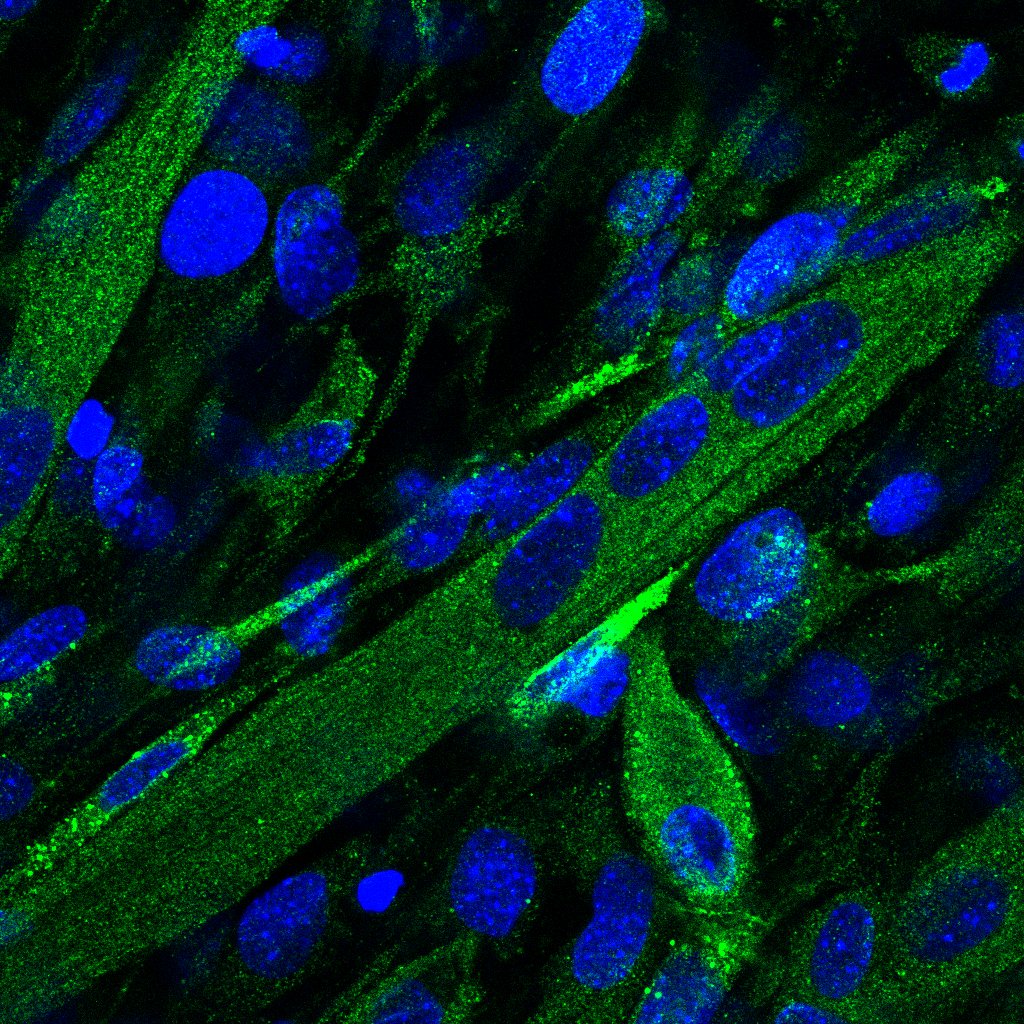

Supplement: Supplementary file 17 — Source data Fig. 3 [file 44318_2024_356_MOESM17_ESM.zip › Figure 3/Fig 3J Fam134b2.jpg]

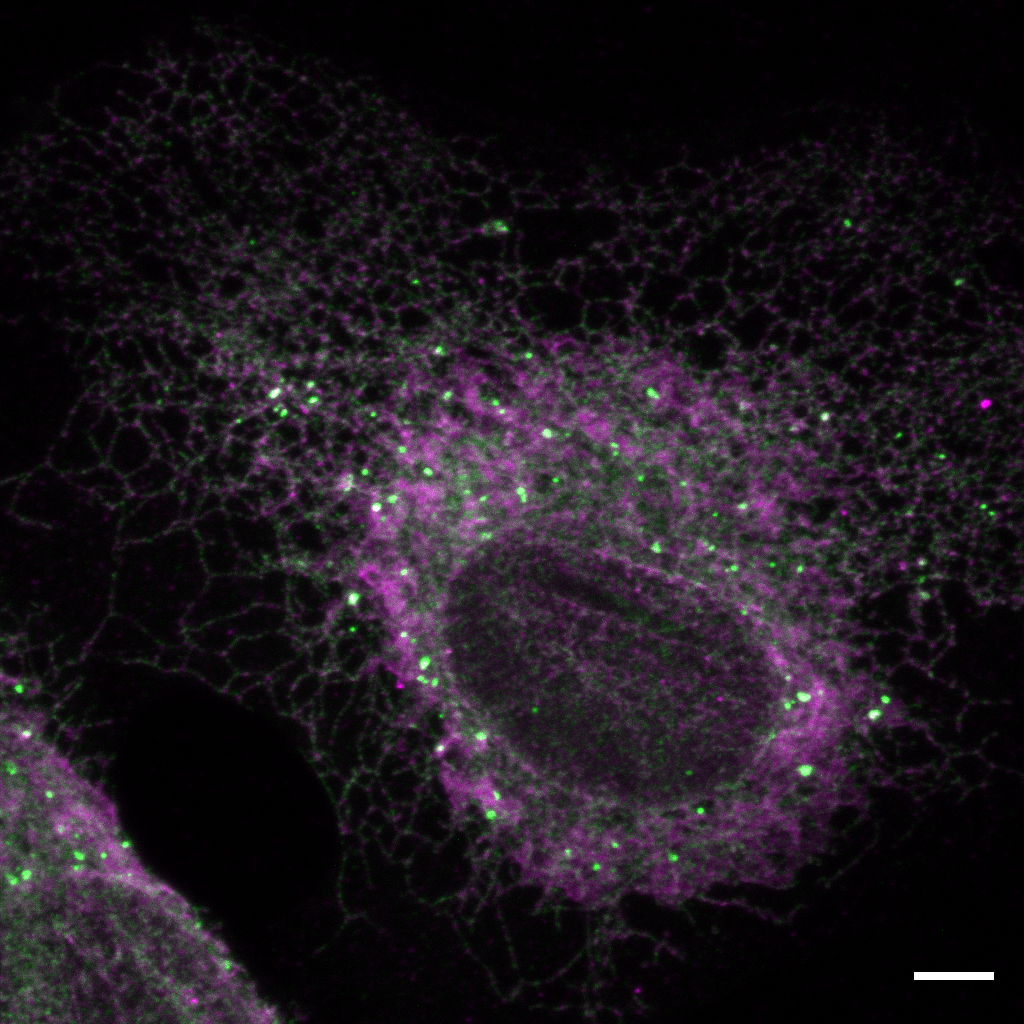

Supplement: Supplementary file 17 — Source data Fig. 3 [file 44318_2024_356_MOESM17_ESM.zip › Figure 3/Fig 3A Fam134b2 Merge Calnexin-HA.tif]

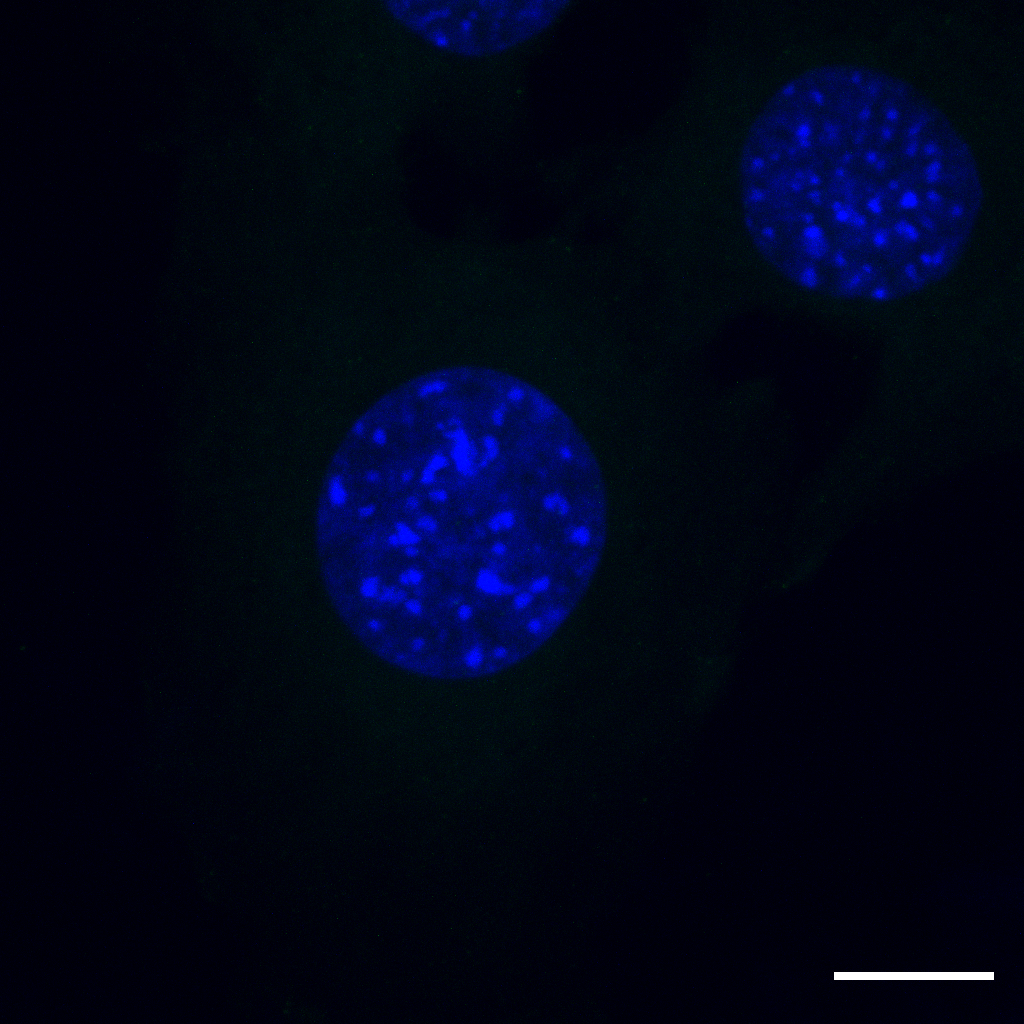

Supplement: Supplementary file 17 — Source data Fig. 3 [file 44318_2024_356_MOESM17_ESM.zip › Figure 3/Fig 3H no doxi Fam134b2.tif]

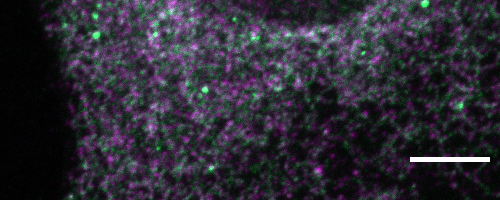

Supplement: Supplementary file 17 — Source data Fig. 3 [file 44318_2024_356_MOESM17_ESM.zip › Figure 3/Fig 3A Fam134b1 Merge HA-Reep5 crop.tif]

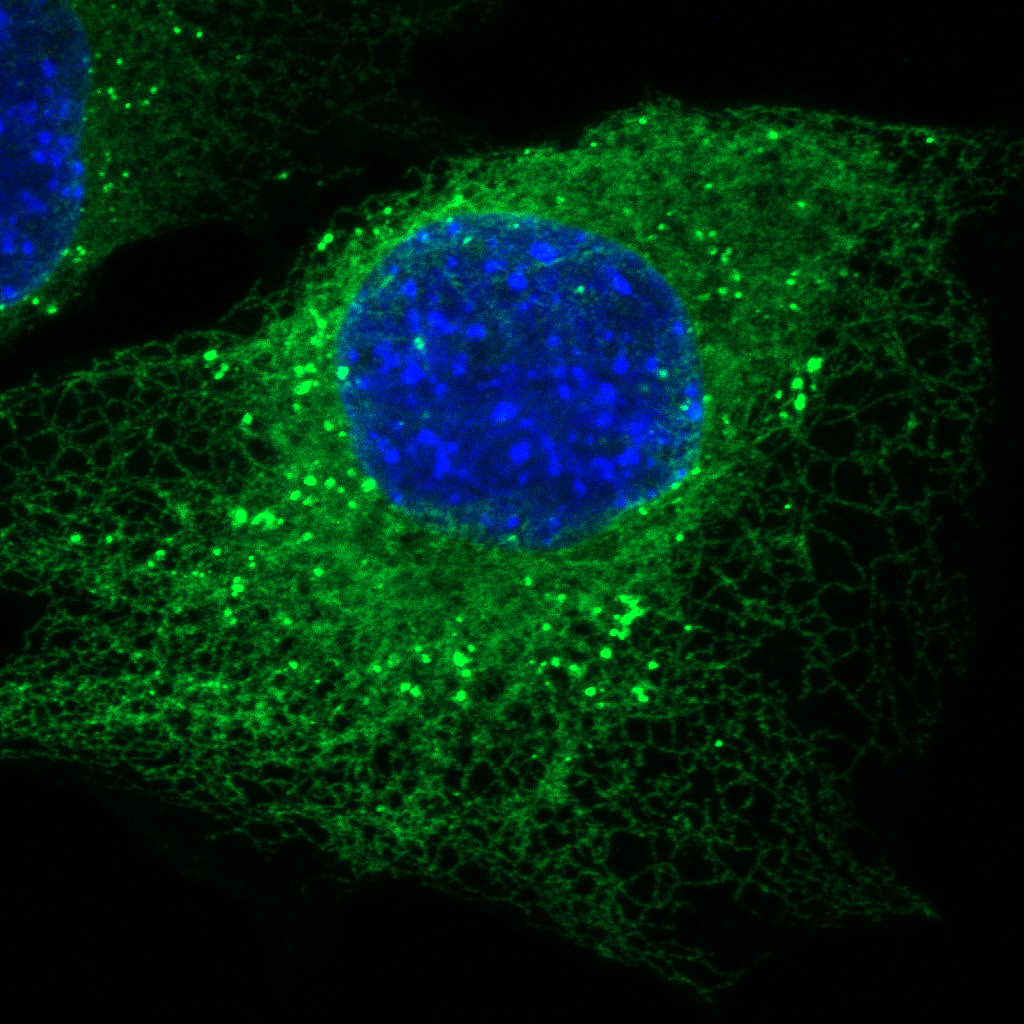

Supplement: Supplementary file 17 — Source data Fig. 3 [file 44318_2024_356_MOESM17_ESM.zip › Figure 3/Fig 3H Fam134b1 24h.tif]

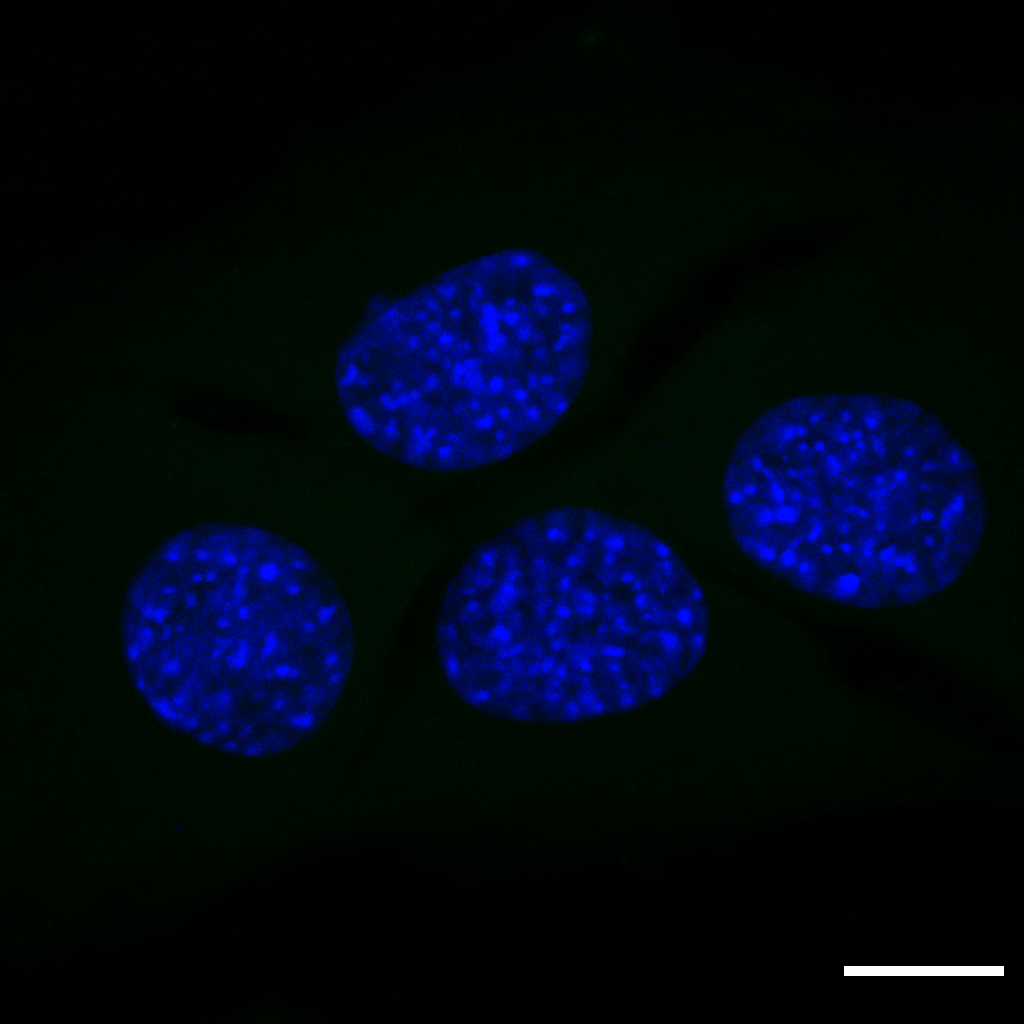

Supplement: Supplementary file 17 — Source data Fig. 3 [file 44318_2024_356_MOESM17_ESM.zip › Figure 3/Fig 3H no dox Fam134b1.tif]

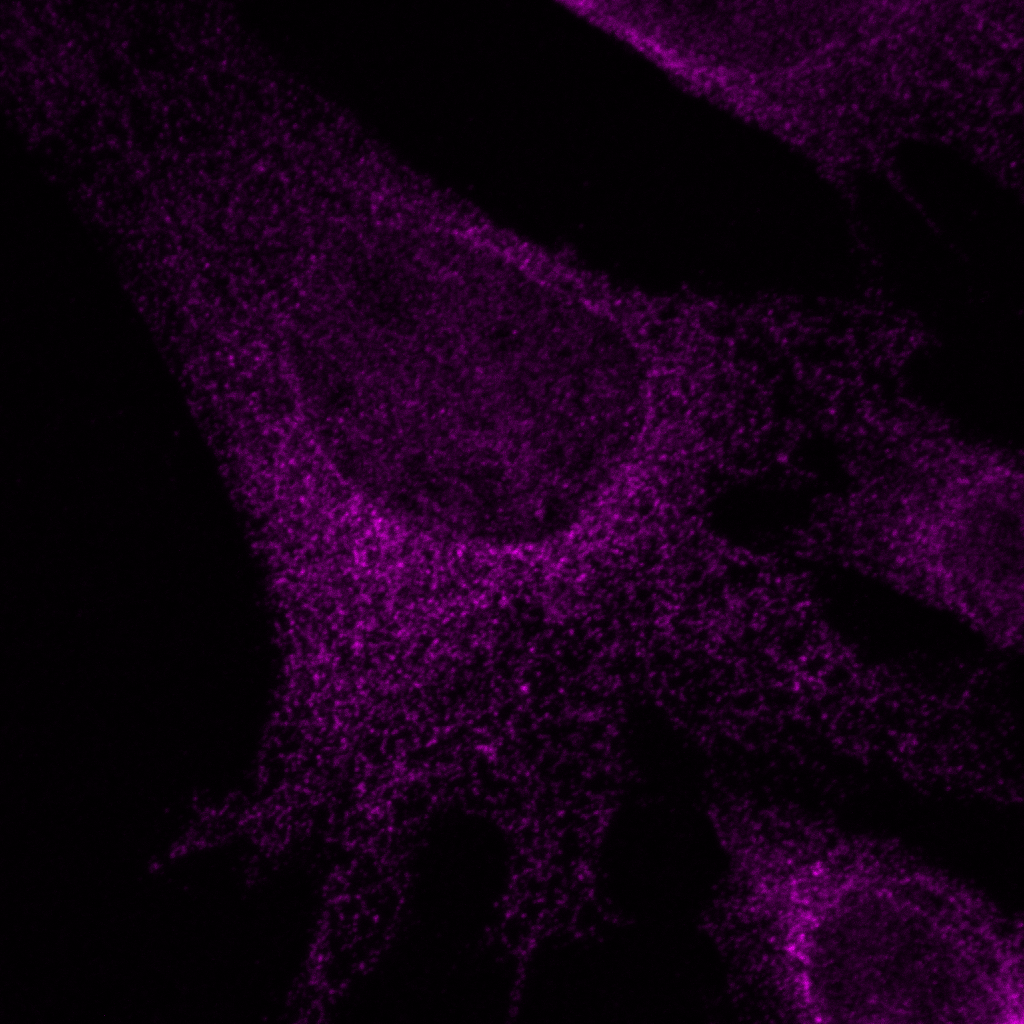

Supplement: Supplementary file 17 — Source data Fig. 3 [file 44318_2024_356_MOESM17_ESM.zip › Figure 3/Fig 3A Fam134b1 Reep5.tif]

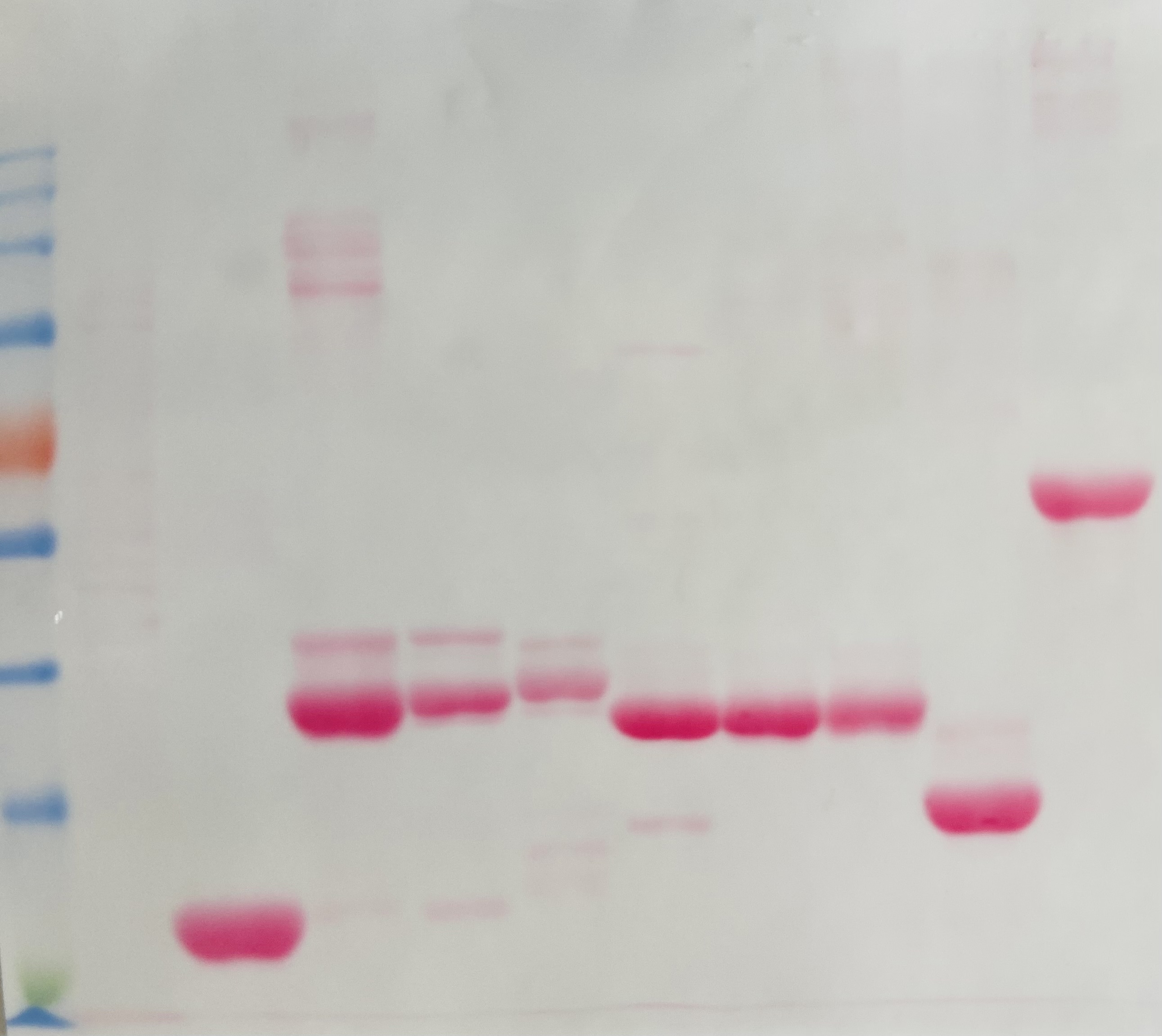

Supplement: Supplementary file 18 — Source data Fig. 4 [file 44318_2024_356_MOESM18_ESM.zip › Figure 4/Fig 4A Ponceau.jpg]

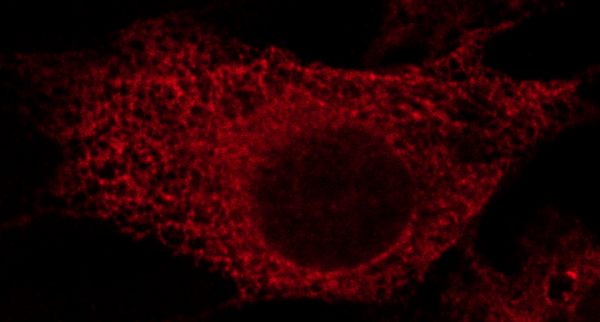

Supplement: Supplementary file 18 — Source data Fig. 4 [file 44318_2024_356_MOESM18_ESM.zip › Figure 4/Fig 4D HA Fam134b2LIR.tif]

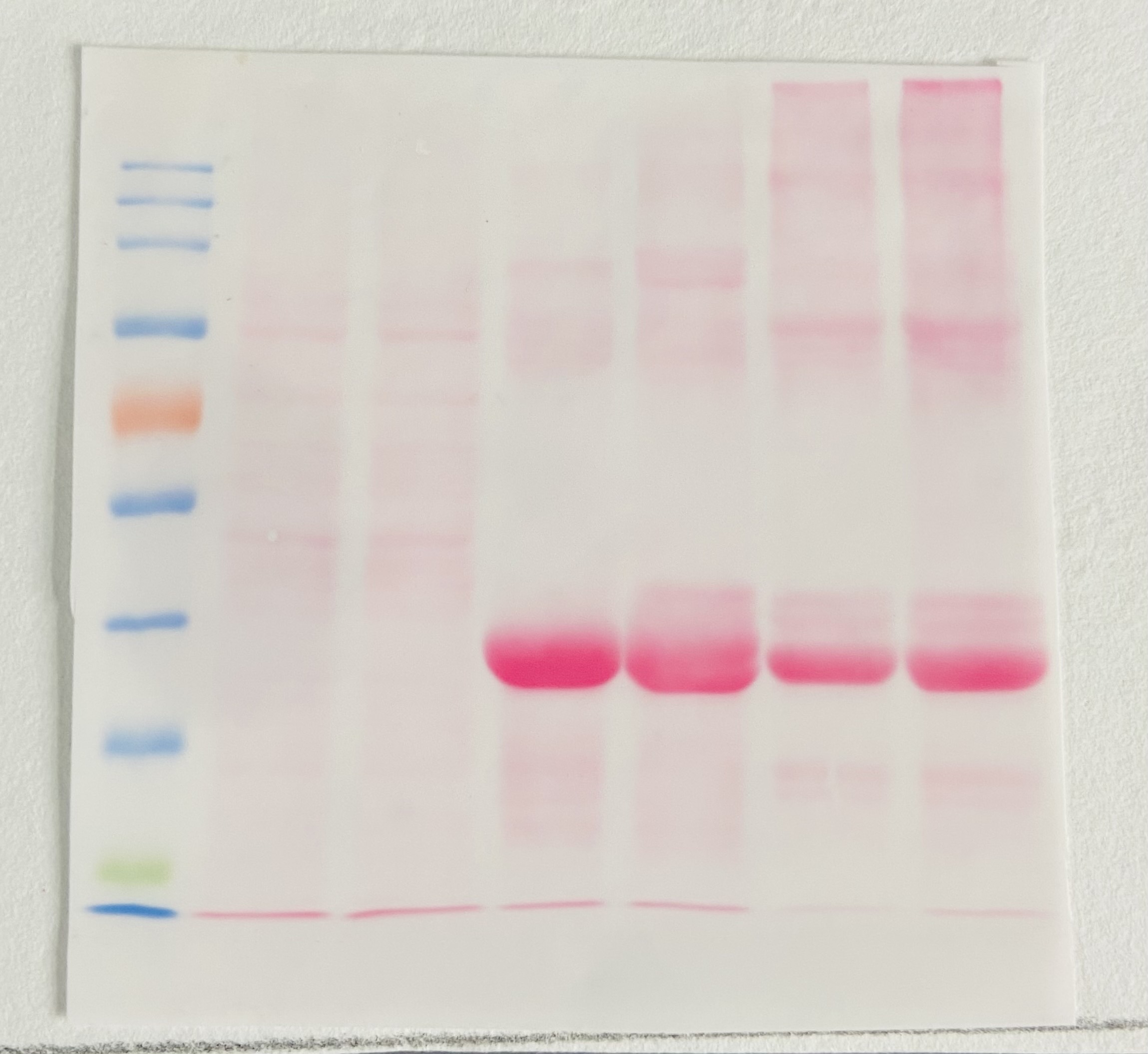

Supplement: Supplementary file 18 — Source data Fig. 4 [file 44318_2024_356_MOESM18_ESM.zip › Figure 4/Fig 4C Ponceau.jpeg]

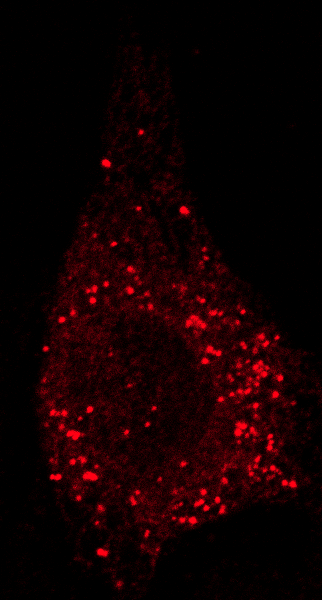

Supplement: Supplementary file 18 — Source data Fig. 4 [file 44318_2024_356_MOESM18_ESM.zip › Figure 4/Fig 4D HA Fam134b2.tif]

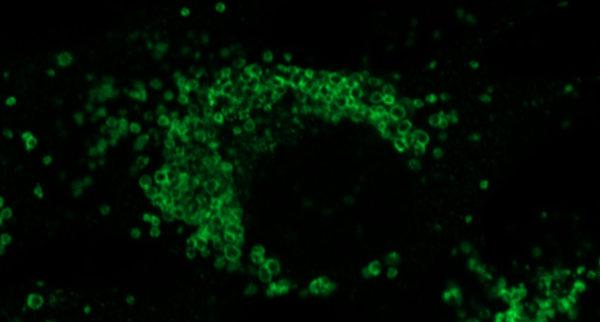

Supplement: Supplementary file 18 — Source data Fig. 4 [file 44318_2024_356_MOESM18_ESM.zip › Figure 4/Fig 4D LAMP1 Fam134b2LIR.tif]

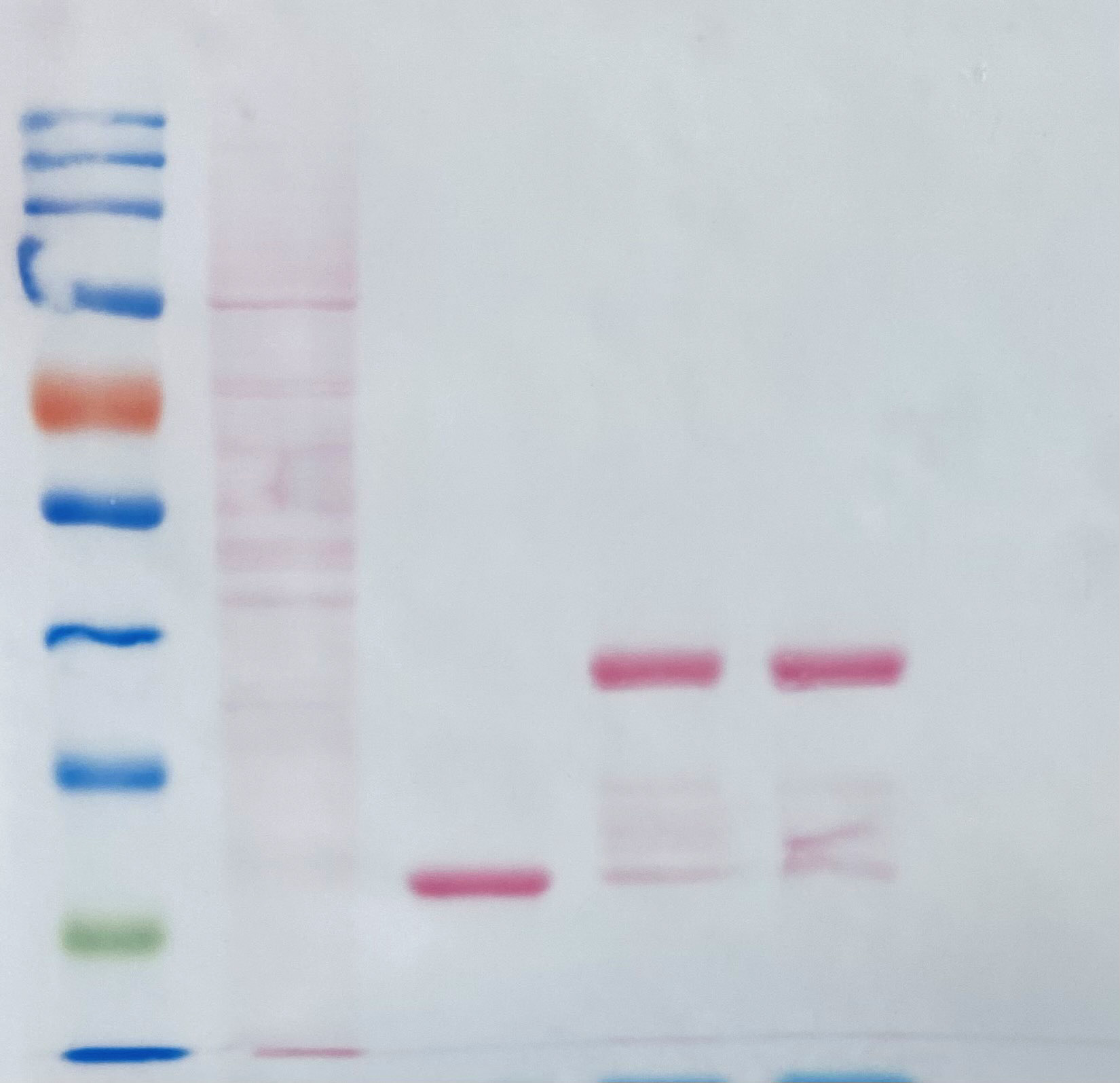

Supplement: Supplementary file 18 — Source data Fig. 4 [file 44318_2024_356_MOESM18_ESM.zip › Figure 4/Fig 4B Ponceau.jpg]

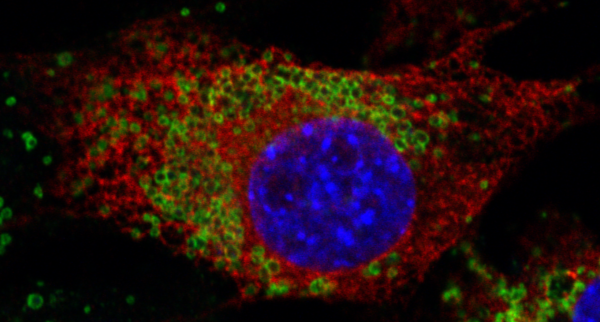

Supplement: Supplementary file 18 — Source data Fig. 4 [file 44318_2024_356_MOESM18_ESM.zip › Figure 4/Fig 4D Merge Fam134b2LIR.tif]

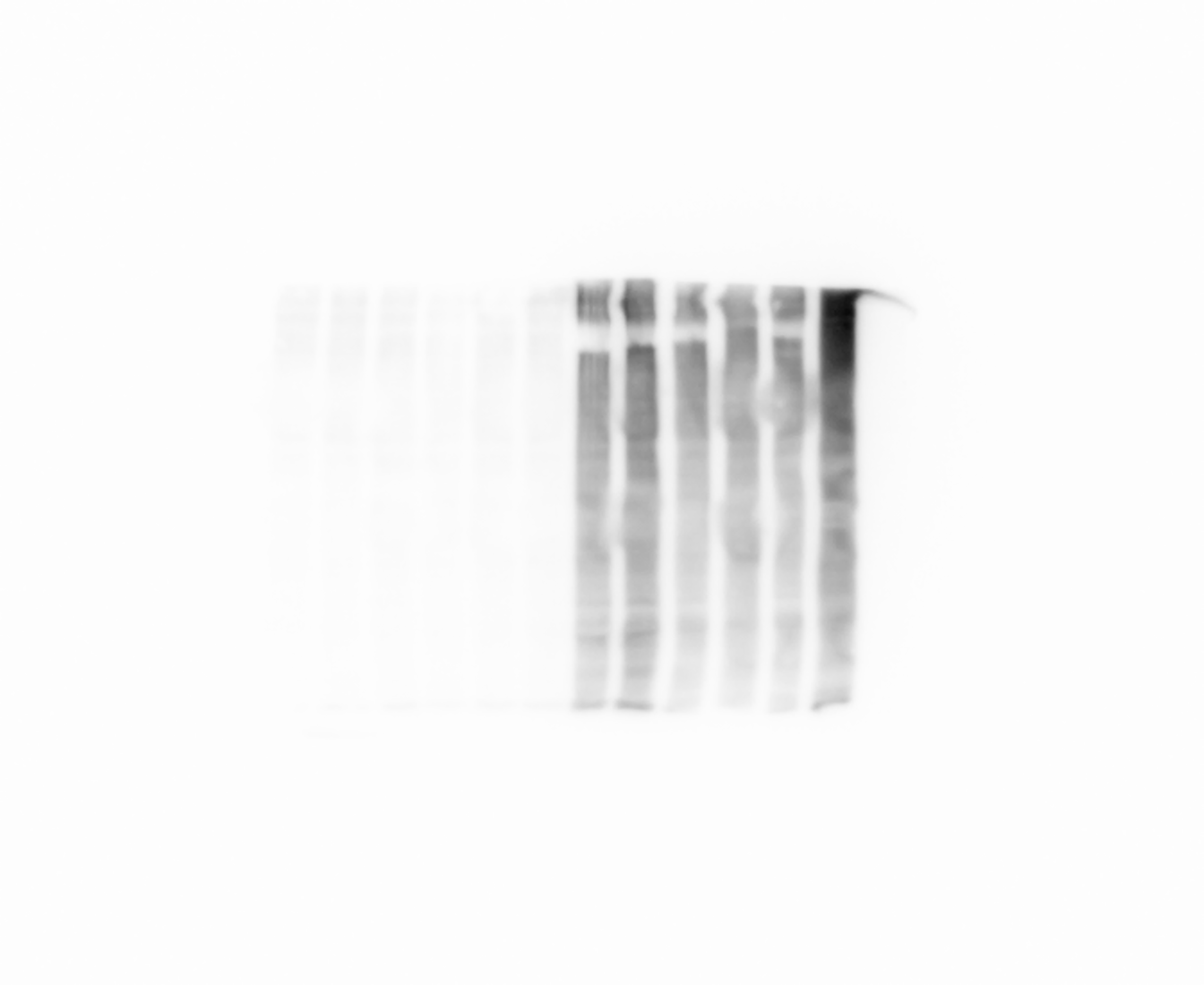

Supplement: Supplementary file 18 — Source data Fig. 4 [file 44318_2024_356_MOESM18_ESM.zip › Figure 4/Fig 4I Ub.Tif]

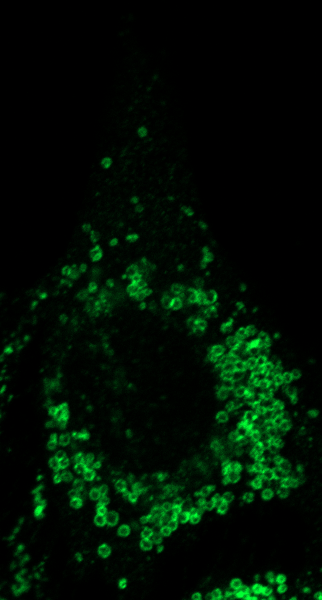

Supplement: Supplementary file 18 — Source data Fig. 4 [file 44318_2024_356_MOESM18_ESM.zip › Figure 4/Fig 4D LAMP1 Fam134b2.tif]

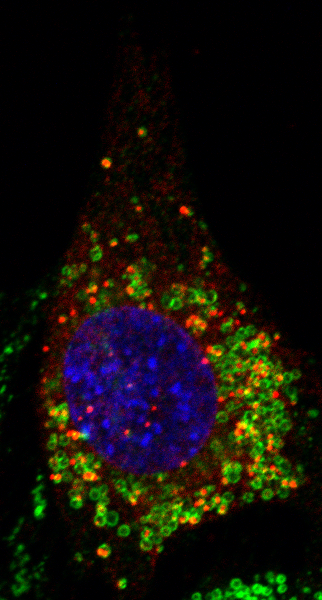

Supplement: Supplementary file 18 — Source data Fig. 4 [file 44318_2024_356_MOESM18_ESM.zip › Figure 4/Fig 4D Merge Fam134b2.tif]

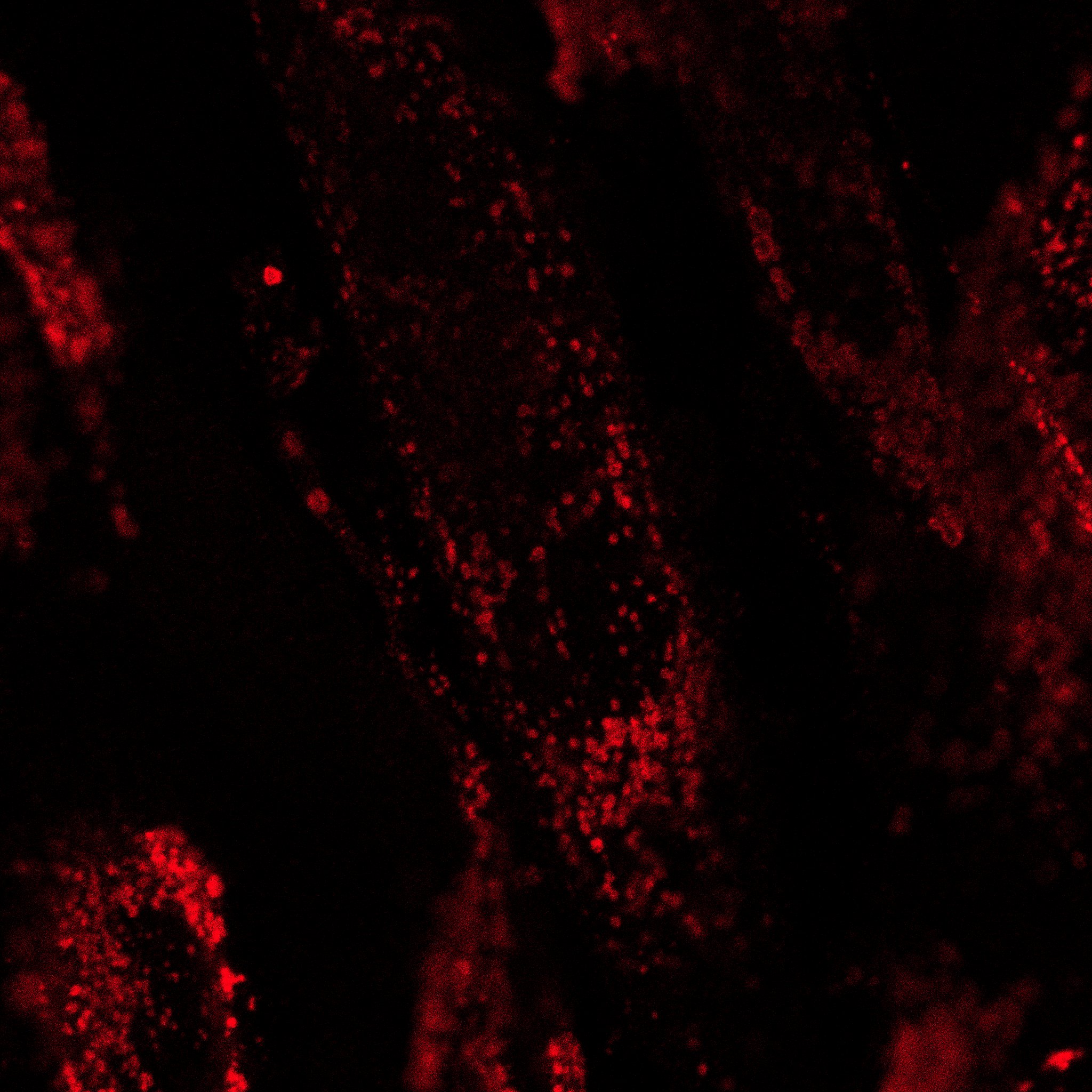

Supplement: Supplementary file 20 — Source data Fig. 6 [file 44318_2024_356_MOESM20_ESM.zip › Figure 6/Fig 6D Fam134b KO Lamp1.jpg]

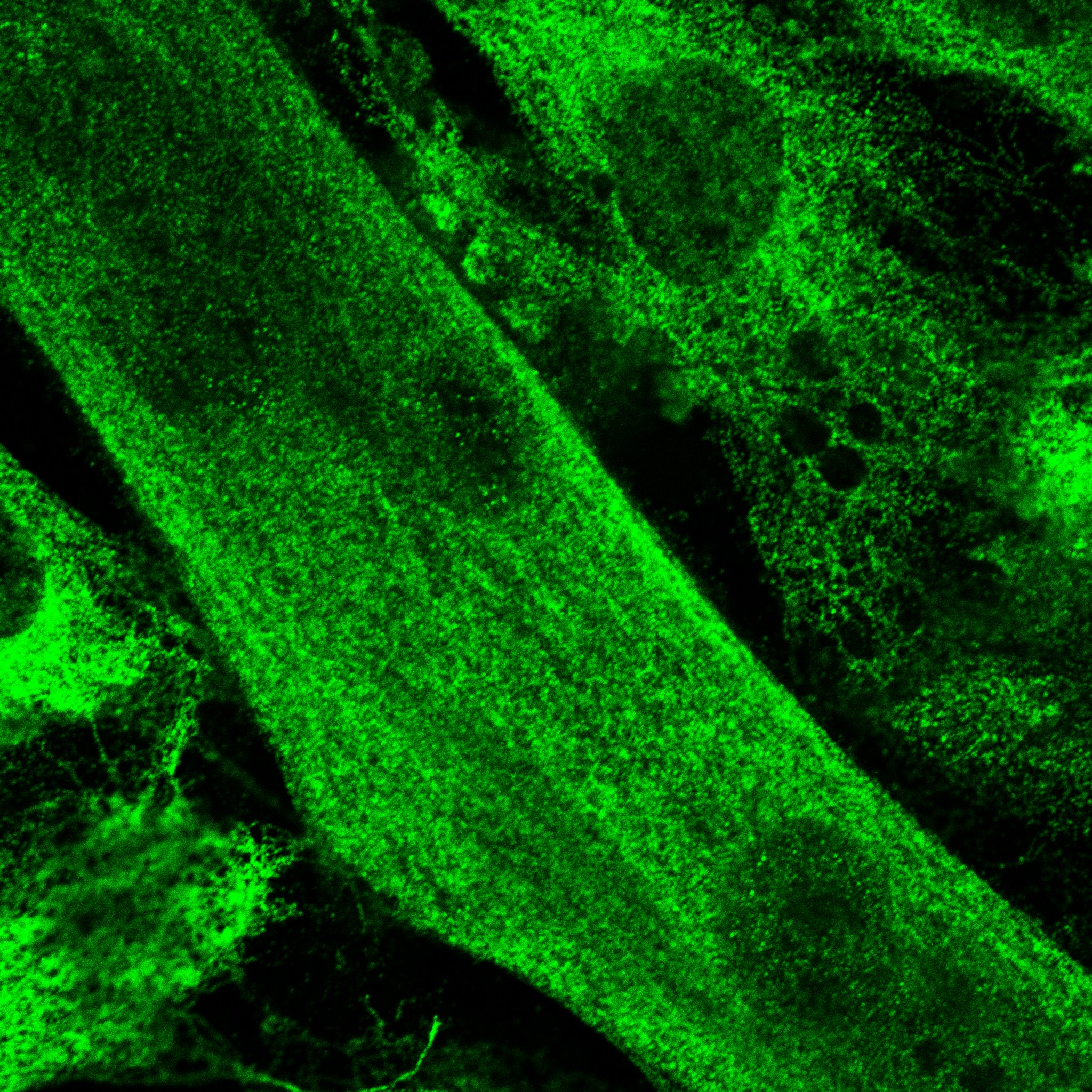

Supplement: Supplementary file 20 — Source data Fig. 6 [file 44318_2024_356_MOESM20_ESM.zip › Figure 6/Fig 6D Wild Type Reep5.jpg]

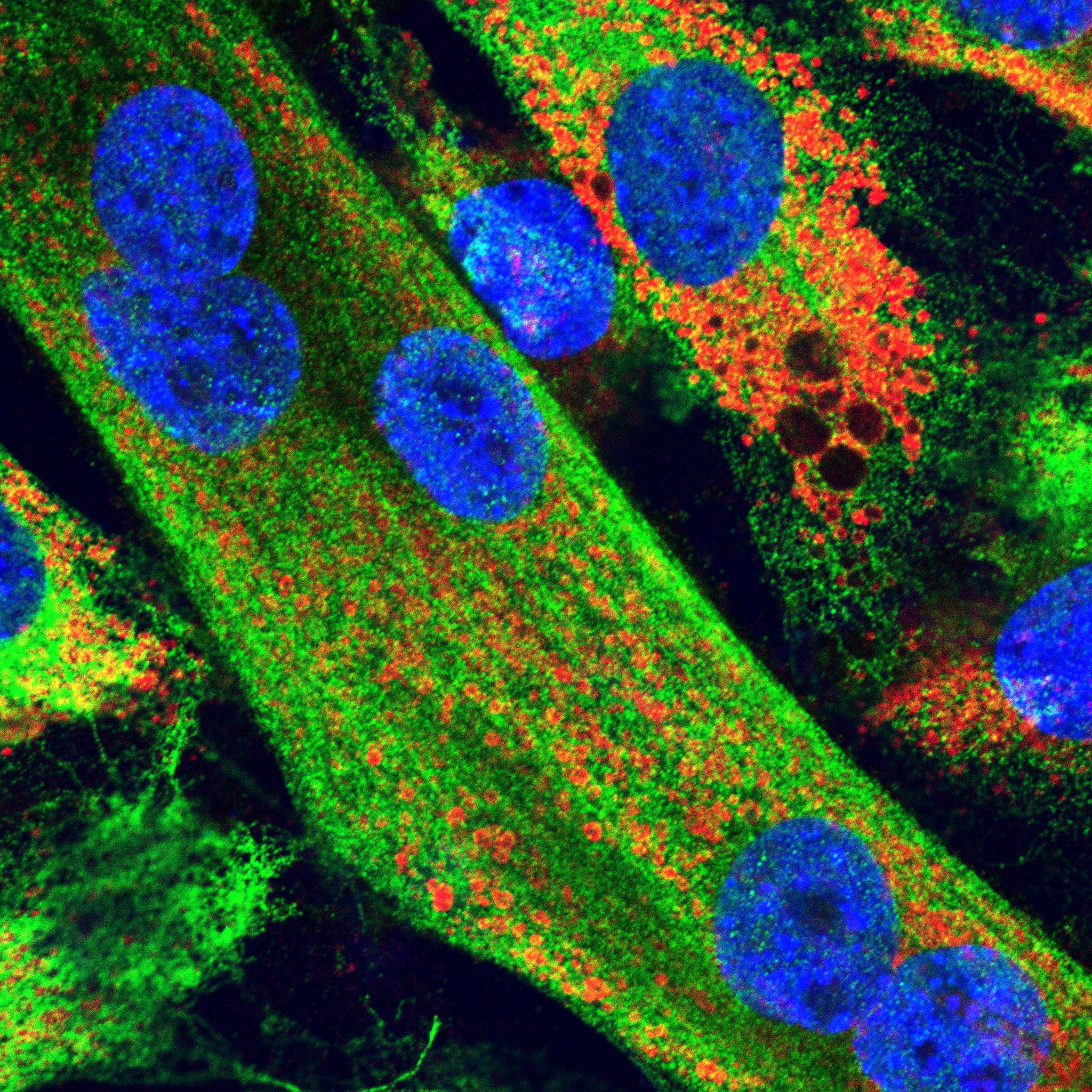

Supplement: Supplementary file 20 — Source data Fig. 6 [file 44318_2024_356_MOESM20_ESM.zip › Figure 6/Fig 6D Wild Type Merge.jpg]

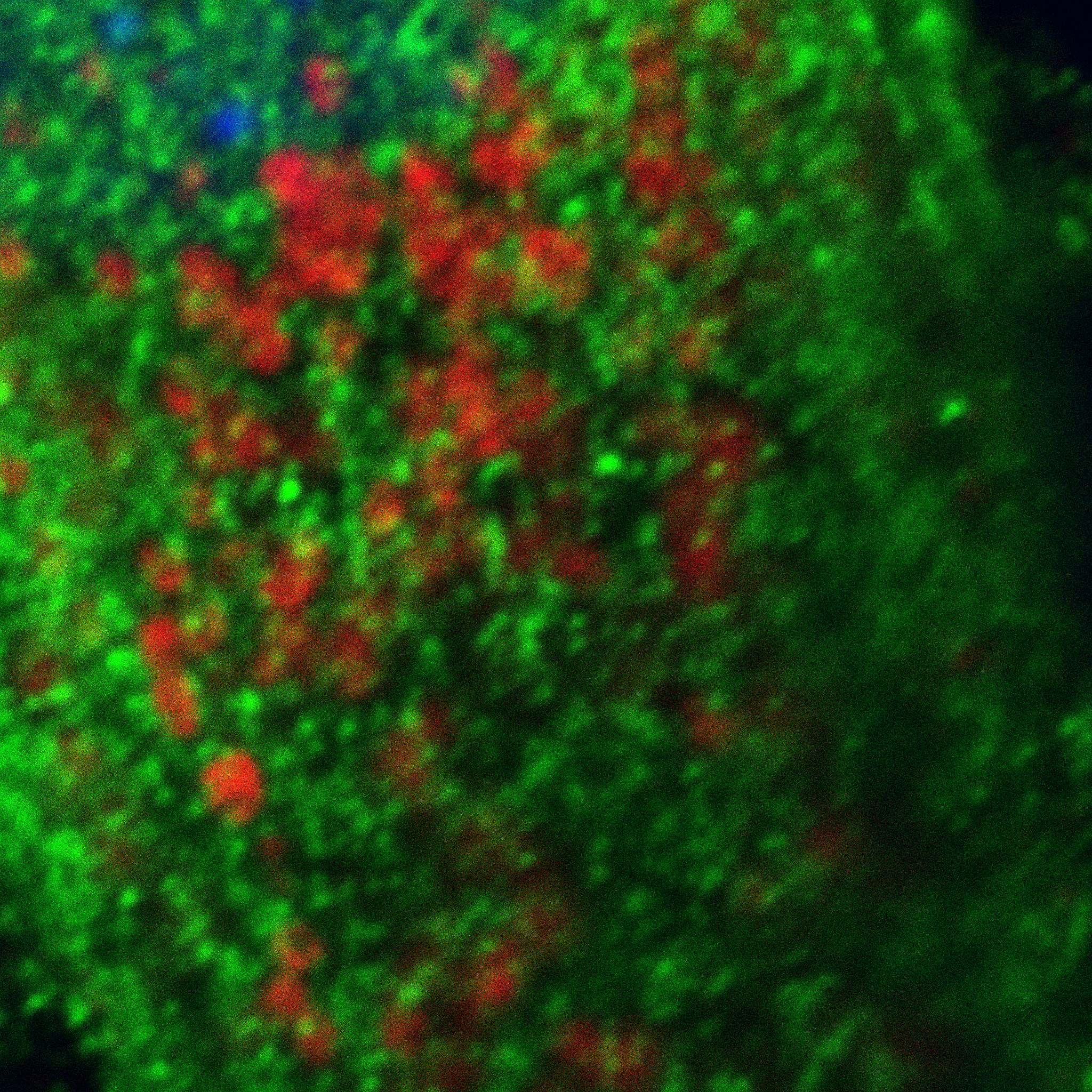

Supplement: Supplementary file 20 — Source data Fig. 6 [file 44318_2024_356_MOESM20_ESM.zip › Figure 6/Fig 6D Fam134b KO crop.tif]

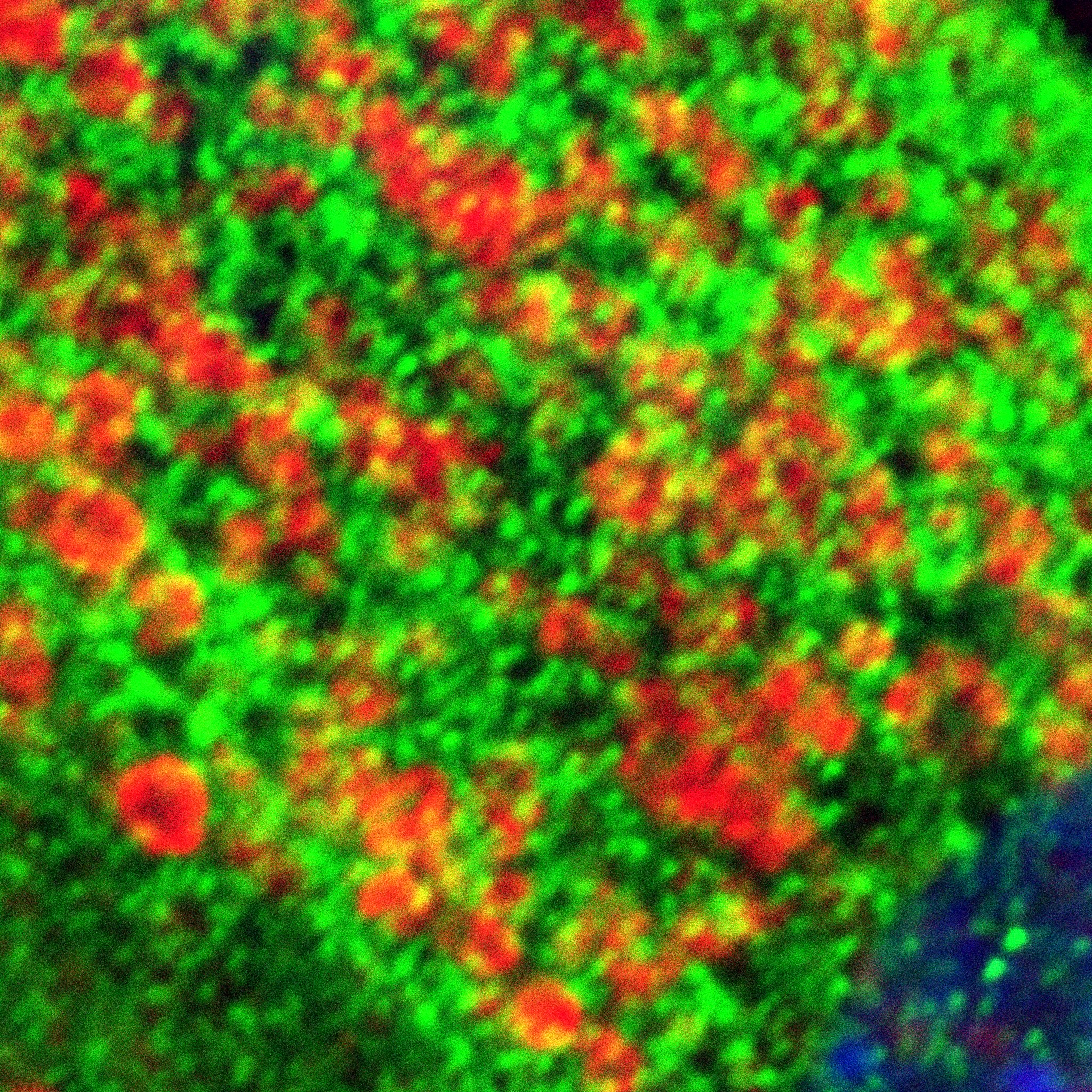

Supplement: Supplementary file 20 — Source data Fig. 6 [file 44318_2024_356_MOESM20_ESM.zip › Figure 6/Fig 6D Wild-Type crop.jpg]

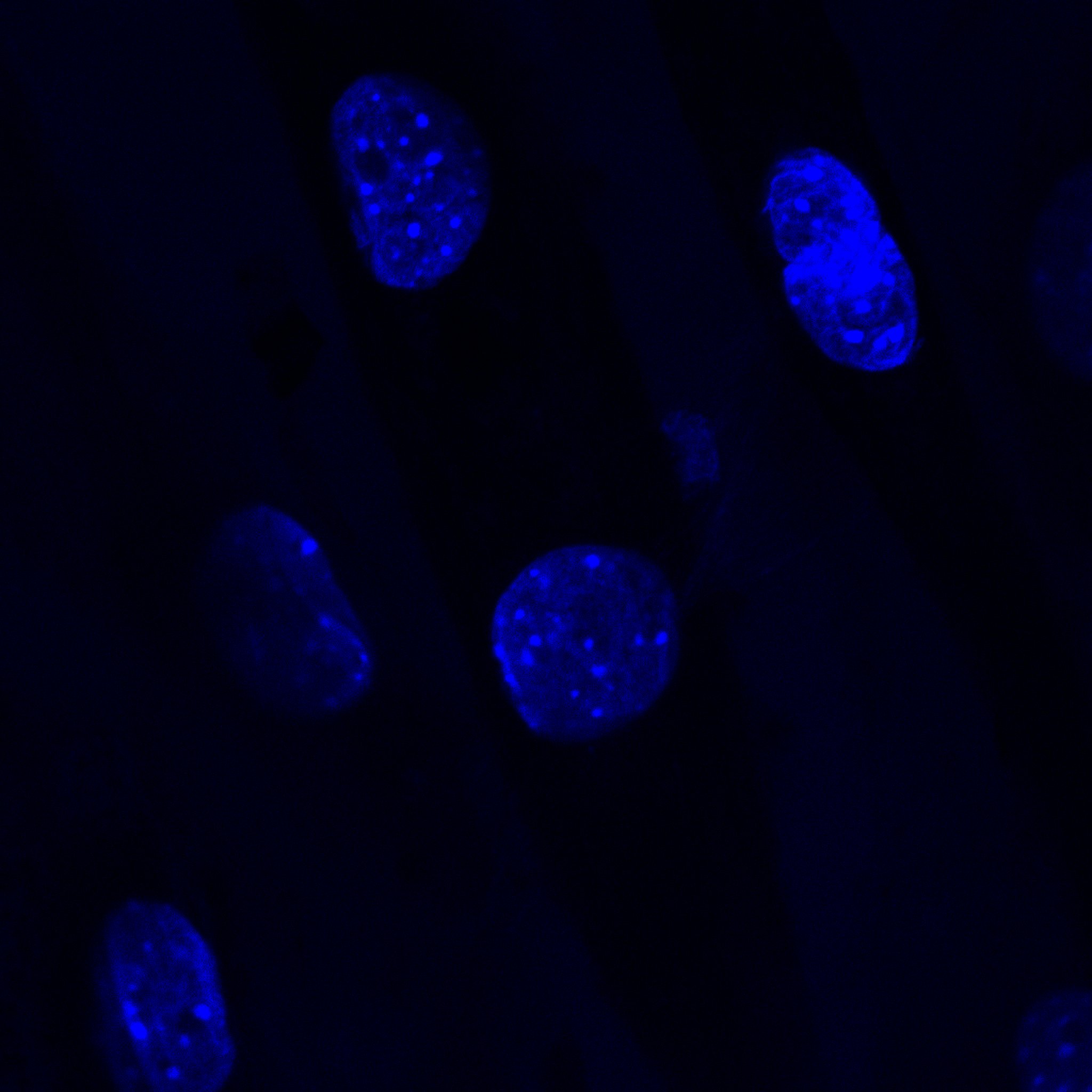

Supplement: Supplementary file 20 — Source data Fig. 6 [file 44318_2024_356_MOESM20_ESM.zip › Figure 6/Fig 6D Fam134b KO Dapi.jpg]

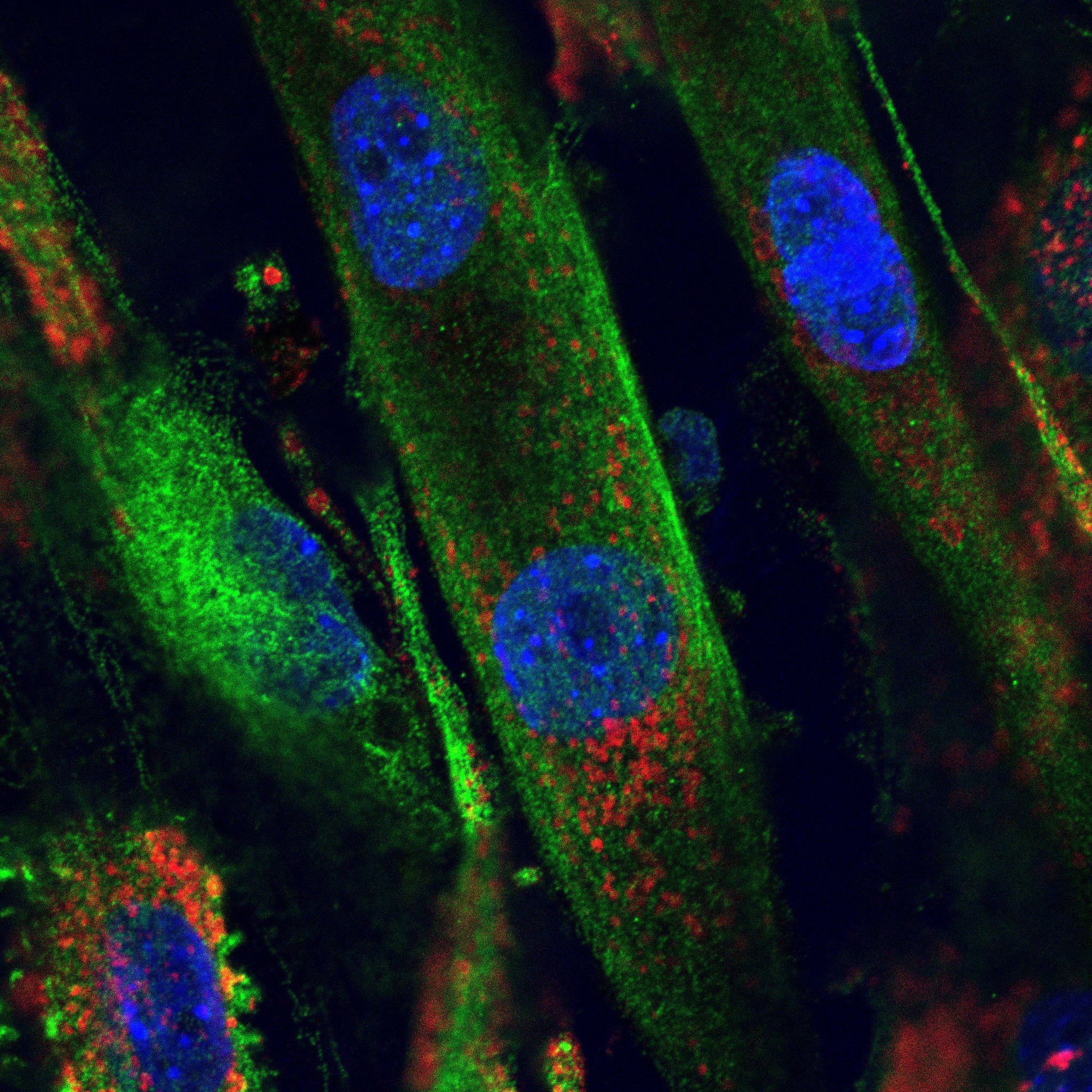

Supplement: Supplementary file 20 — Source data Fig. 6 [file 44318_2024_356_MOESM20_ESM.zip › Figure 6/Fig 6D Fam134b KO Merge.jpg]

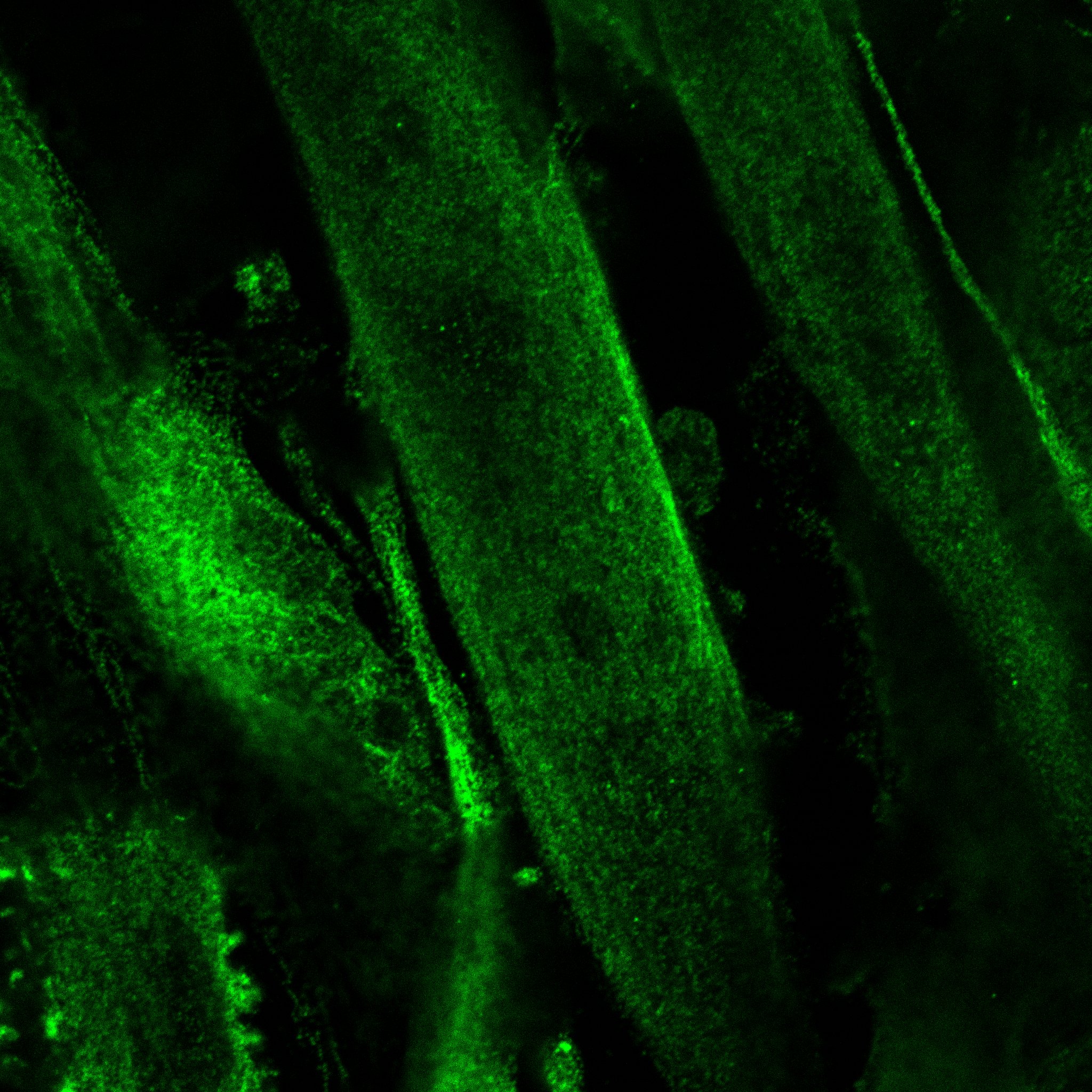

Supplement: Supplementary file 20 — Source data Fig. 6 [file 44318_2024_356_MOESM20_ESM.zip › Figure 6/Fig 6D Fam134b KO Reep5.jpg]

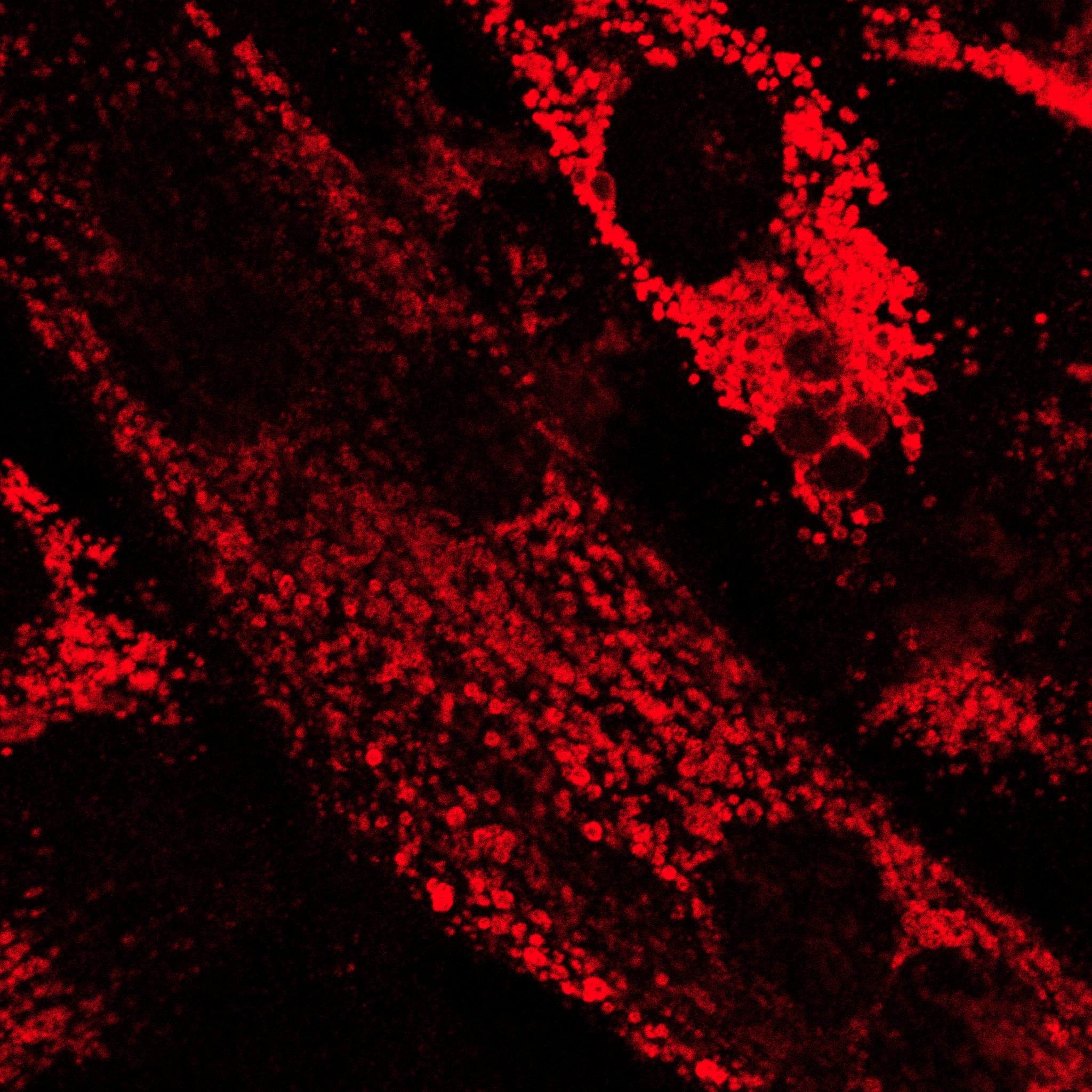

Supplement: Supplementary file 20 — Source data Fig. 6 [file 44318_2024_356_MOESM20_ESM.zip › Figure 6/Fig 6D Wild Type Lamp1.jpg]

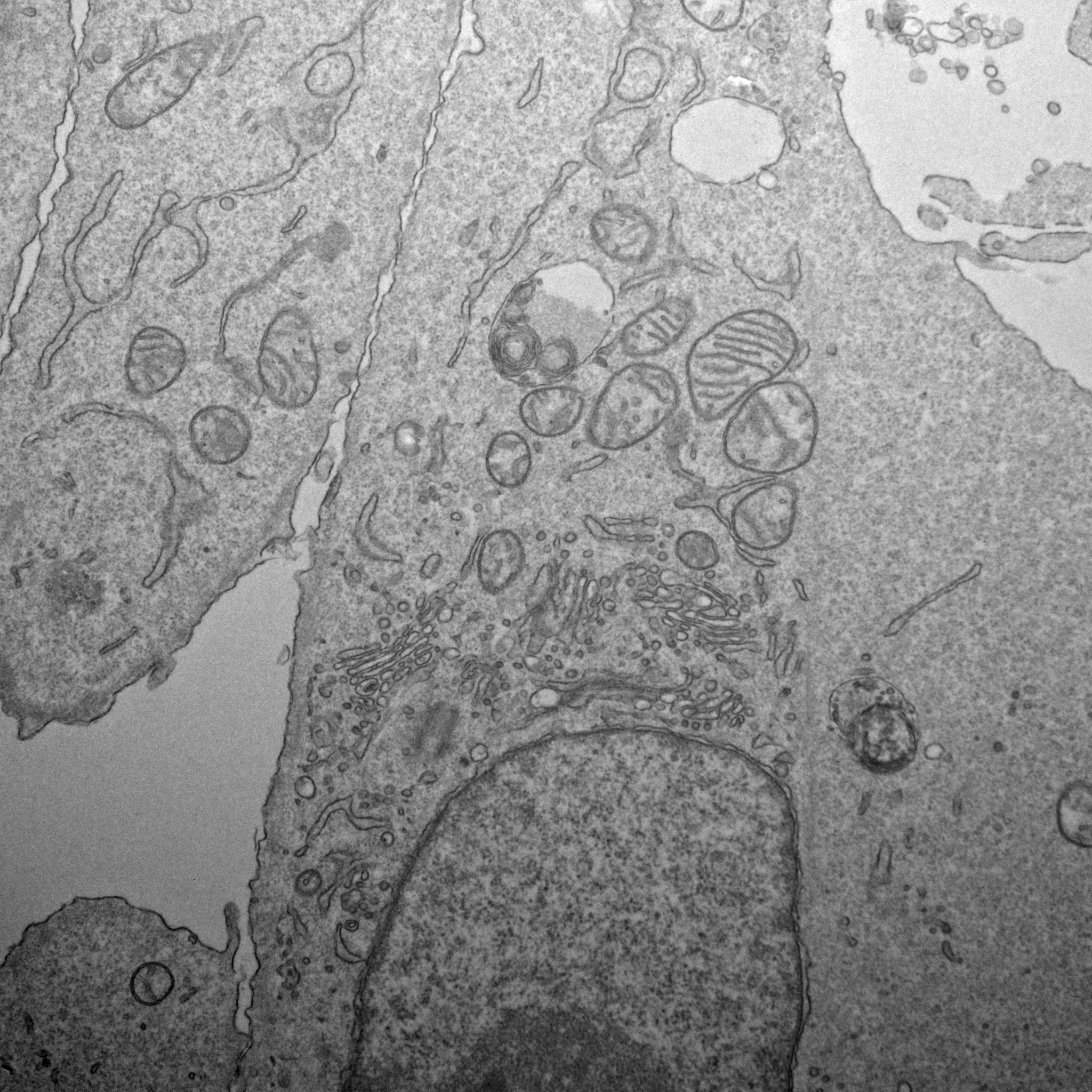

Supplement: Supplementary file 21 — Source data Fig. 7 [file 44318_2024_356_MOESM21_ESM.zip › Figure 7/Fig 7C Wild Type Myoblasts.tif]

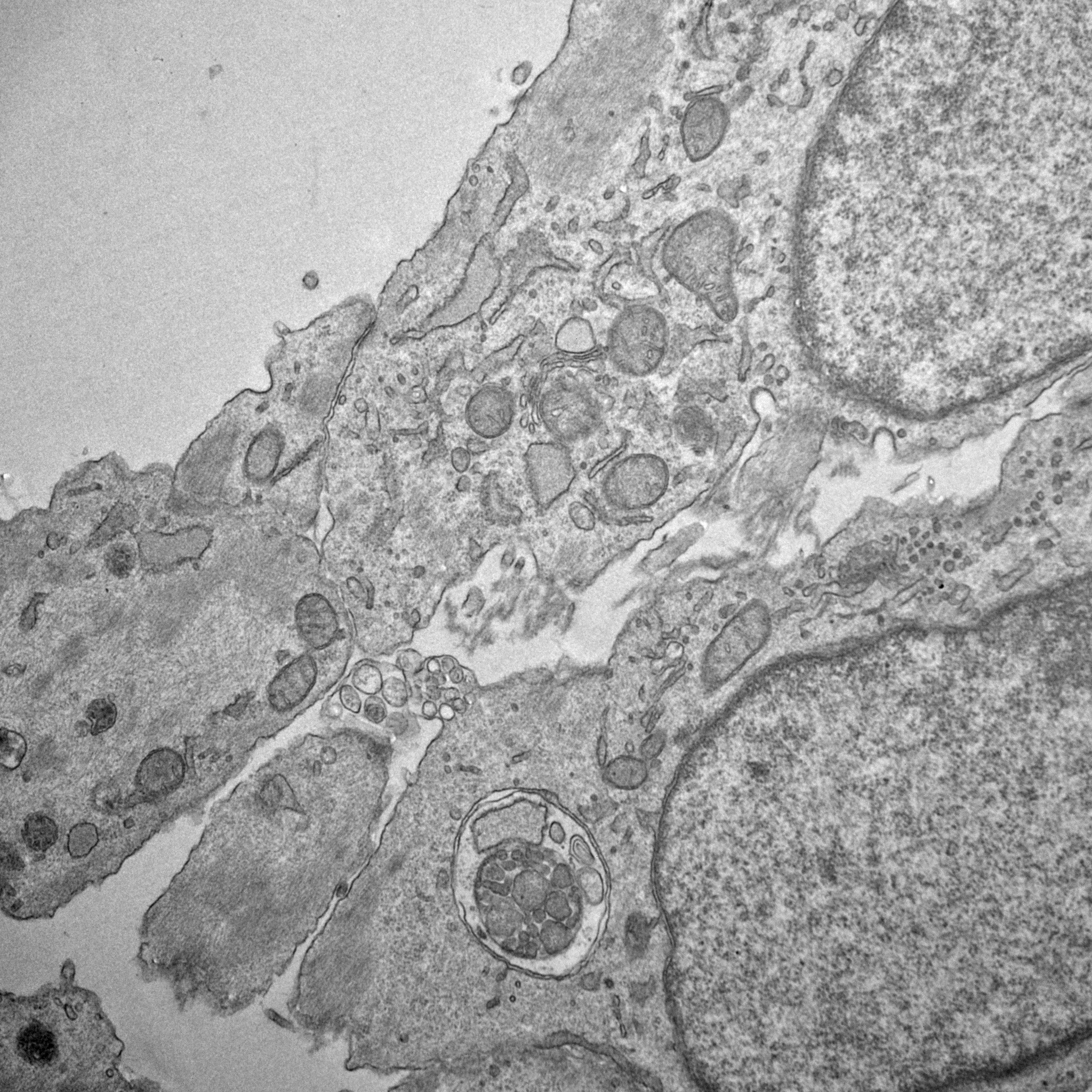

Supplement: Supplementary file 21 — Source data Fig. 7 [file 44318_2024_356_MOESM21_ESM.zip › Figure 7/Fig 7C Wild Type Myotubes 7d .tif]

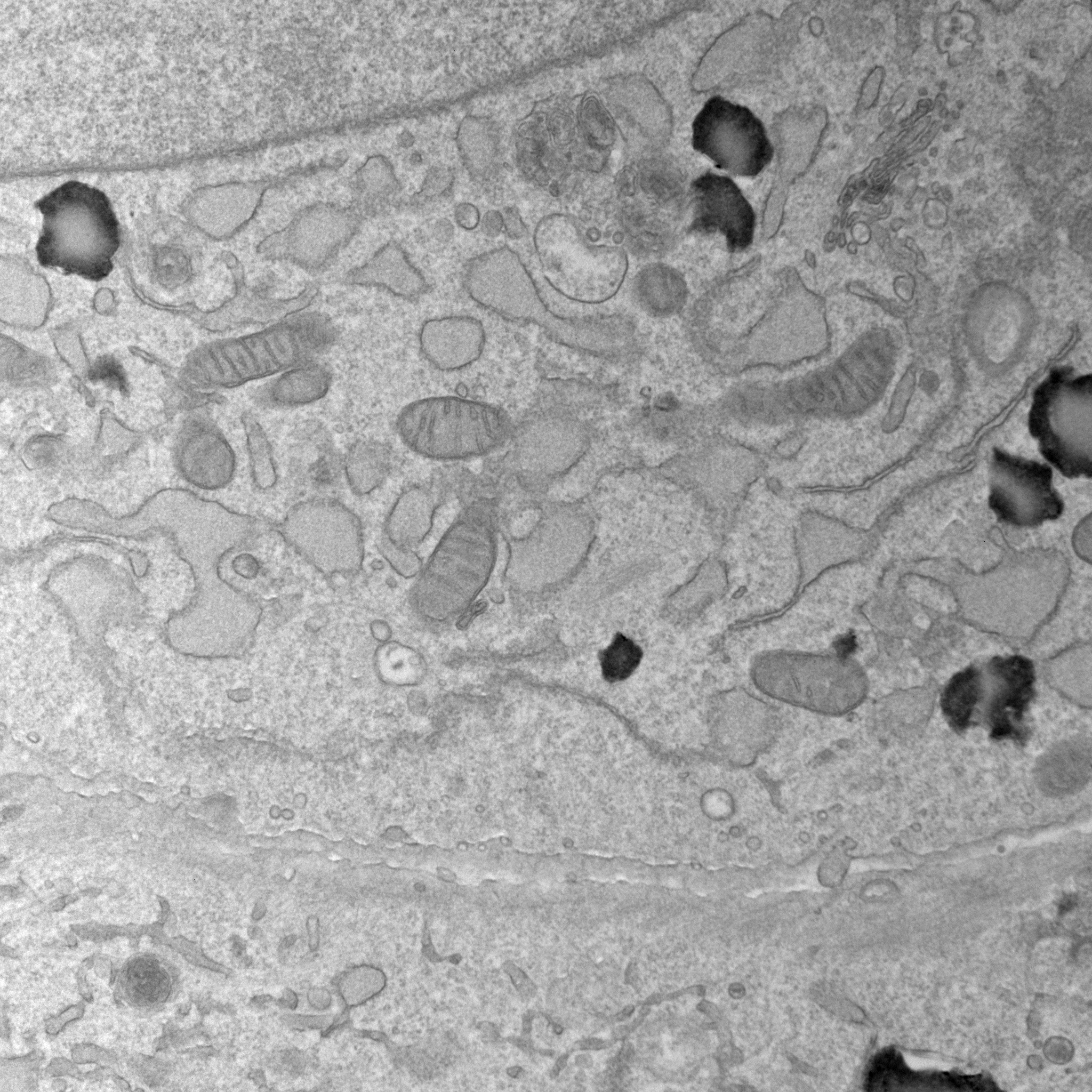

Supplement: Supplementary file 21 — Source data Fig. 7 [file 44318_2024_356_MOESM21_ESM.zip › Figure 7/Fig 7C Fam134b KO Myotubes 7d.tif]

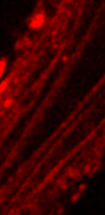

Supplement: Supplementary file 21 — Source data Fig. 7 [file 44318_2024_356_MOESM21_ESM.zip › Figure 7/Fig 7F Wild-Type myotubes Crop.tif]

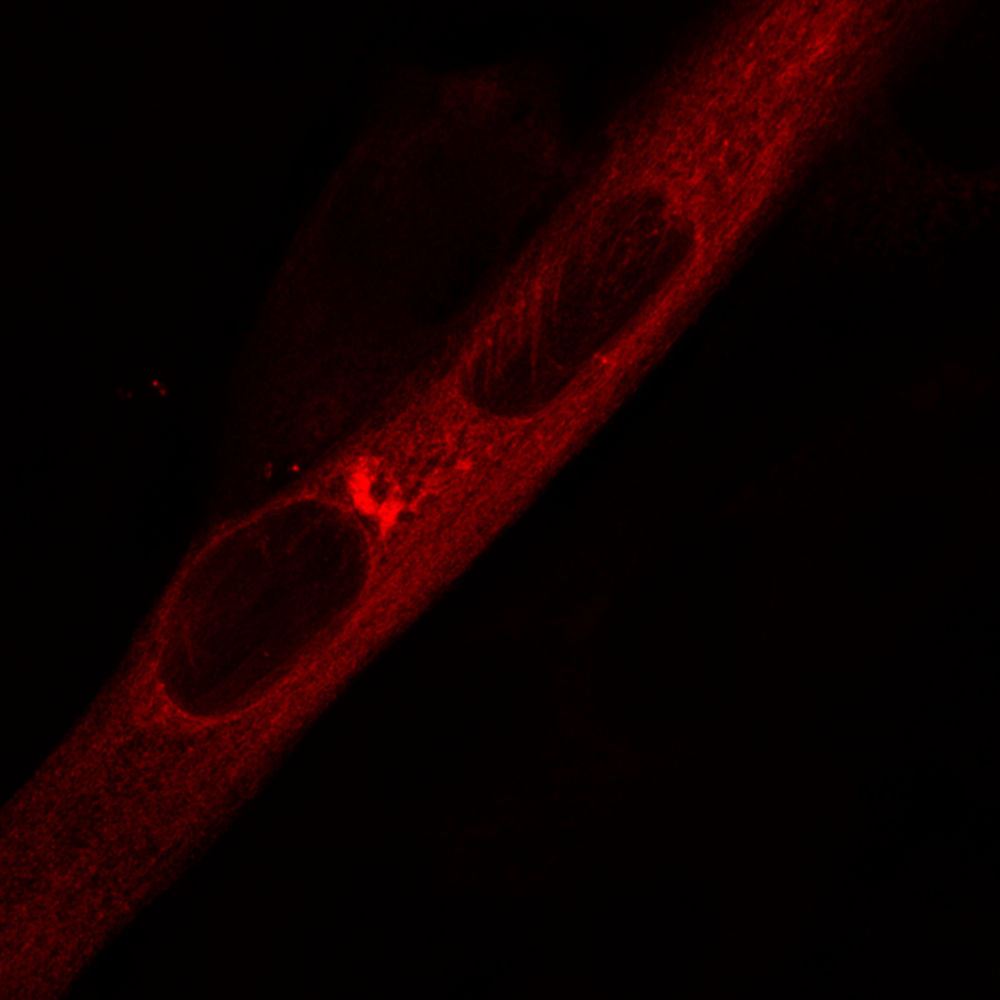

Supplement: Supplementary file 21 — Source data Fig. 7 [file 44318_2024_356_MOESM21_ESM.zip › Figure 7/Fig 7F Fam134b KO myotubes.tif]

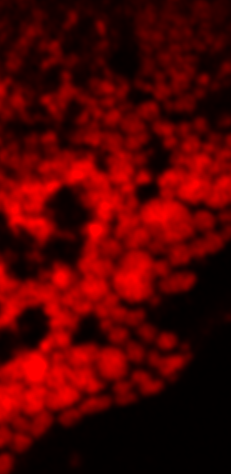

Supplement: Supplementary file 21 — Source data Fig. 7 [file 44318_2024_356_MOESM21_ESM.zip › Figure 7/Fig 7F Fam134b KO Myoblasts crop.tif]

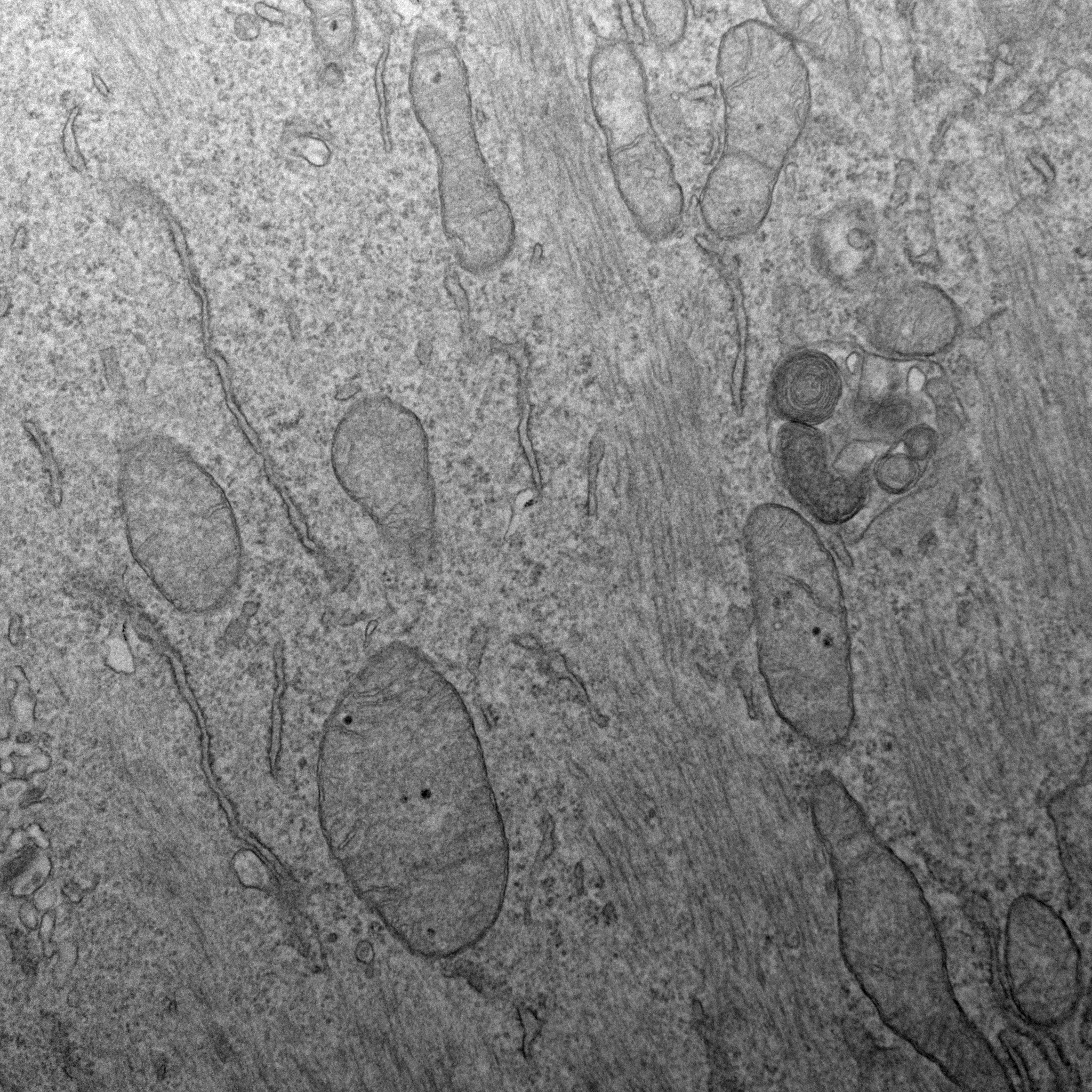

Supplement: Supplementary file 21 — Source data Fig. 7 [file 44318_2024_356_MOESM21_ESM.zip › Figure 7/Fig 7D Fam134b KO + hFAM134B2.tif]

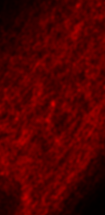

Supplement: Supplementary file 21 — Source data Fig. 7 [file 44318_2024_356_MOESM21_ESM.zip › Figure 7/Fig 7F Fam134b KO myotubes Crop.tif]

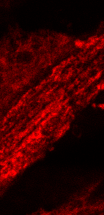

Supplement: Supplementary file 21 — Source data Fig. 7 [file 44318_2024_356_MOESM21_ESM.zip › Figure 7/Fig 7F Fam134b KO + hFAM134B2 myotubes Crop.tif]

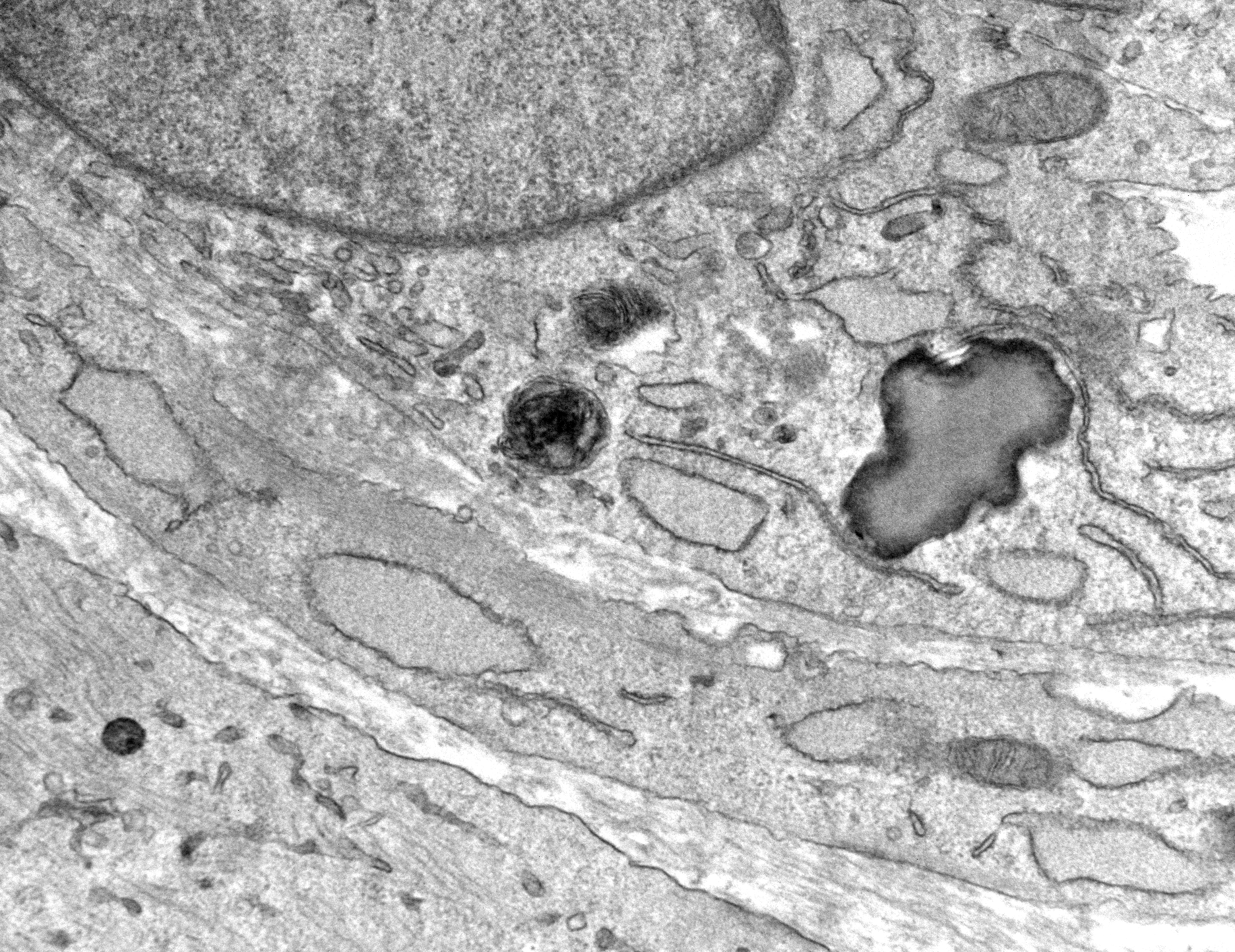

Supplement: Supplementary file 21 — Source data Fig. 7 [file 44318_2024_356_MOESM21_ESM.zip › Figure 7/Fig 7C Fam134b KO Myotubes 10d.tif]

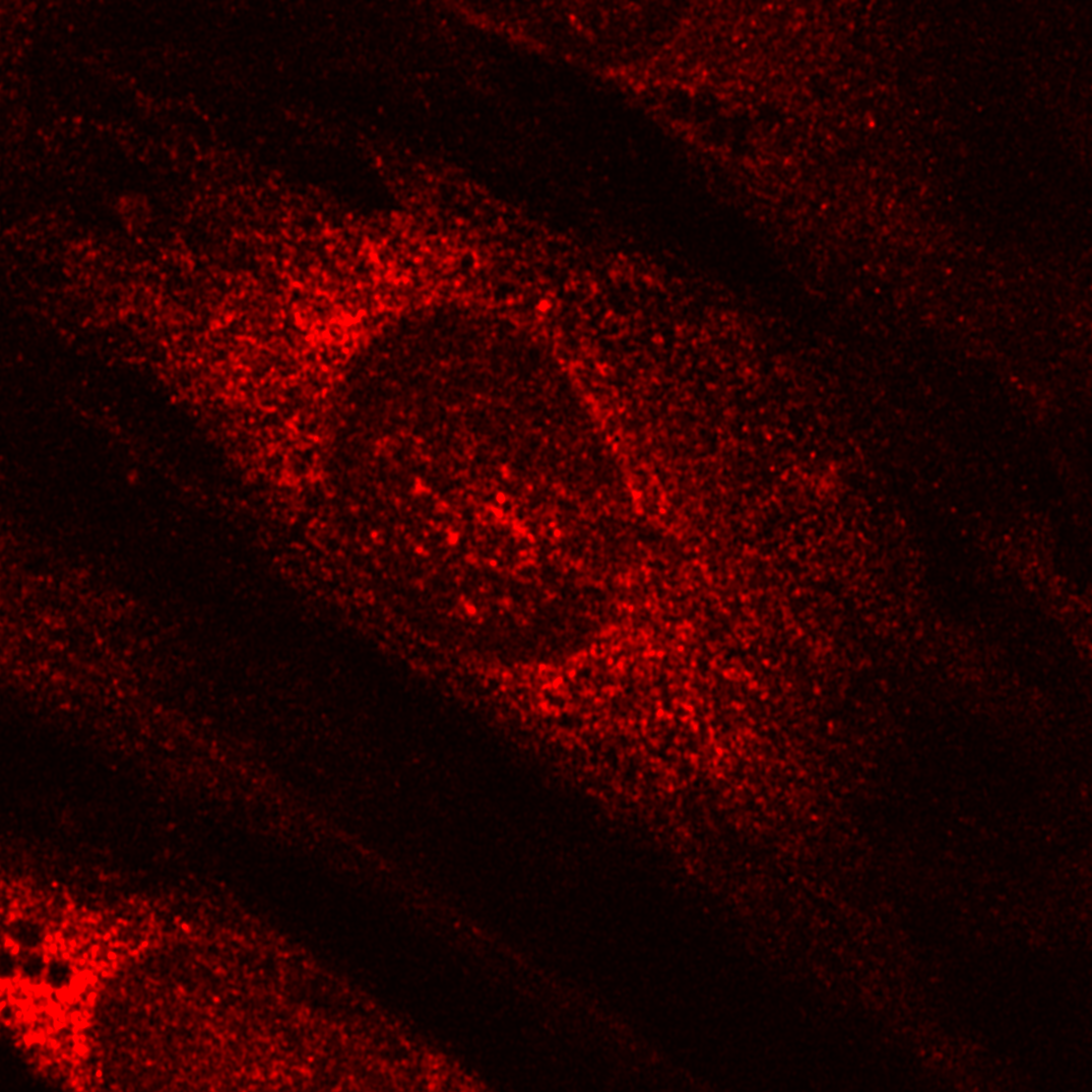

Supplement: Supplementary file 21 — Source data Fig. 7 [file 44318_2024_356_MOESM21_ESM.zip › Figure 7/Fig 7F Wild Type Myoblasts.tif]

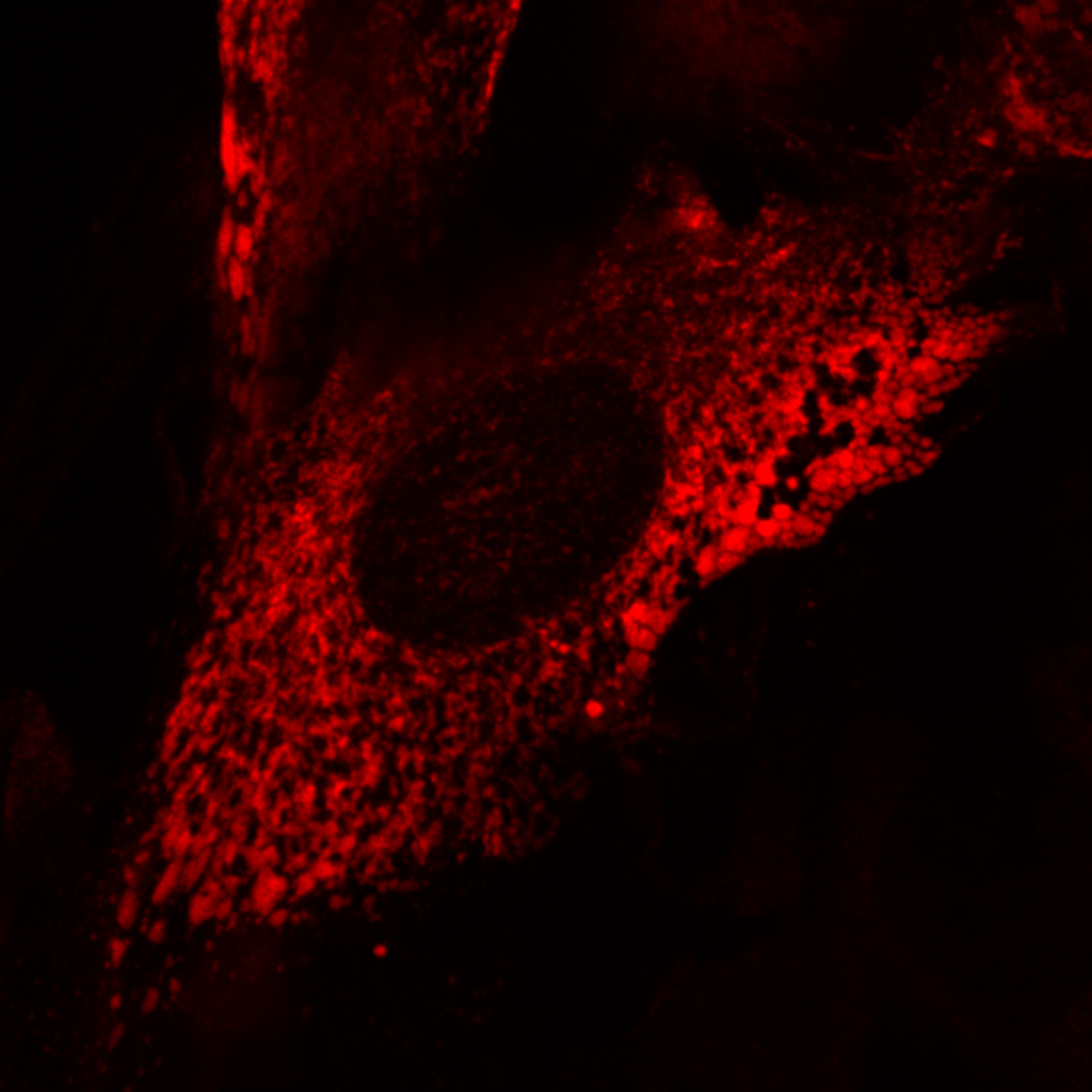

Supplement: Supplementary file 21 — Source data Fig. 7 [file 44318_2024_356_MOESM21_ESM.zip › Figure 7/Fig 7F Fam134b KO Myoblasts.tif]

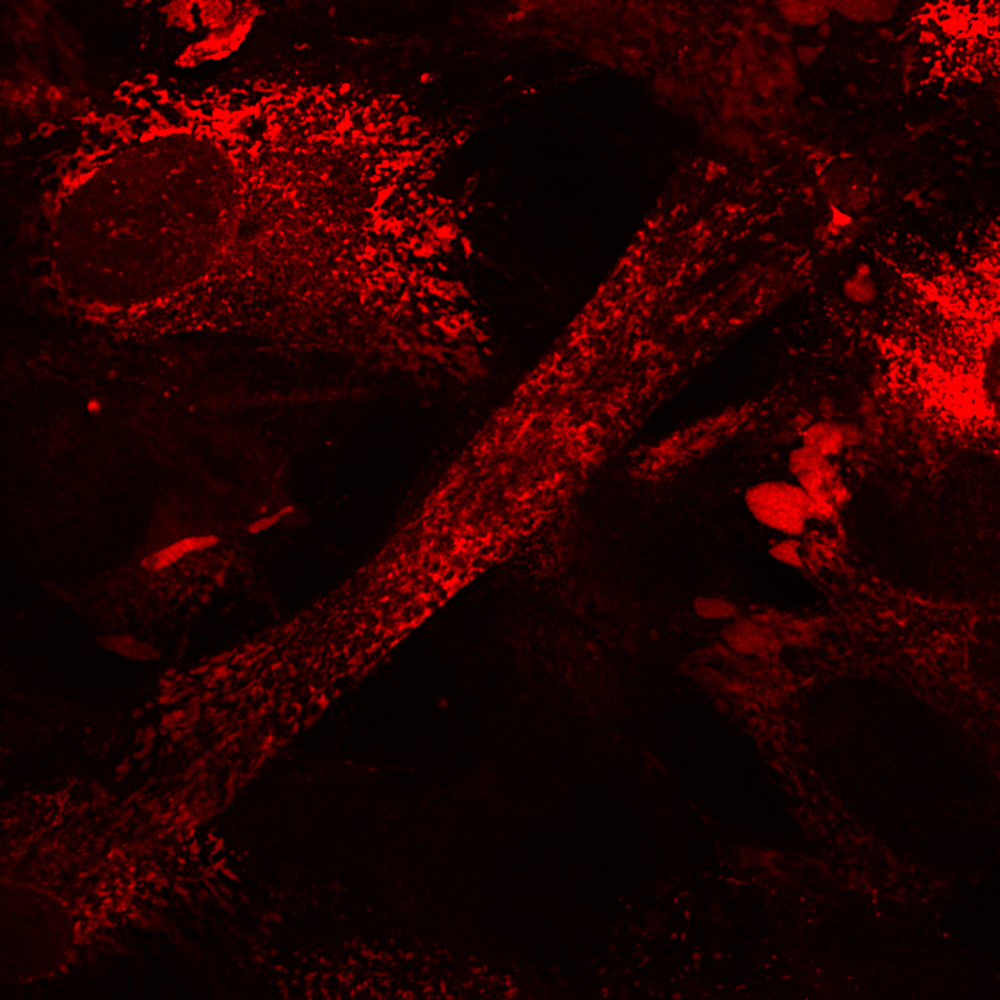

Supplement: Supplementary file 21 — Source data Fig. 7 [file 44318_2024_356_MOESM21_ESM.zip › Figure 7/Fig 7F Fam134b KO + hFAM134B2 LIR myotubes.tif]

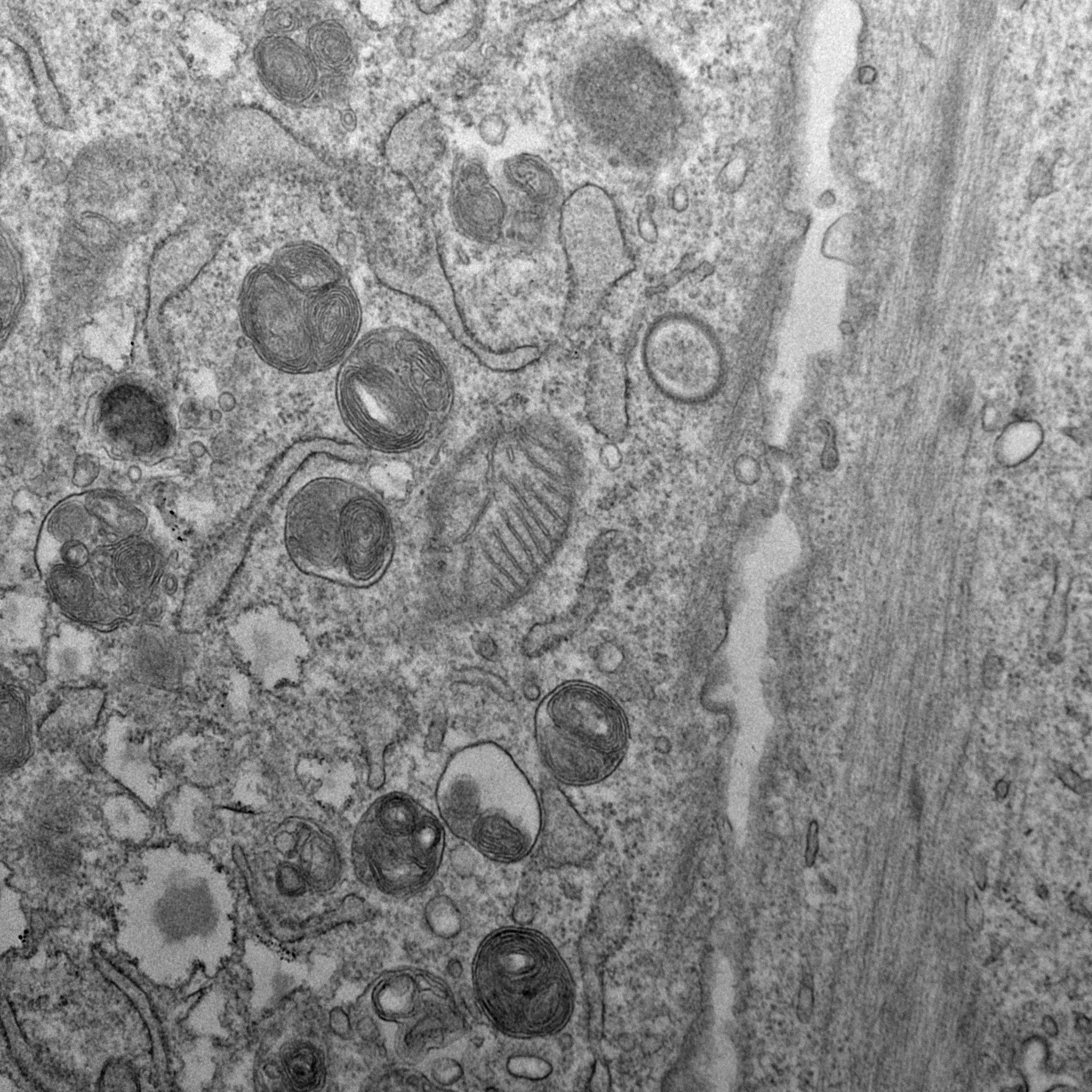

Supplement: Supplementary file 21 — Source data Fig. 7 [file 44318_2024_356_MOESM21_ESM.zip › Figure 7/Fig 7D Fam134b KO + hFAM134b LIR.tif]

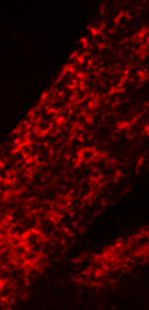

Supplement: Supplementary file 21 — Source data Fig. 7 [file 44318_2024_356_MOESM21_ESM.zip › Figure 7/Fig 7F Fam134b KO + hFAM134B2 LIR myotubes Crop.tif]

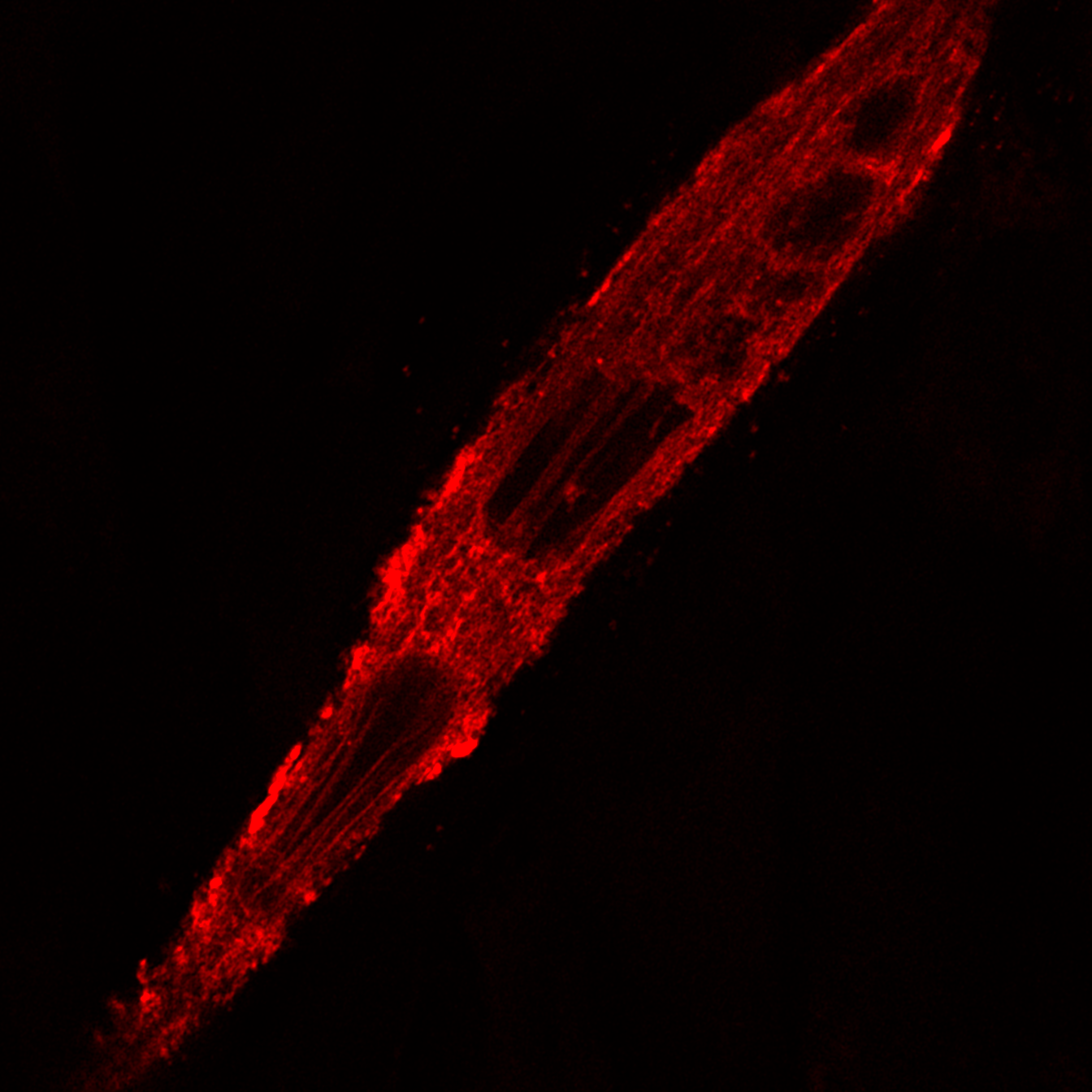

Supplement: Supplementary file 21 — Source data Fig. 7 [file 44318_2024_356_MOESM21_ESM.zip › Figure 7/Fig 7F Wild-Type myotubes.tif]

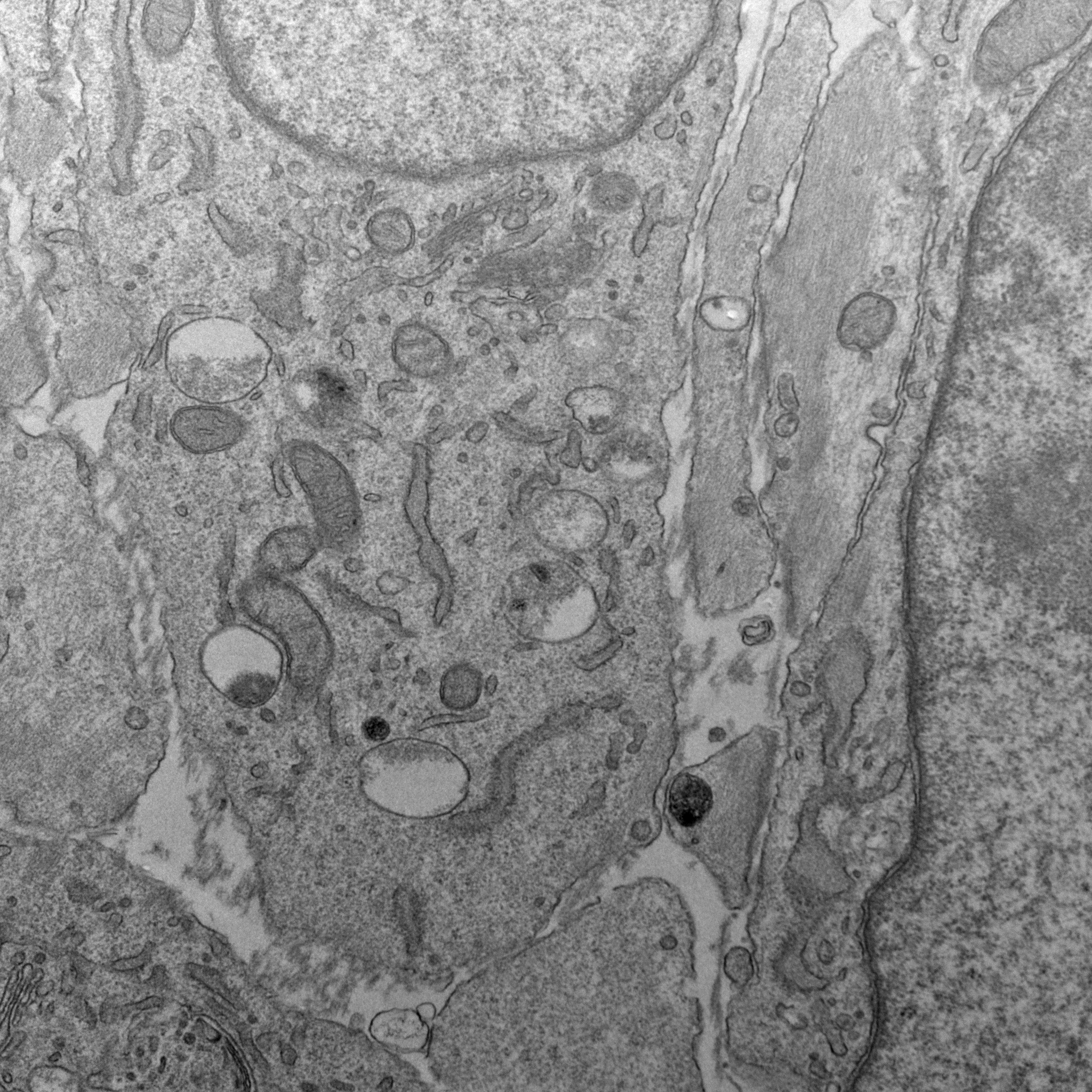

Supplement: Supplementary file 21 — Source data Fig. 7 [file 44318_2024_356_MOESM21_ESM.zip › Figure 7/Fig 7C Wild Type Myotubes 10d .tif]

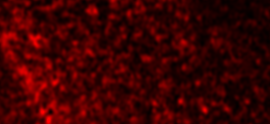

Supplement: Supplementary file 21 — Source data Fig. 7 [file 44318_2024_356_MOESM21_ESM.zip › Figure 7/Fig 7F Wild Type Myoblasts crop.tif]

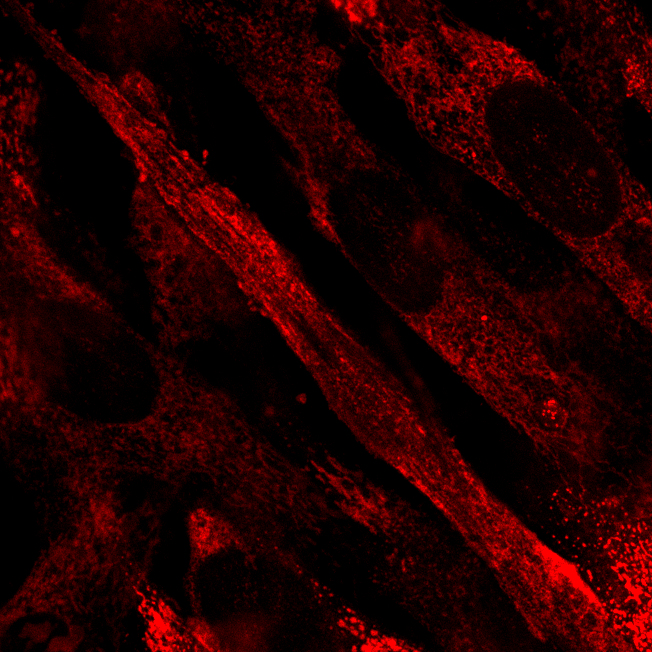

Supplement: Supplementary file 21 — Source data Fig. 7 [file 44318_2024_356_MOESM21_ESM.zip › Figure 7/Fig 7F Fam134b KO + hFAM134B2 myotubes .tif]

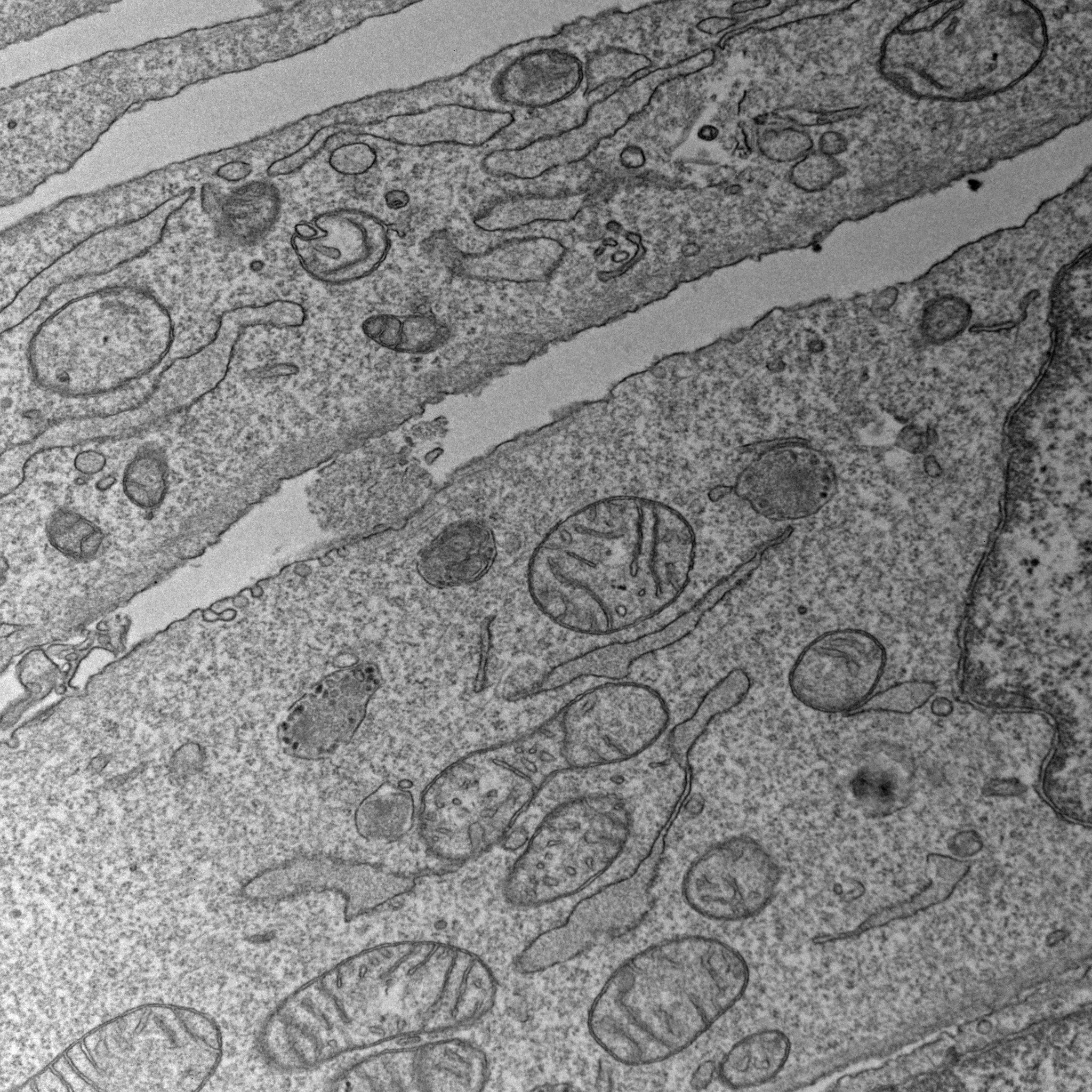

Supplement: Supplementary file 21 — Source data Fig. 7 [file 44318_2024_356_MOESM21_ESM.zip › Figure 7/Fig 7C Fam134b KO Myoblasts.tif]

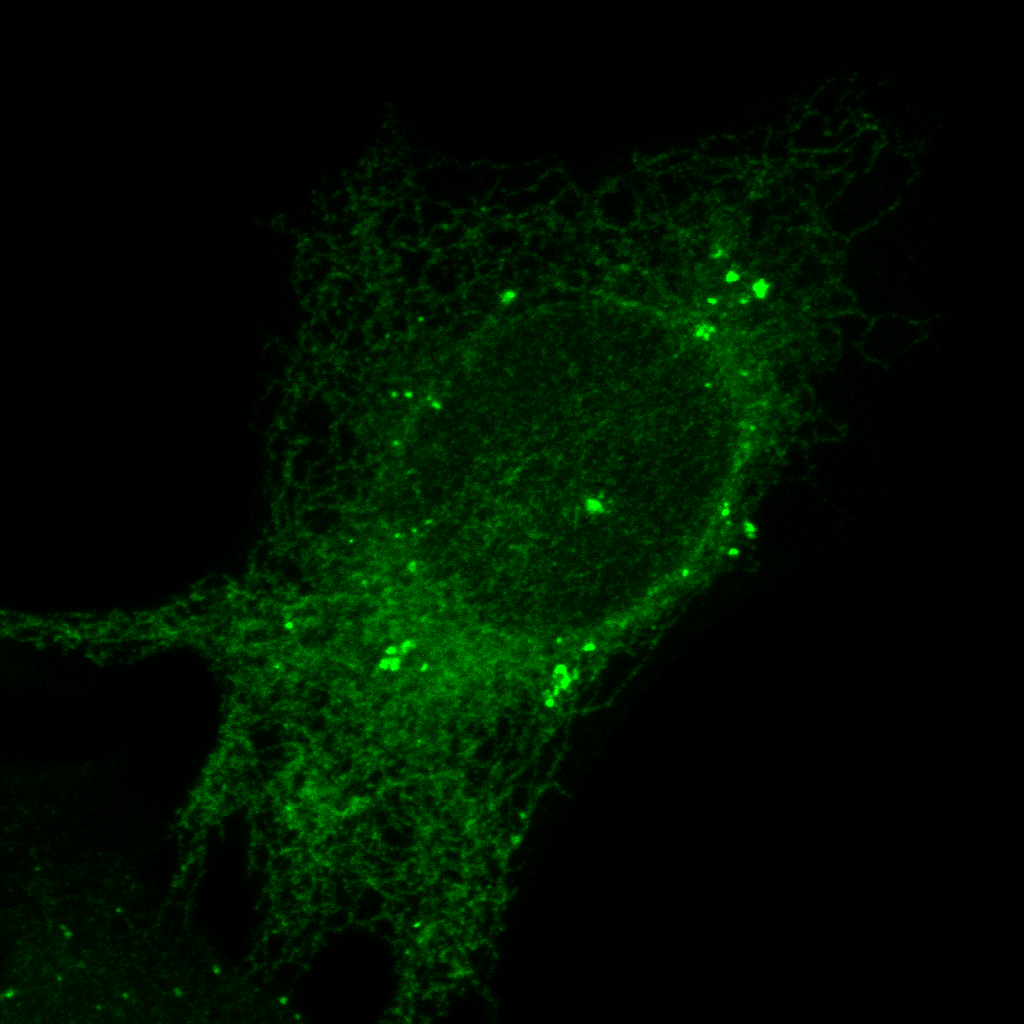

Supplement: Supplementary file 22 — EV Figure Source Data [file 44318_2024_356_MOESM22_ESM.zip › Figure EV/Appendix/Appendix 1B_HA_Fam134b2 (TOM20).tif]

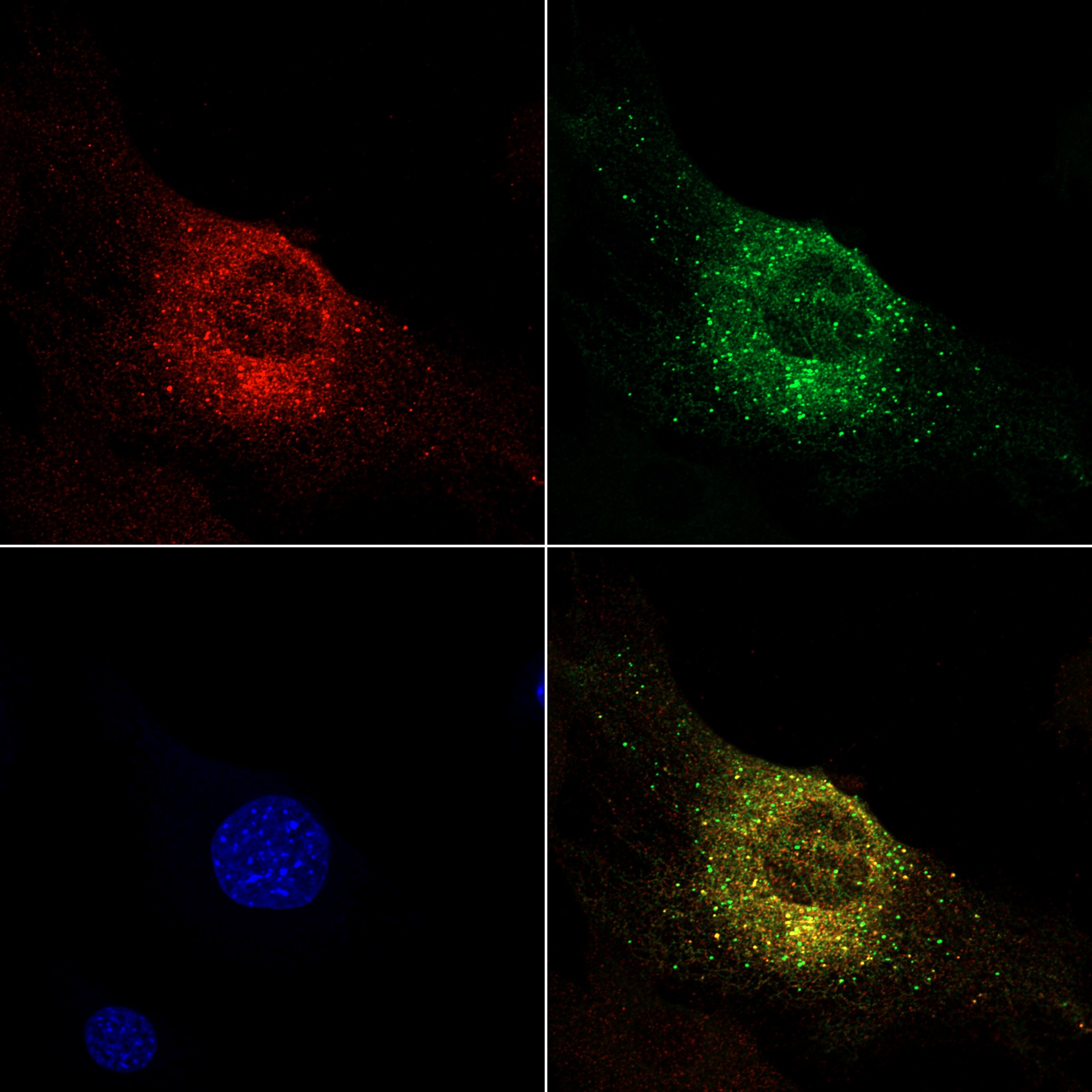

Supplement: Supplementary file 22 — EV Figure Source Data [file 44318_2024_356_MOESM22_ESM.zip › Figure EV/Appendix/Appendix 1C_Famb1 digi+triton n1.tif]

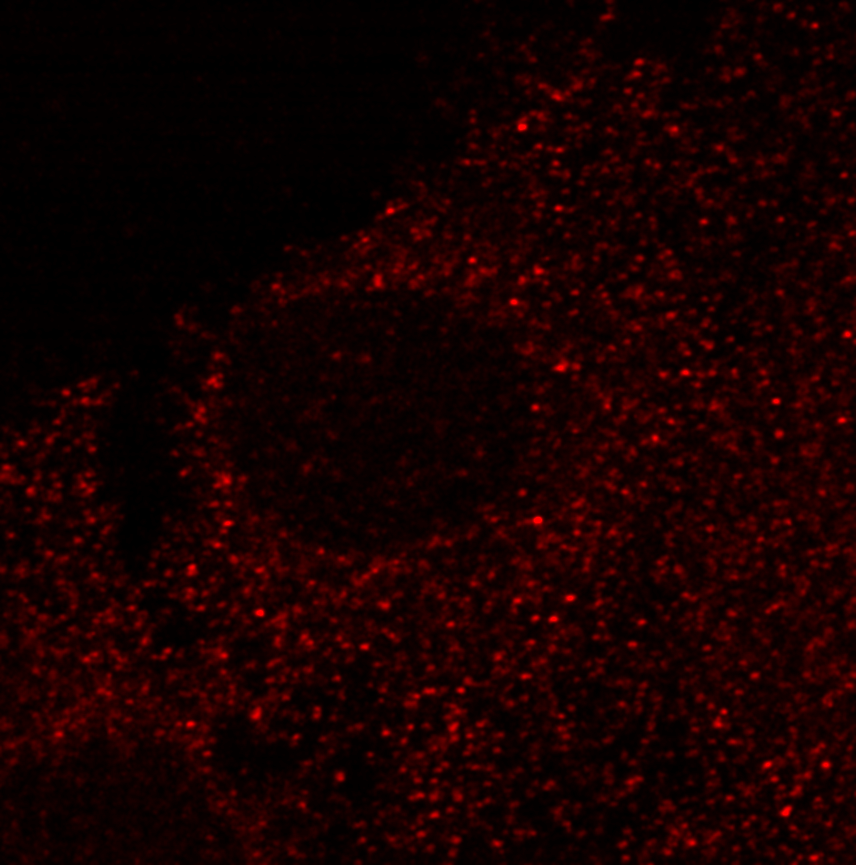

Supplement: Supplementary file 22 — EV Figure Source Data [file 44318_2024_356_MOESM22_ESM.zip › Figure EV/Appendix/Appendix 1C TGN38 Digitonin.tif]

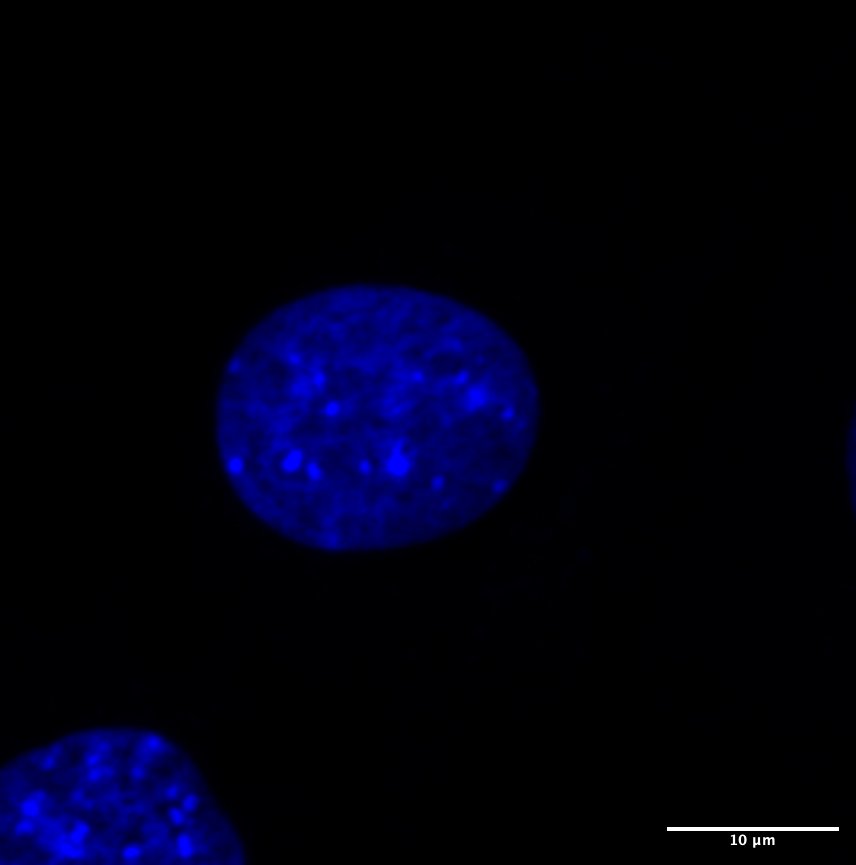

Supplement: Supplementary file 22 — EV Figure Source Data [file 44318_2024_356_MOESM22_ESM.zip › Figure EV/Appendix/Appendix 1C DAPI.jpg]

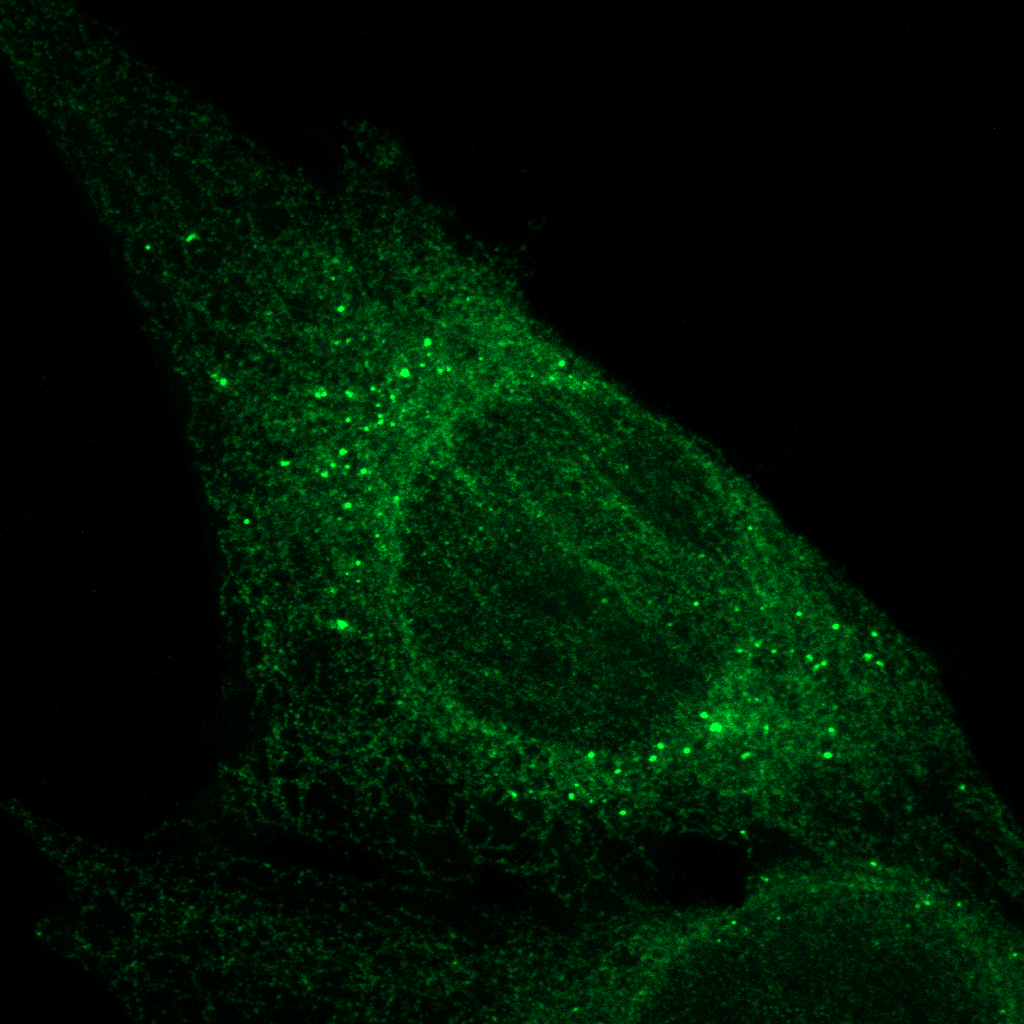

Supplement: Supplementary file 22 — EV Figure Source Data [file 44318_2024_356_MOESM22_ESM.zip › Figure EV/Appendix/Appendix 1B_HA_ Fam134b1 (TOM20).tif]

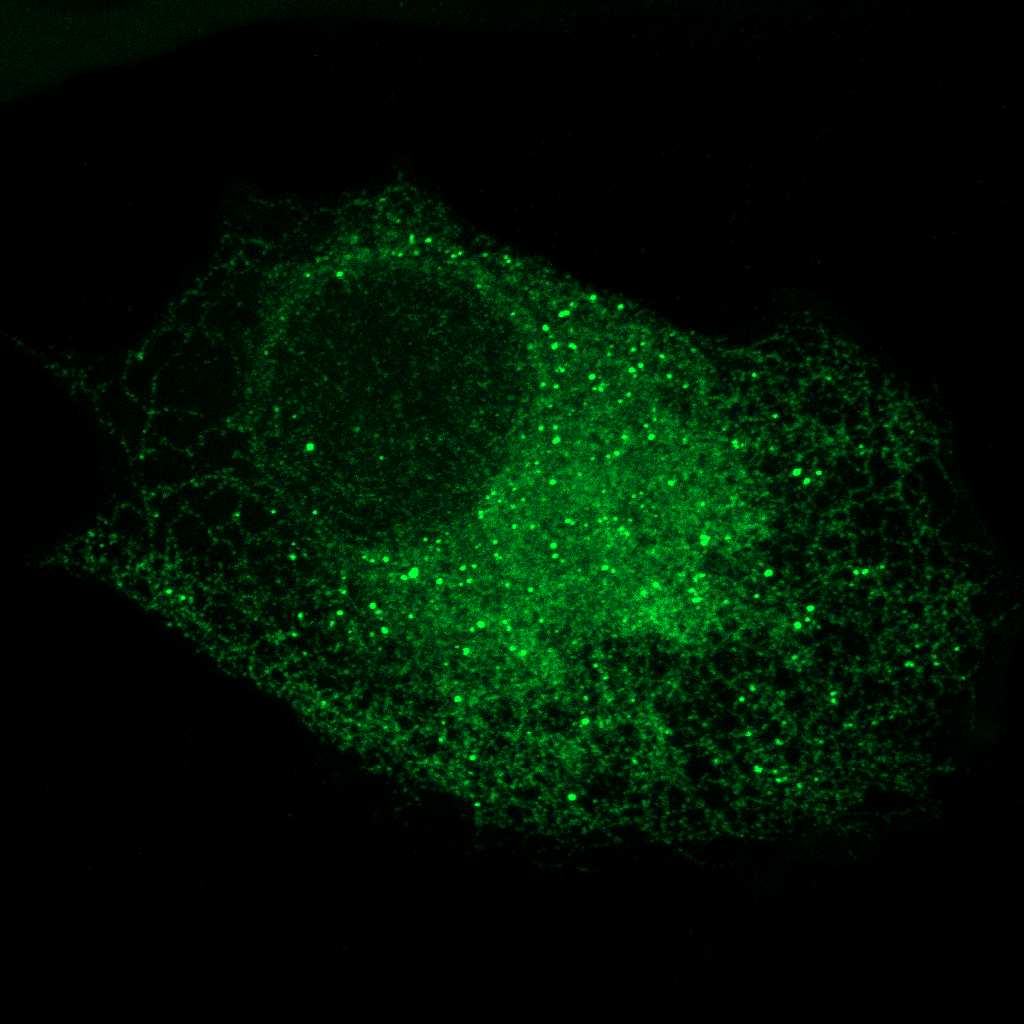

Supplement: Supplementary file 22 — EV Figure Source Data [file 44318_2024_356_MOESM22_ESM.zip › Figure EV/Appendix/Appendix 1B_HA_Fam134b2 (GM130).tif]

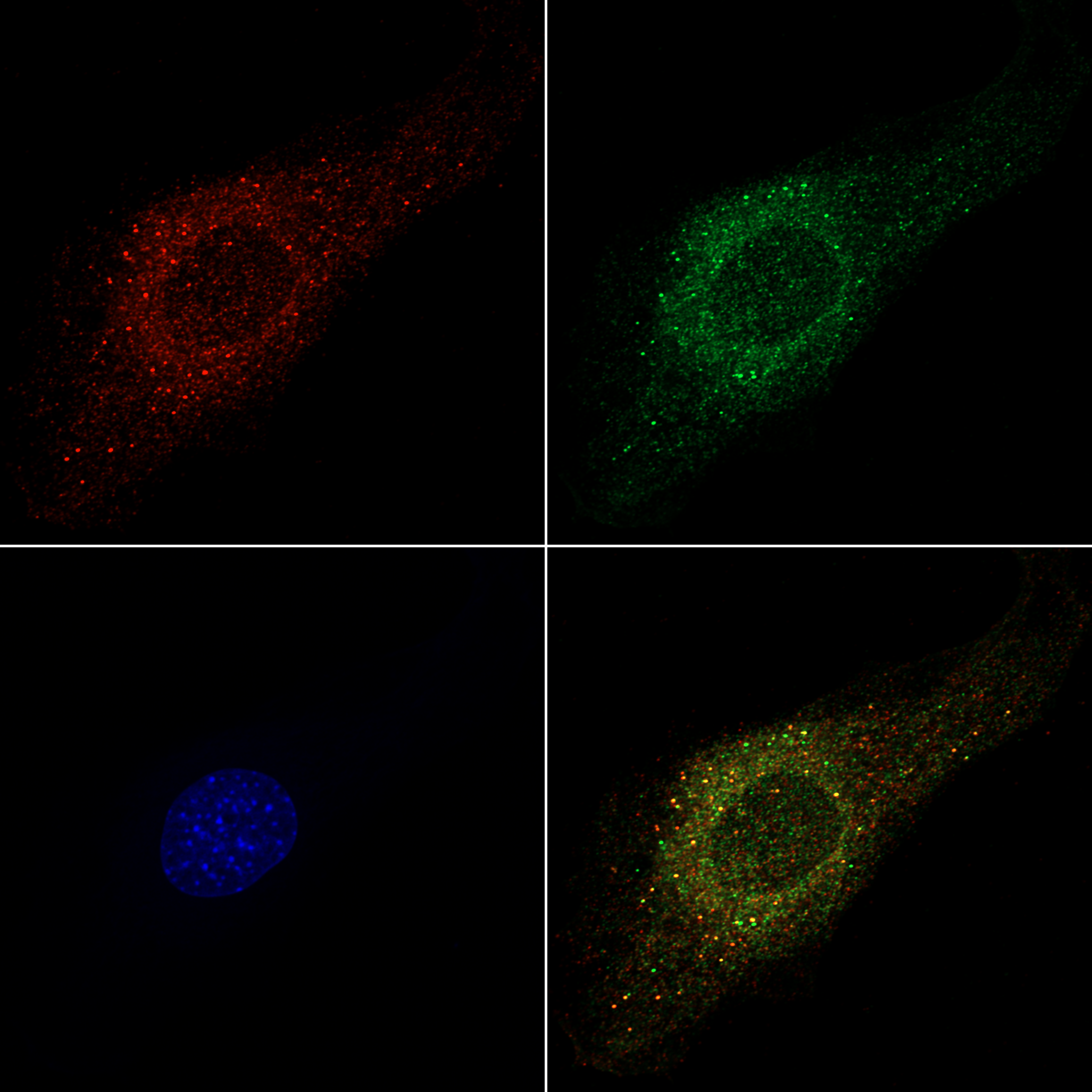

Supplement: Supplementary file 22 — EV Figure Source Data [file 44318_2024_356_MOESM22_ESM.zip › Figure EV/Appendix/Appendix 1C_Famb2 digi+triton n1.tif]

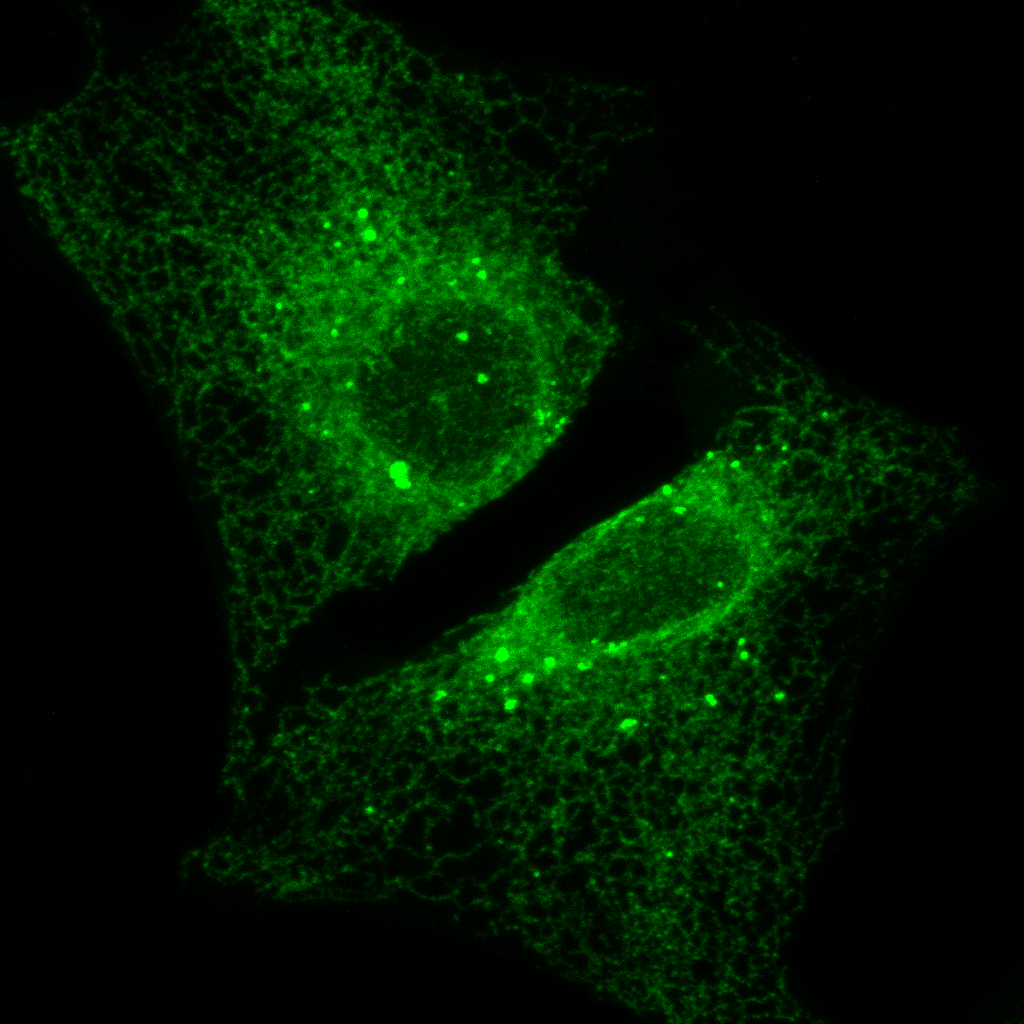

Supplement: Supplementary file 22 — EV Figure Source Data [file 44318_2024_356_MOESM22_ESM.zip › Figure EV/Appendix/Appendix 1C_HA_Fam134b2 (GM130).tif]

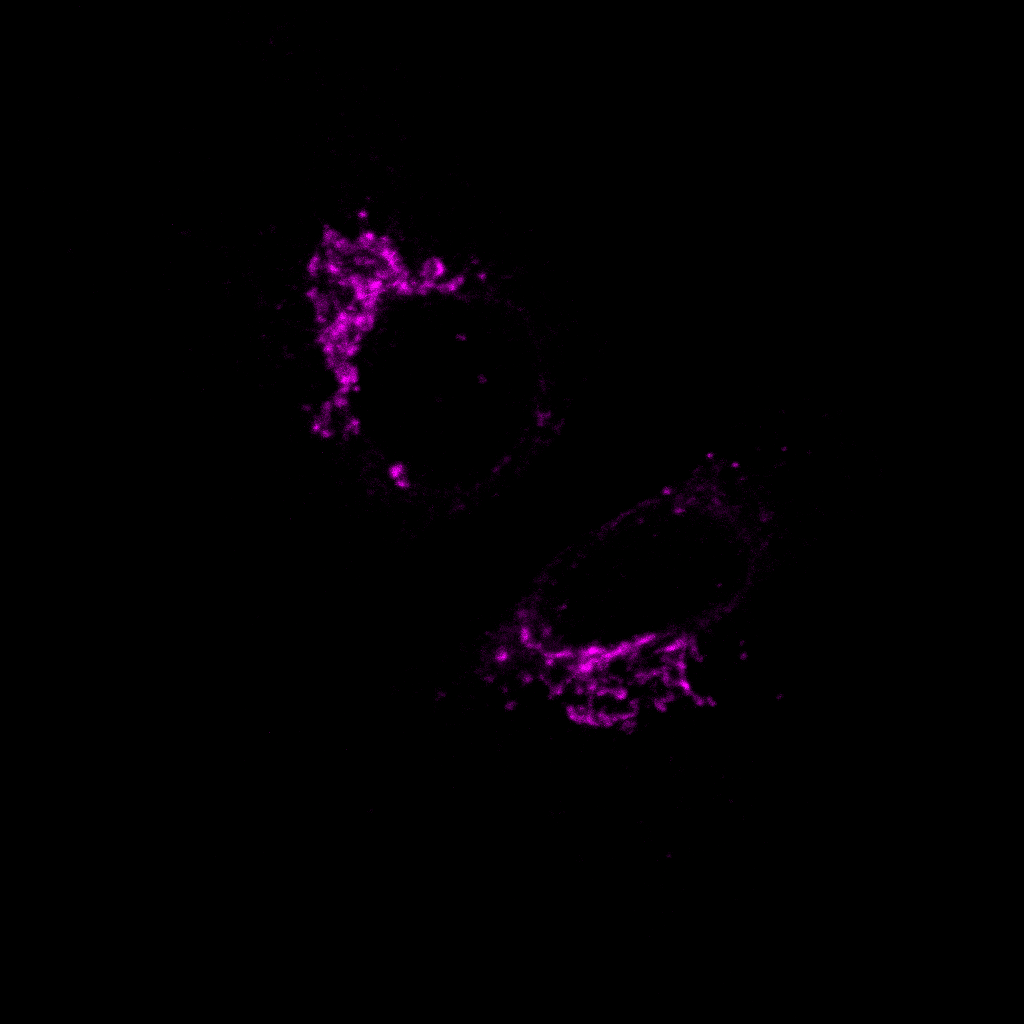

Supplement: Supplementary file 22 — EV Figure Source Data [file 44318_2024_356_MOESM22_ESM.zip › Figure EV/Appendix/Appendix 1B_GM130_Fam134b2.tif]
