# Supplementary figures and images for: Two FAM134B isoforms differentially regulate ER dynamics during myogenesis (part 2 of 2)
Source: EMBO J. 2025 Jan 6;44(4):1039–73. doi: 10.1038/s44318-024-00356-2 (PMC11832904; doi:10.1038/s44318-024-00356-2)

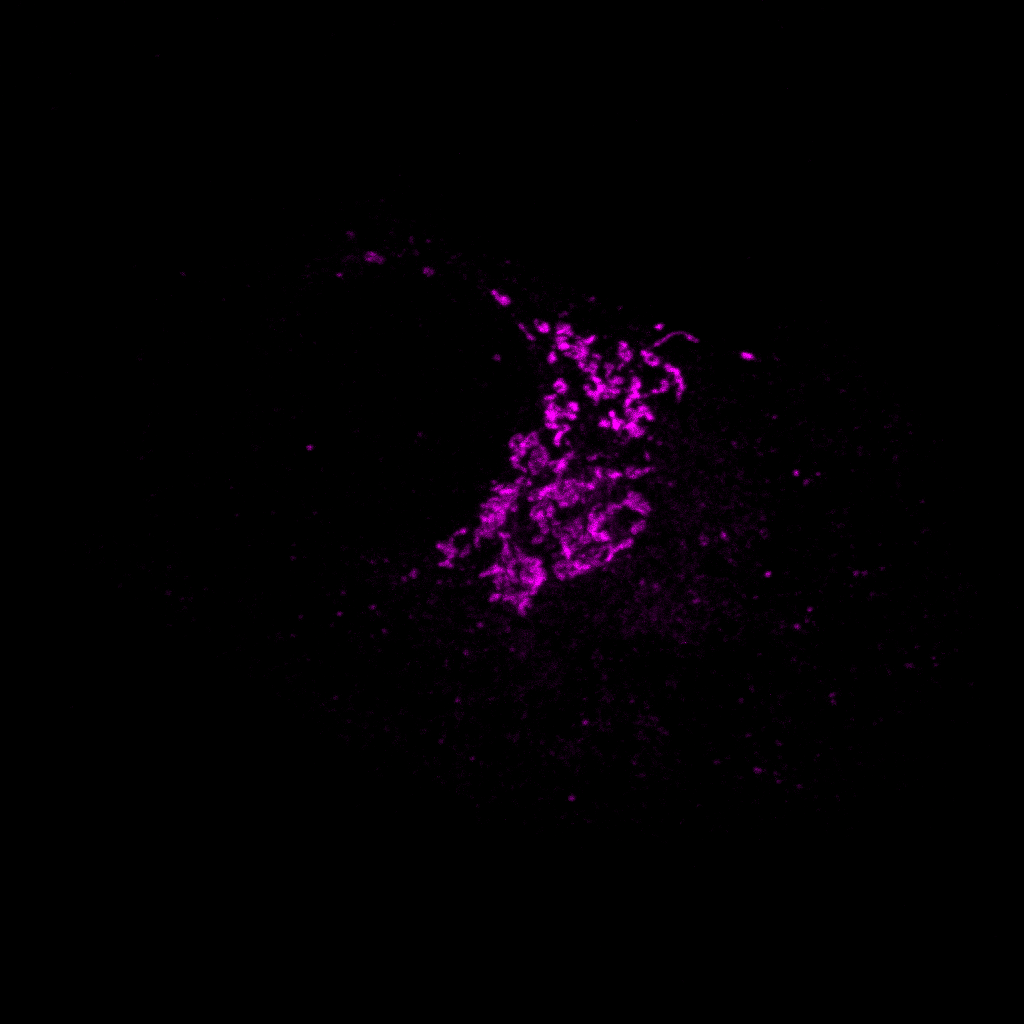

Supplement: Supplementary file 22 — EV Figure Source Data [file 44318_2024_356_MOESM22_ESM.zip › Figure EV/Appendix/Appendix 1B_GM130_Fam134b1.tif]

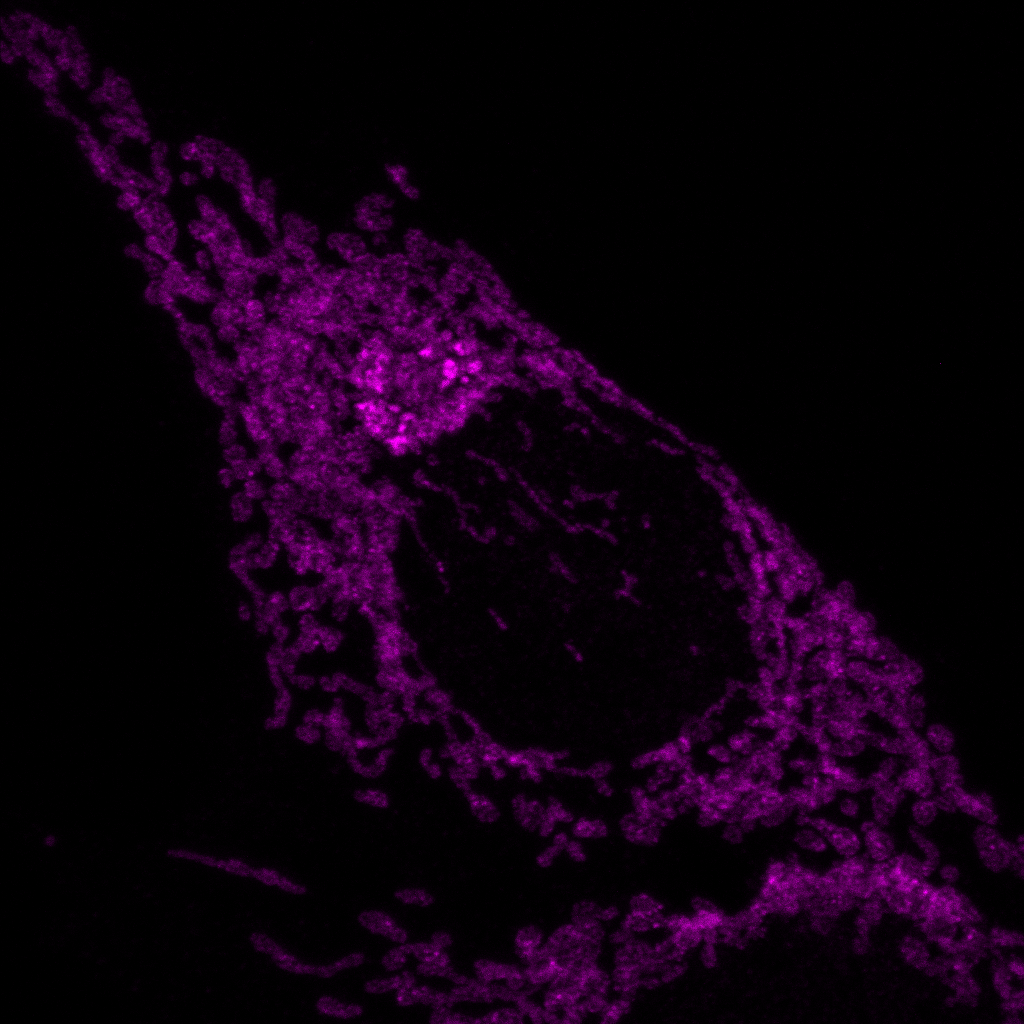

Supplement: Supplementary file 22 — EV Figure Source Data [file 44318_2024_356_MOESM22_ESM.zip › Figure EV/Appendix/Appendix 1B_TOM20_Fam134b1.tif]

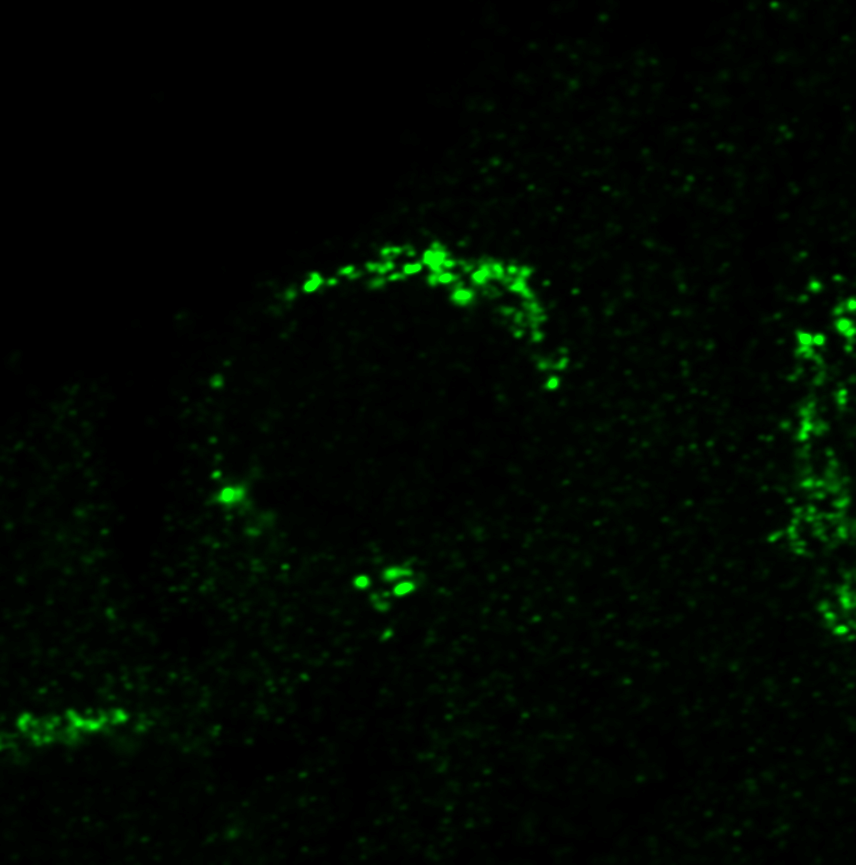

Supplement: Supplementary file 22 — EV Figure Source Data [file 44318_2024_356_MOESM22_ESM.zip › Figure EV/Appendix/Appendix 1C TGN38 Triton.tiff]

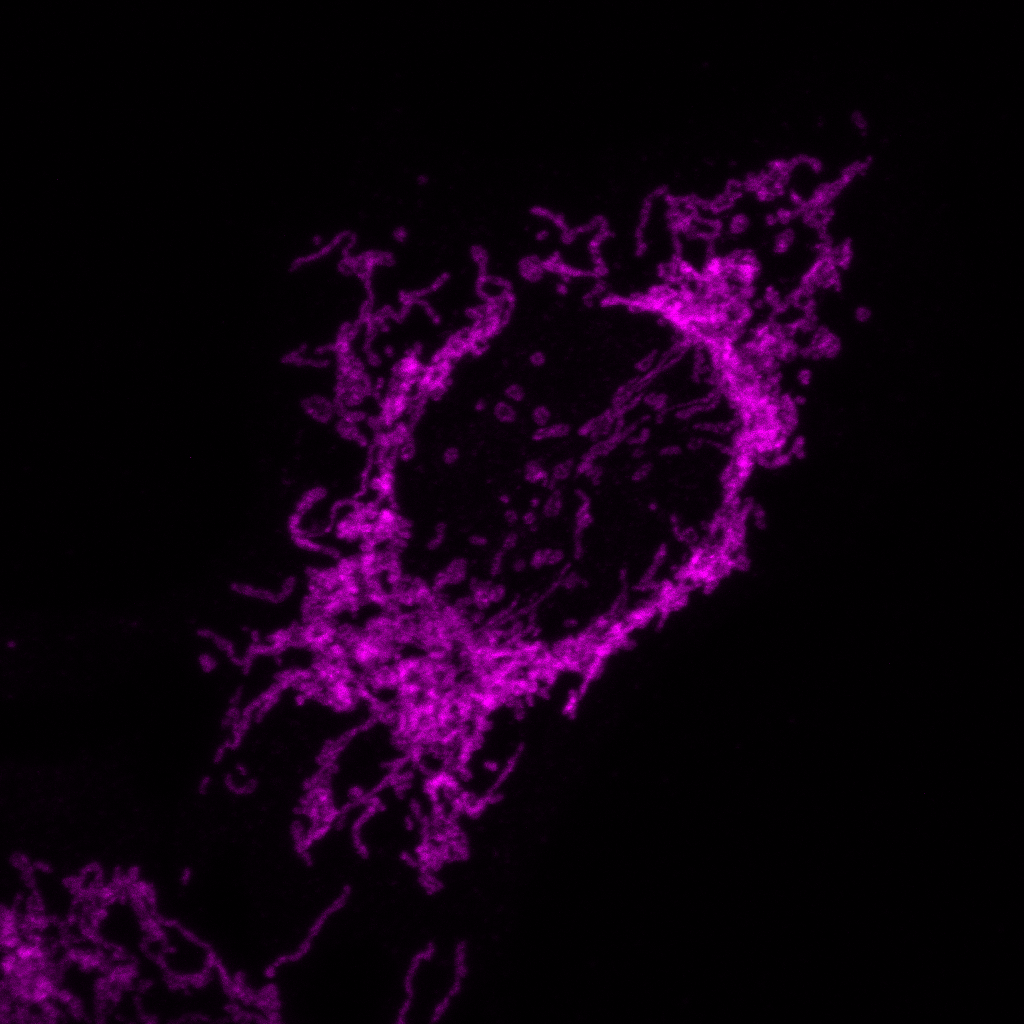

Supplement: Supplementary file 22 — EV Figure Source Data [file 44318_2024_356_MOESM22_ESM.zip › Figure EV/Appendix/Appendix 1B_Tom20_Fam134b2.tif]

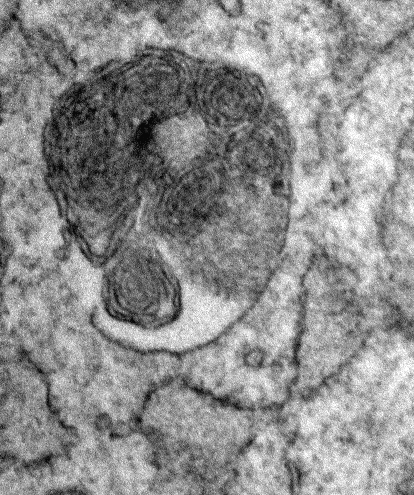

Supplement: Supplementary file 22 — EV Figure Source Data [file 44318_2024_356_MOESM22_ESM.zip › Figure EV/Fig EV5/Fig EV5F crop.tif]

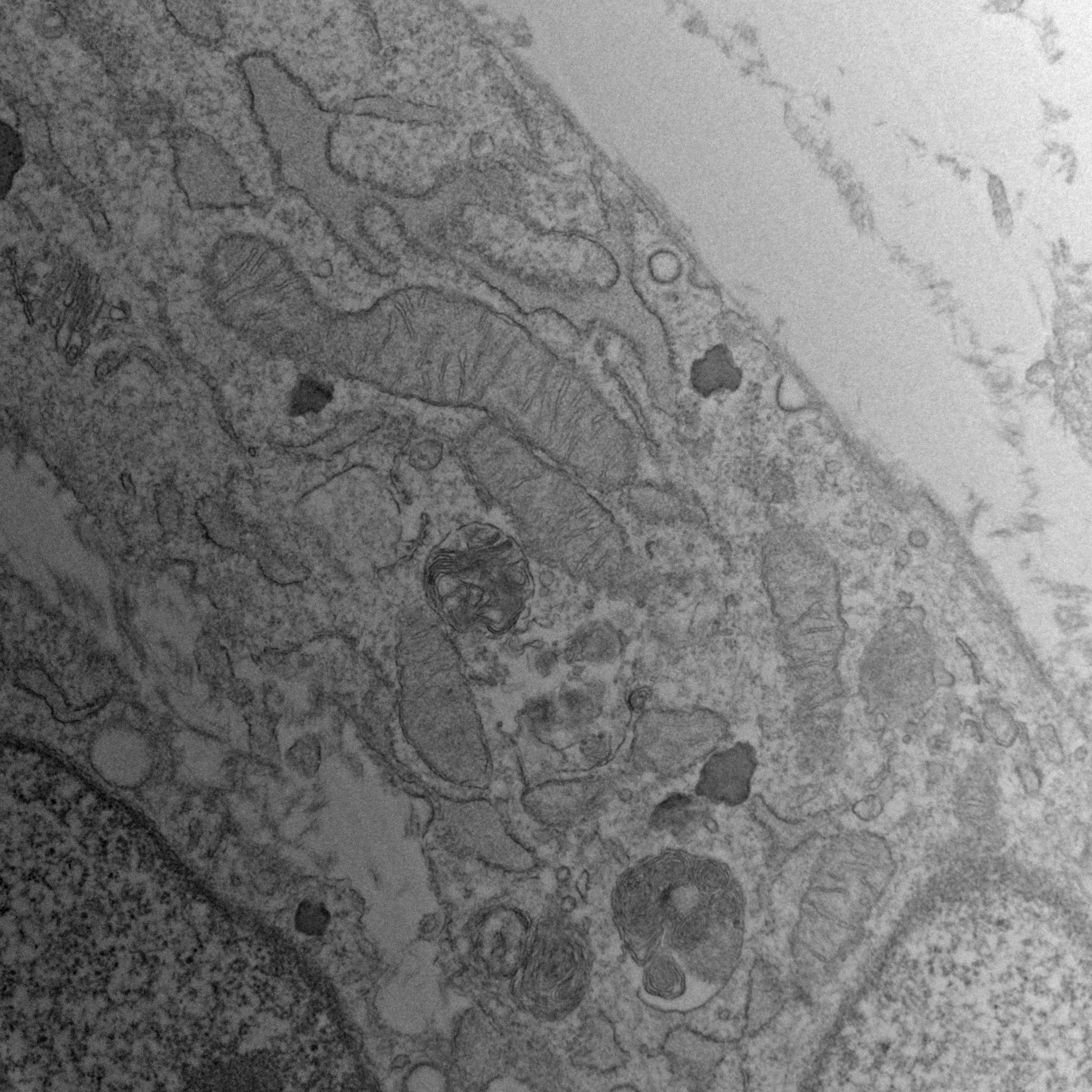

Supplement: Supplementary file 22 — EV Figure Source Data [file 44318_2024_356_MOESM22_ESM.zip › Figure EV/Fig EV5/Fig EV5F.tif]

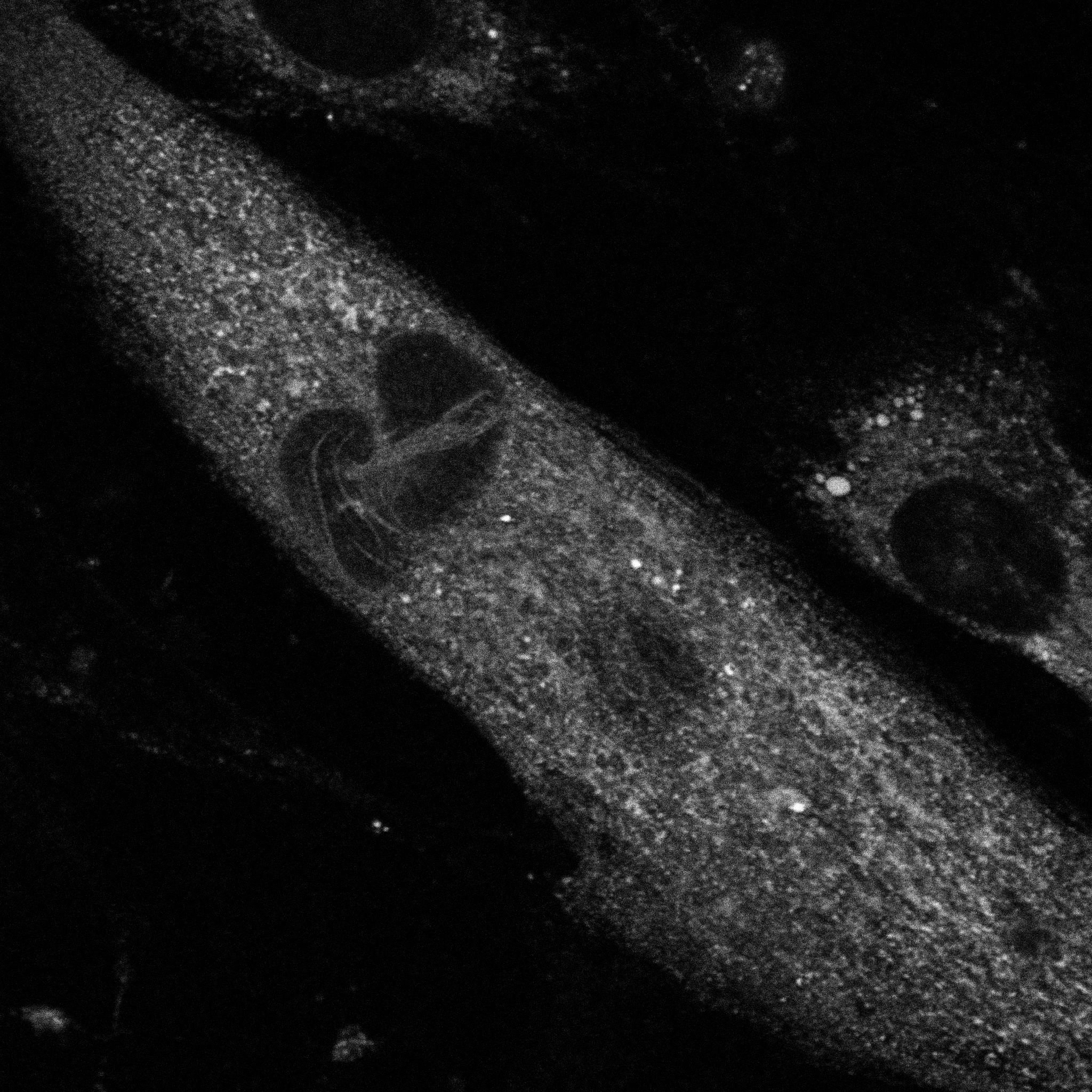

Supplement: Supplementary file 22 — EV Figure Source Data [file 44318_2024_356_MOESM22_ESM.zip › Figure EV/Fig EV2/Fig EV2D (day5).tif]

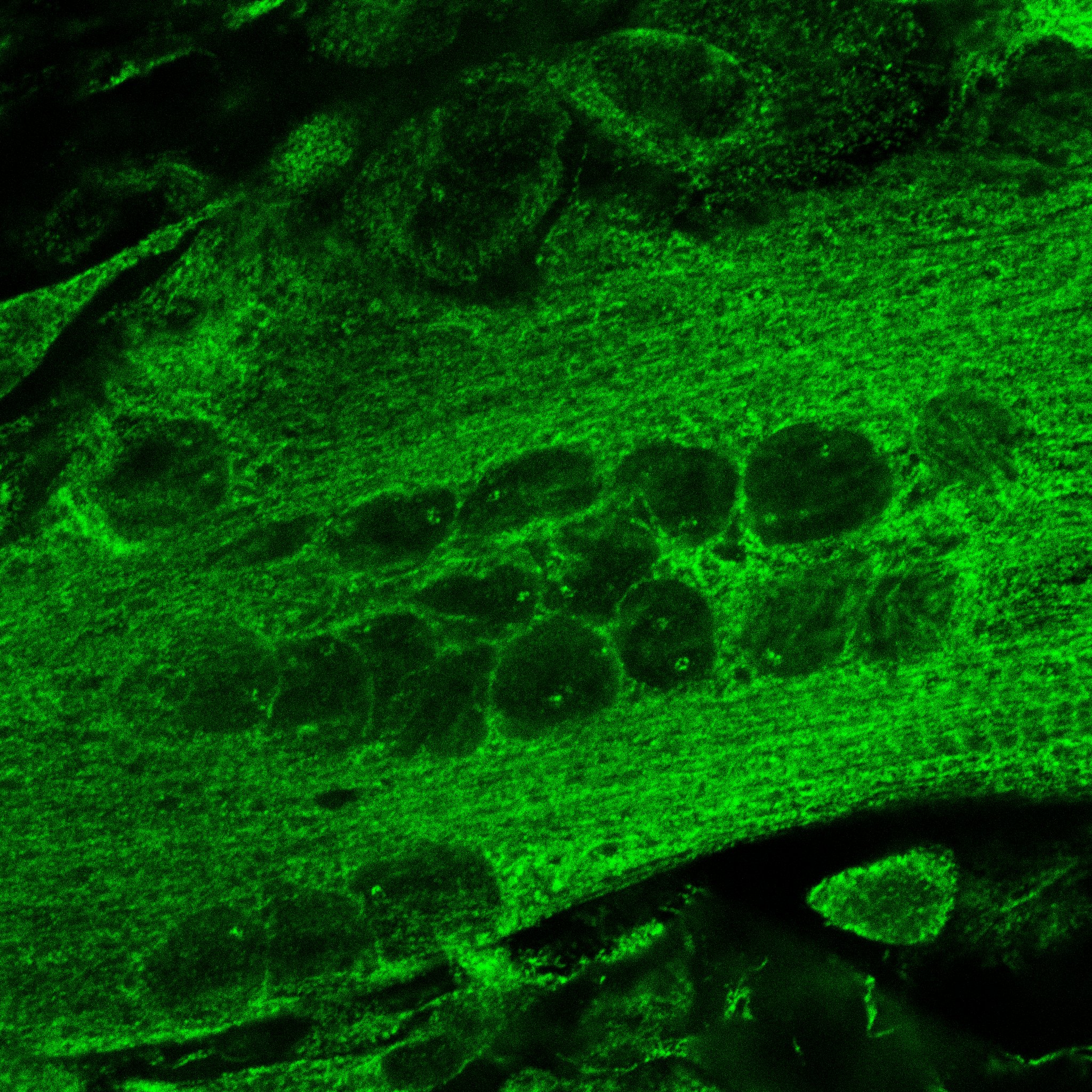

Supplement: Supplementary file 22 — EV Figure Source Data [file 44318_2024_356_MOESM22_ESM.zip › Figure EV/Fig EV2/Fig EV2C Reep5.jpg]

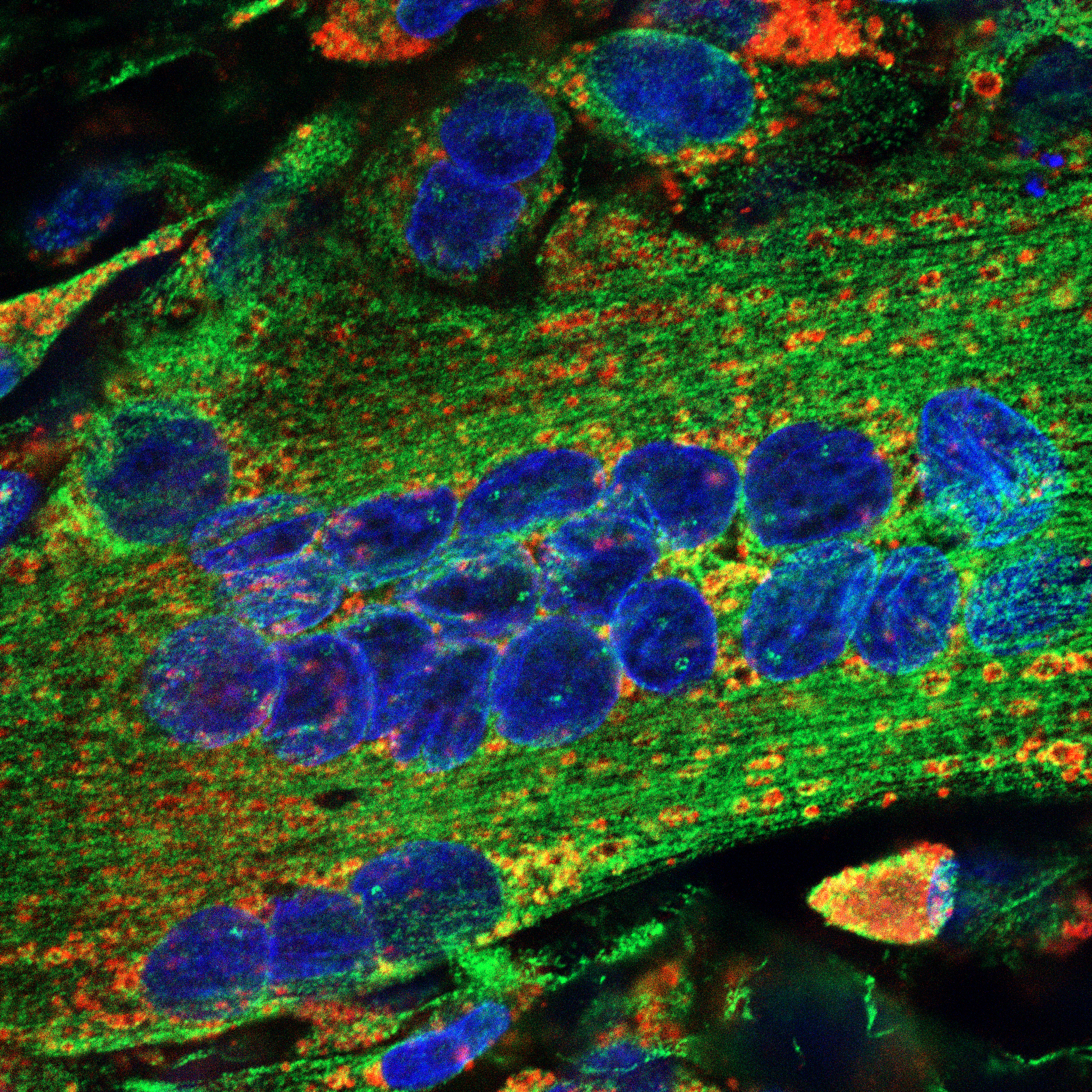

Supplement: Supplementary file 22 — EV Figure Source Data [file 44318_2024_356_MOESM22_ESM.zip › Figure EV/Fig EV2/Fig EV2C Merge.tif]

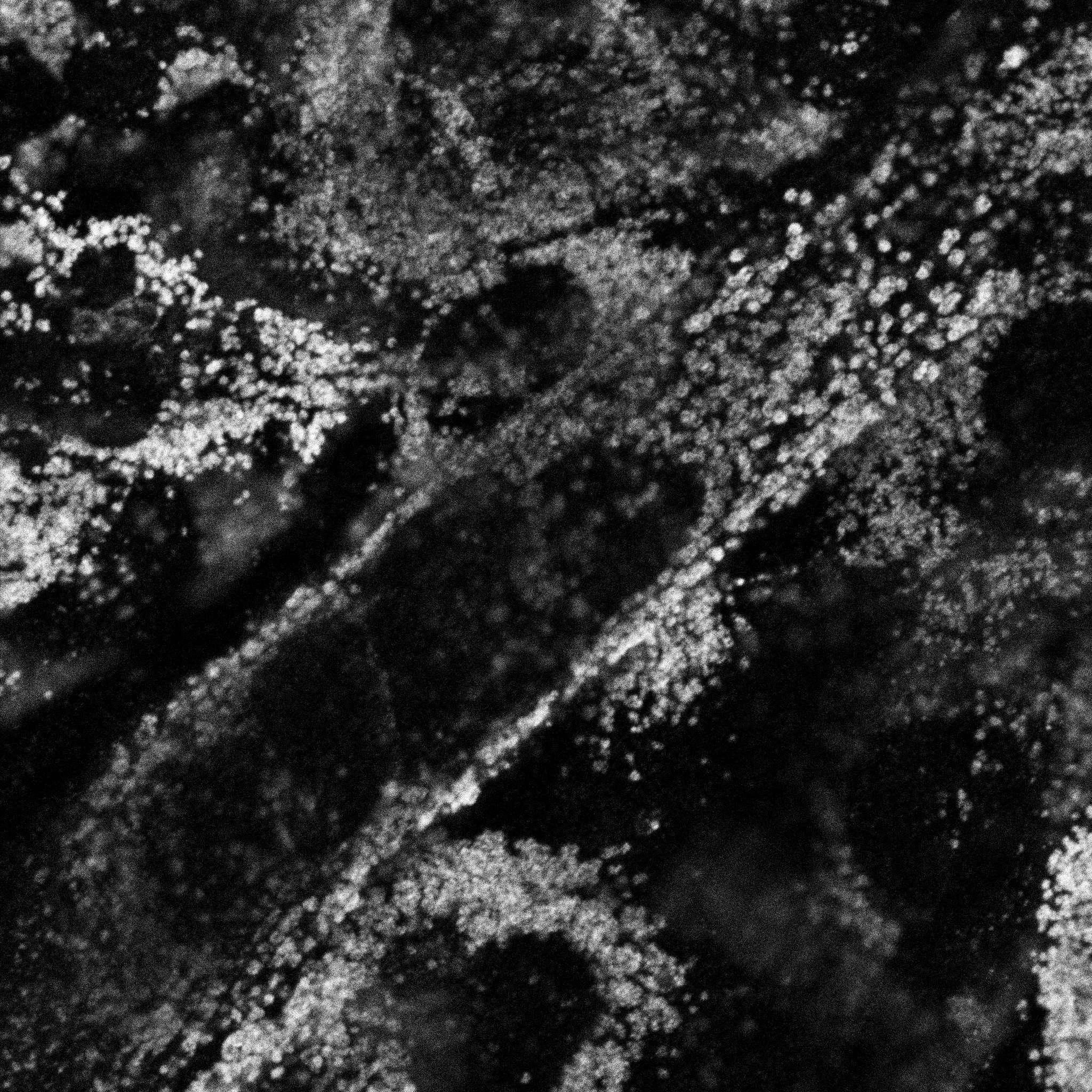

Supplement: Supplementary file 22 — EV Figure Source Data [file 44318_2024_356_MOESM22_ESM.zip › Figure EV/Fig EV2/Fig EV2B (Reep5-Lamp1).tif]

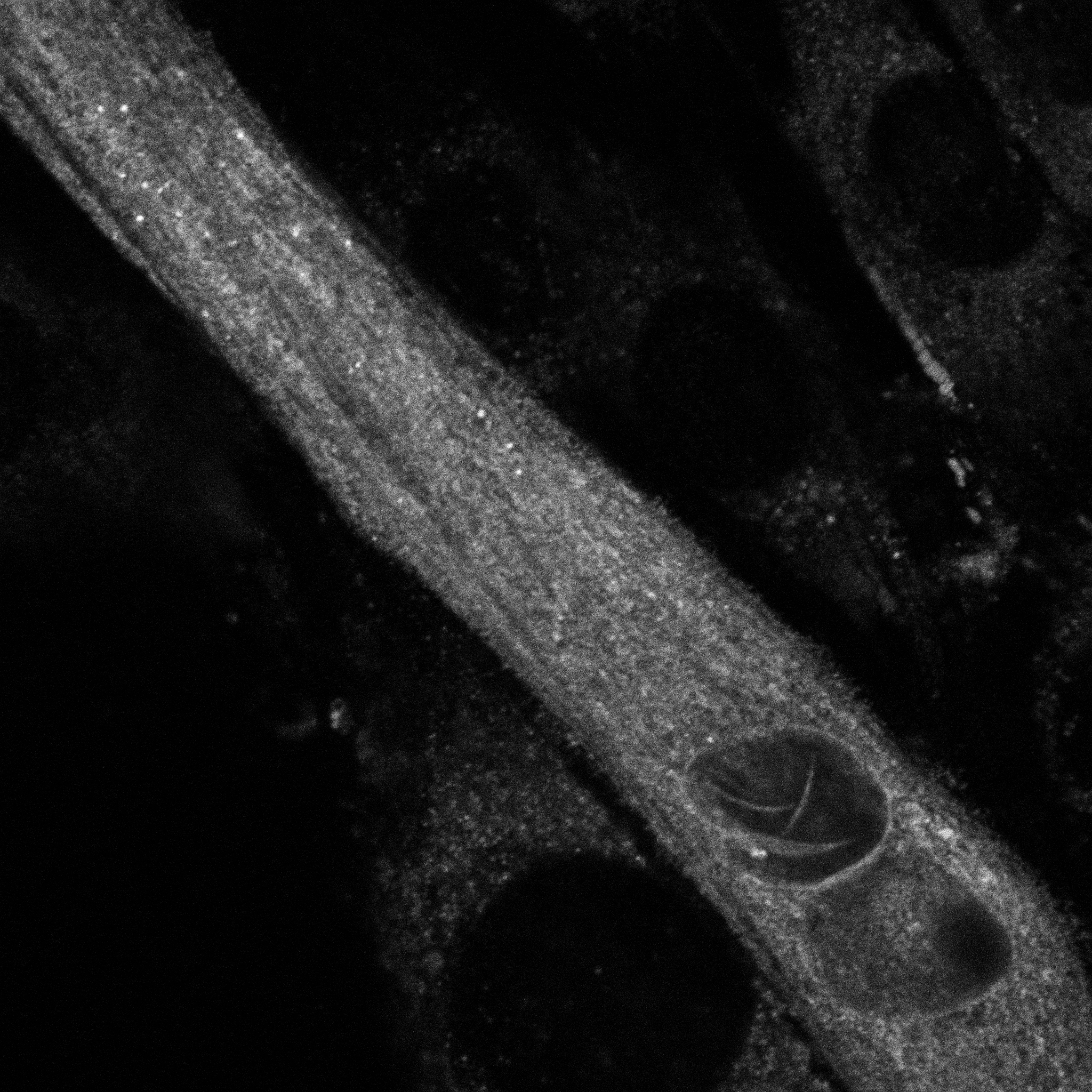

Supplement: Supplementary file 22 — EV Figure Source Data [file 44318_2024_356_MOESM22_ESM.zip › Figure EV/Fig EV2/Fig EV2D (day7).tif]

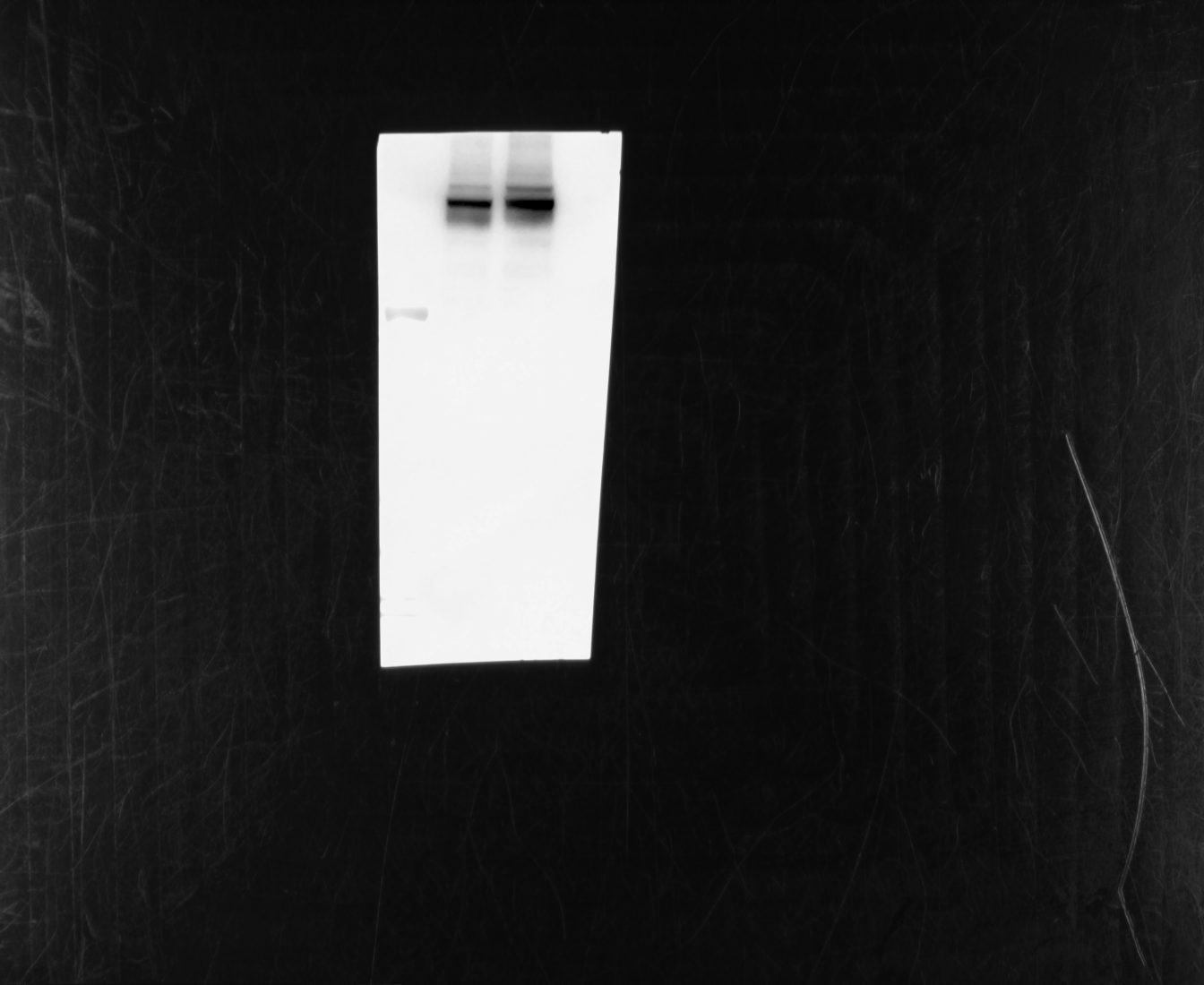

Supplement: Supplementary file 22 — EV Figure Source Data [file 44318_2024_356_MOESM22_ESM.zip › Figure EV/Fig EV2/Fig EV2G Vinculin.Tif]

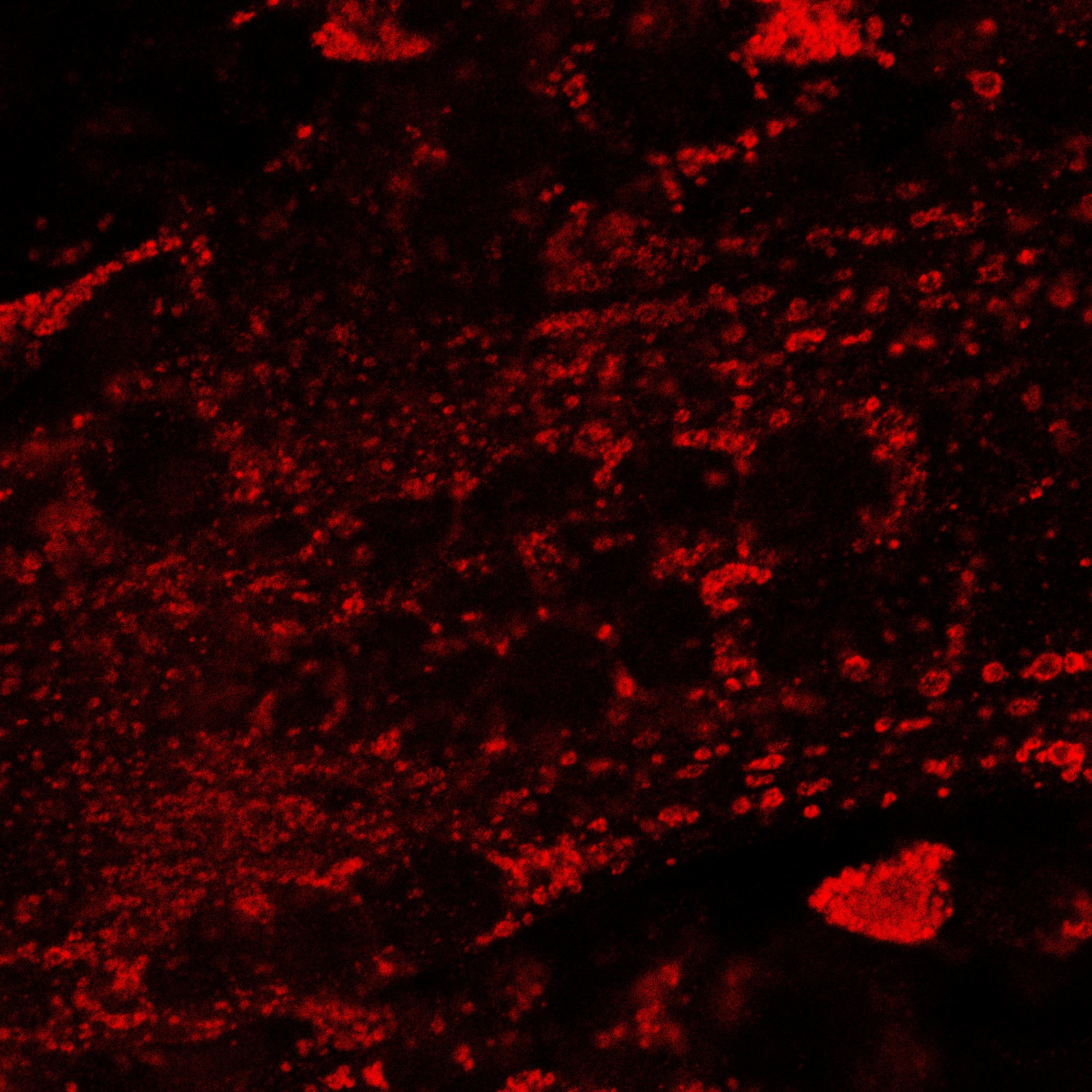

Supplement: Supplementary file 22 — EV Figure Source Data [file 44318_2024_356_MOESM22_ESM.zip › Figure EV/Fig EV2/Fig EV2C Lamp1.jpg]

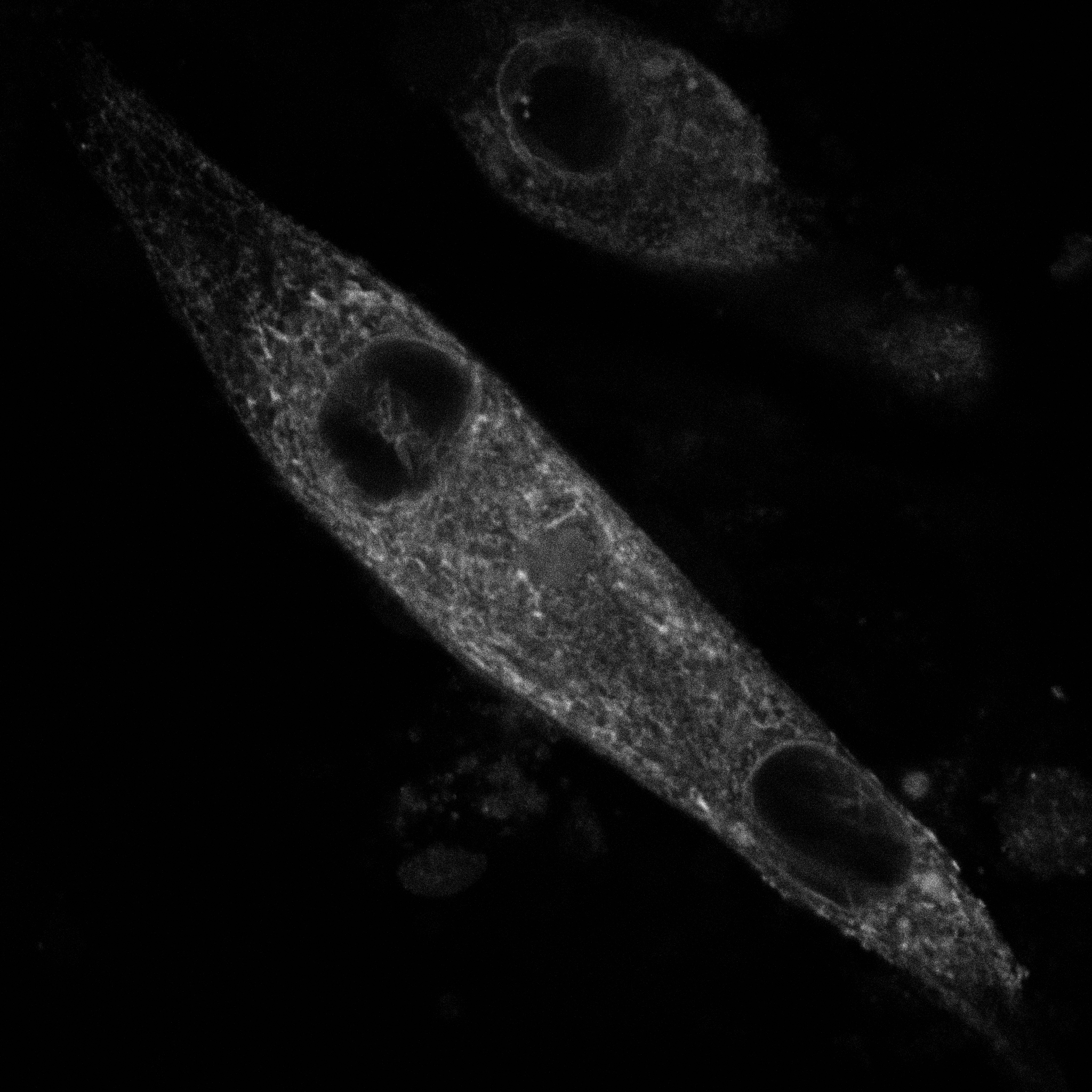

Supplement: Supplementary file 22 — EV Figure Source Data [file 44318_2024_356_MOESM22_ESM.zip › Figure EV/Fig EV2/Fig EV2D (day10).tif]

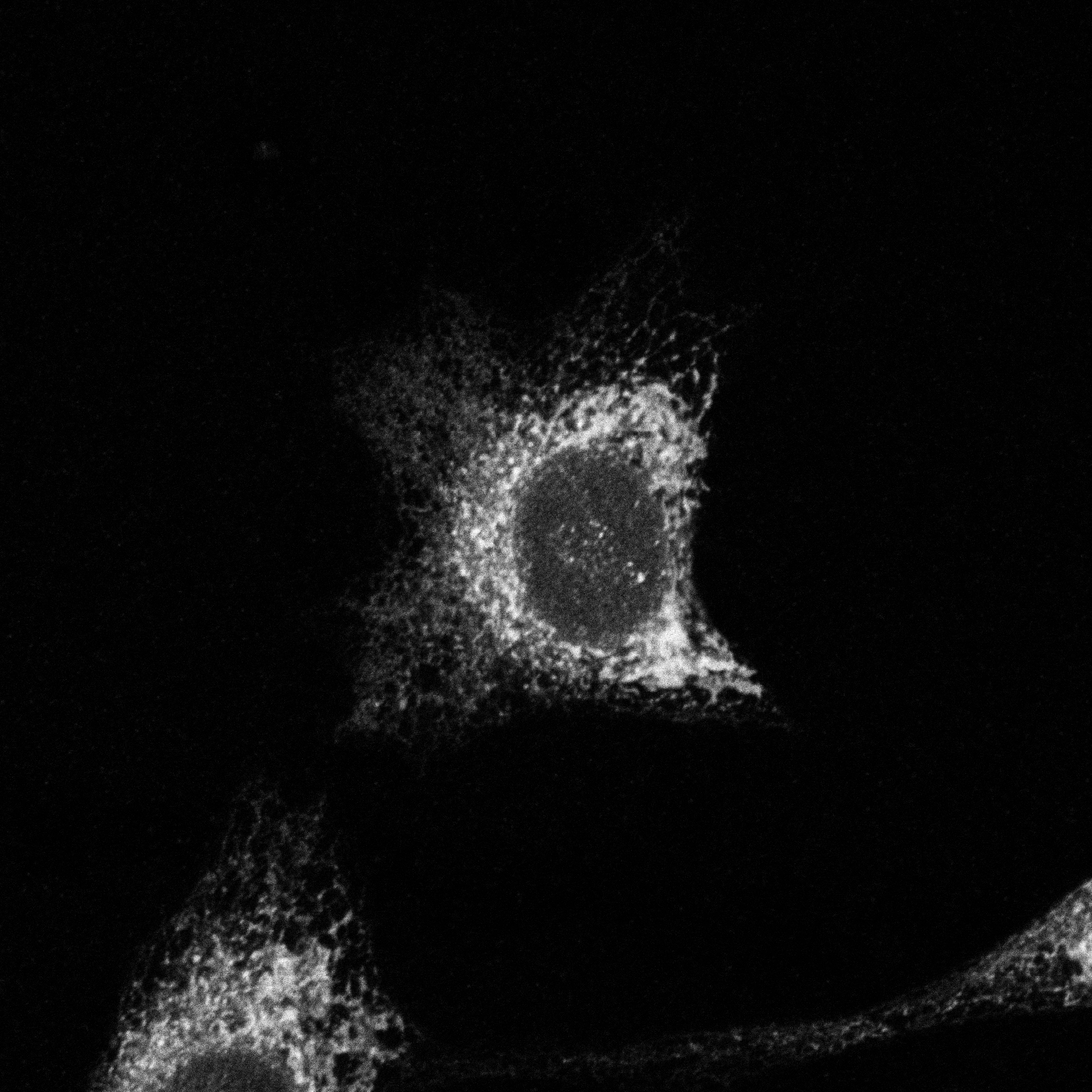

Supplement: Supplementary file 22 — EV Figure Source Data [file 44318_2024_356_MOESM22_ESM.zip › Figure EV/Fig EV2/Fig EV2D (day1).tif]

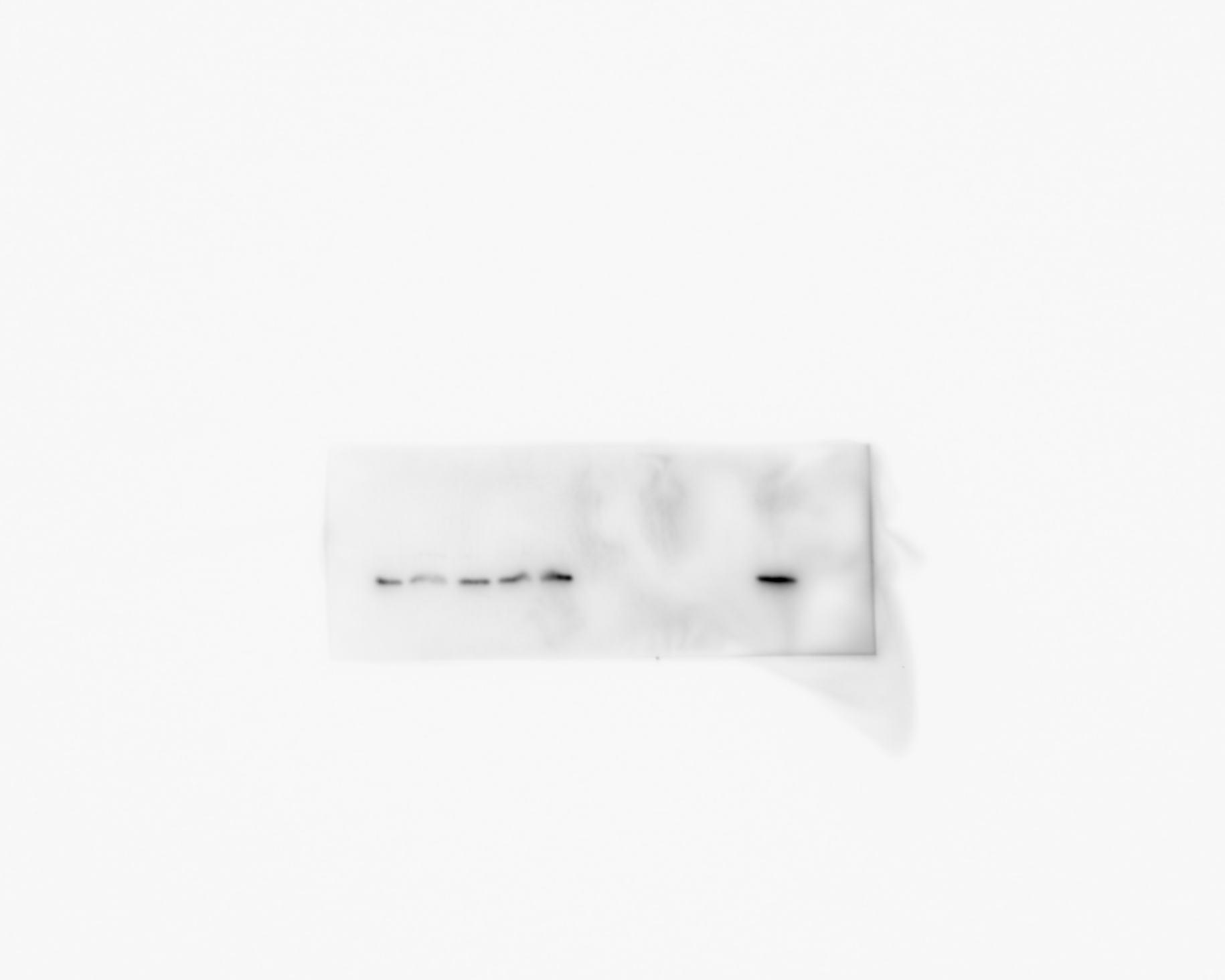

Supplement: Supplementary file 22 — EV Figure Source Data [file 44318_2024_356_MOESM22_ESM.zip › Figure EV/Fig EV3/Fig EV3G LC3B.tif]

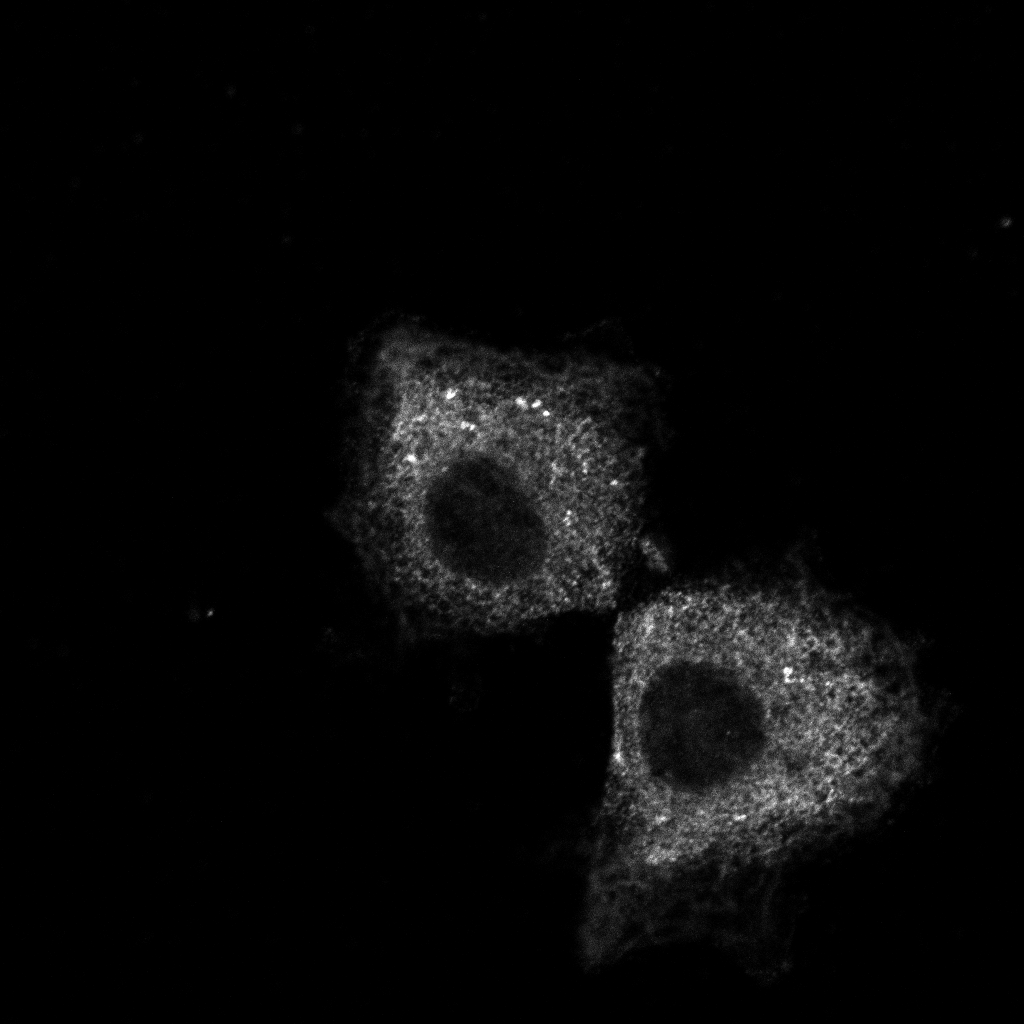

Supplement: Supplementary file 22 — EV Figure Source Data [file 44318_2024_356_MOESM22_ESM.zip › Figure EV/Fig EV3/Fig EV3A Fam134b2-Reep5.tif]

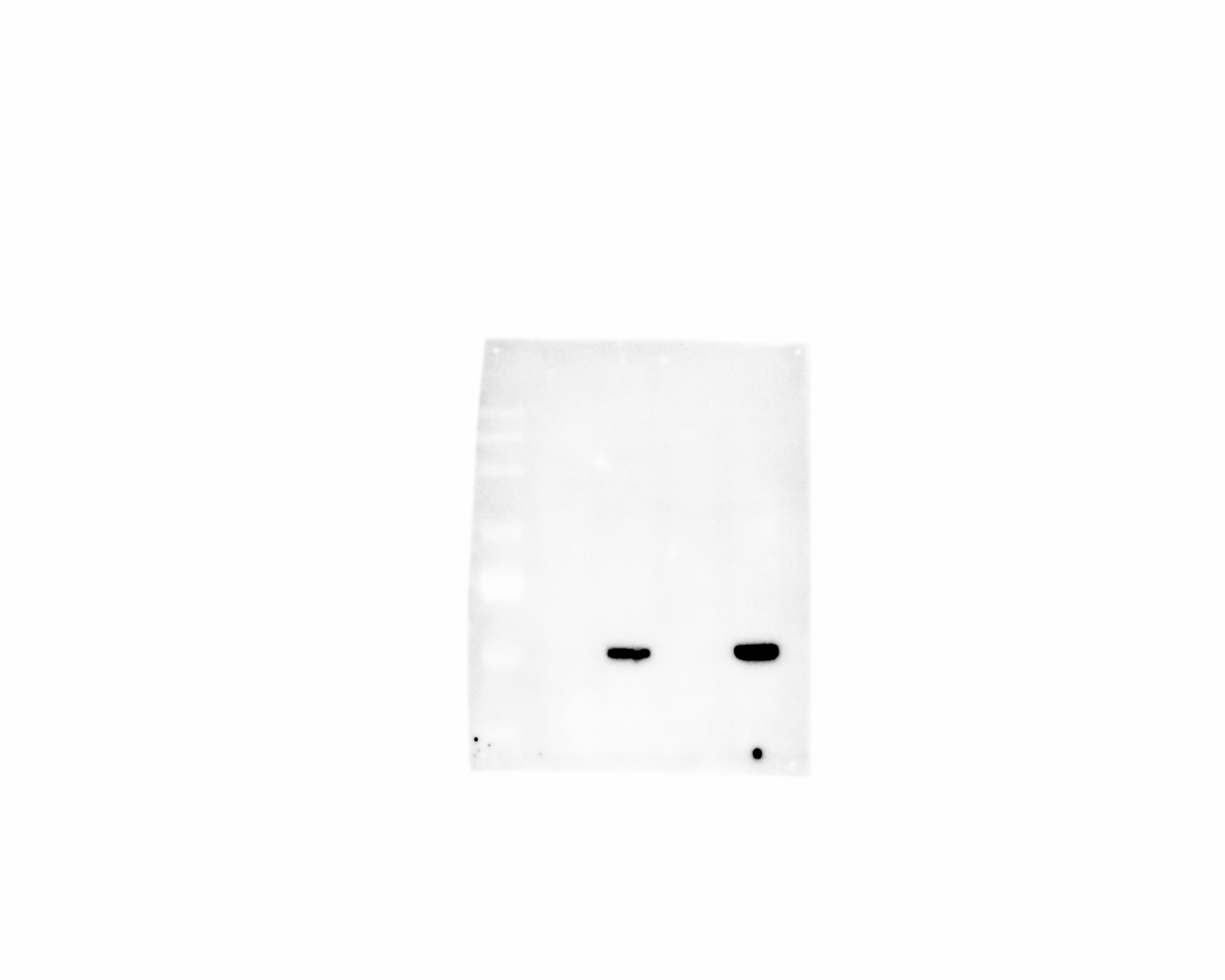

Supplement: Supplementary file 22 — EV Figure Source Data [file 44318_2024_356_MOESM22_ESM.zip › Figure EV/Fig EV3/Fig EV3B HA.tif]

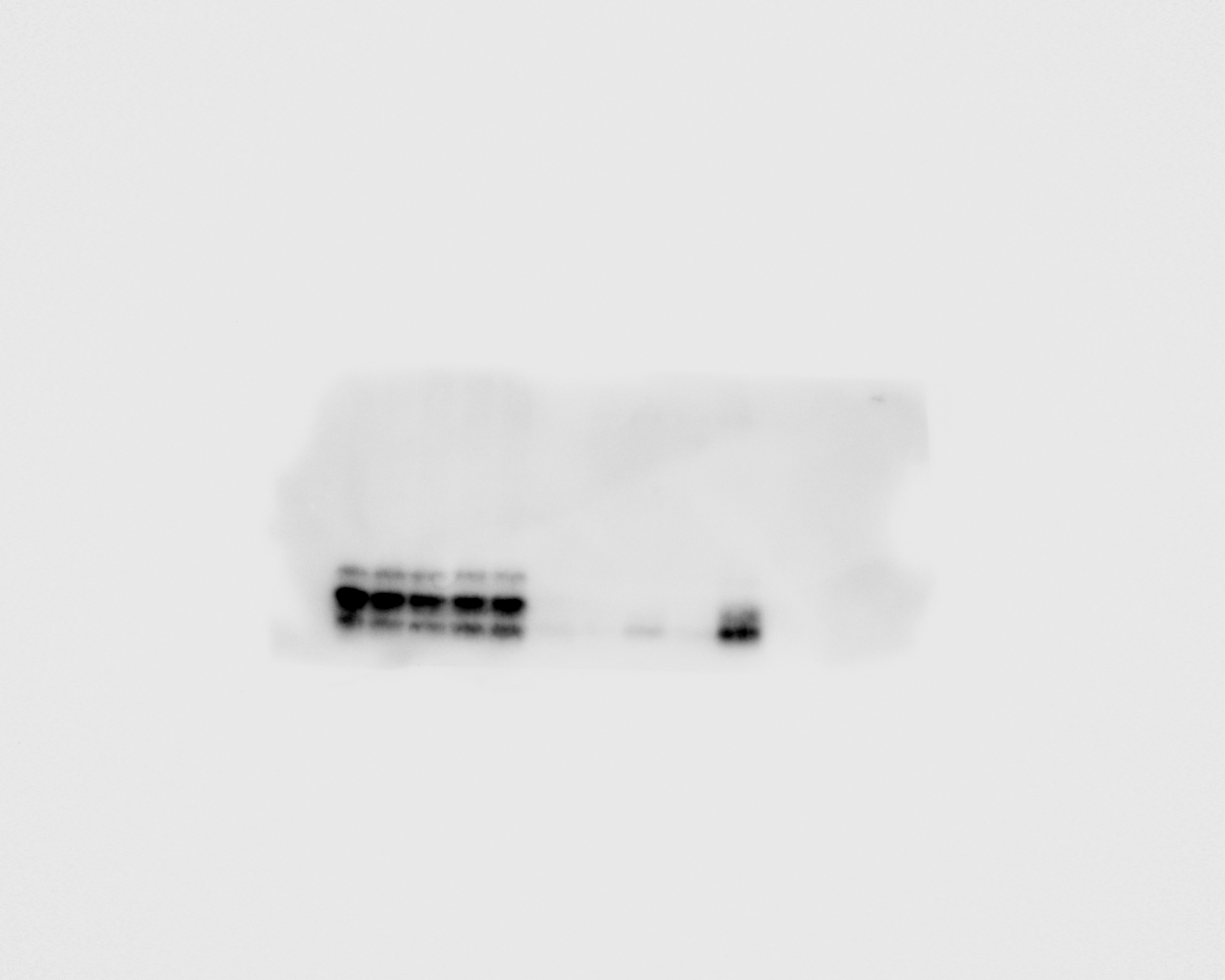

Supplement: Supplementary file 22 — EV Figure Source Data [file 44318_2024_356_MOESM22_ESM.zip › Figure EV/Fig EV3/Fig EV3G Gabarap.tif]

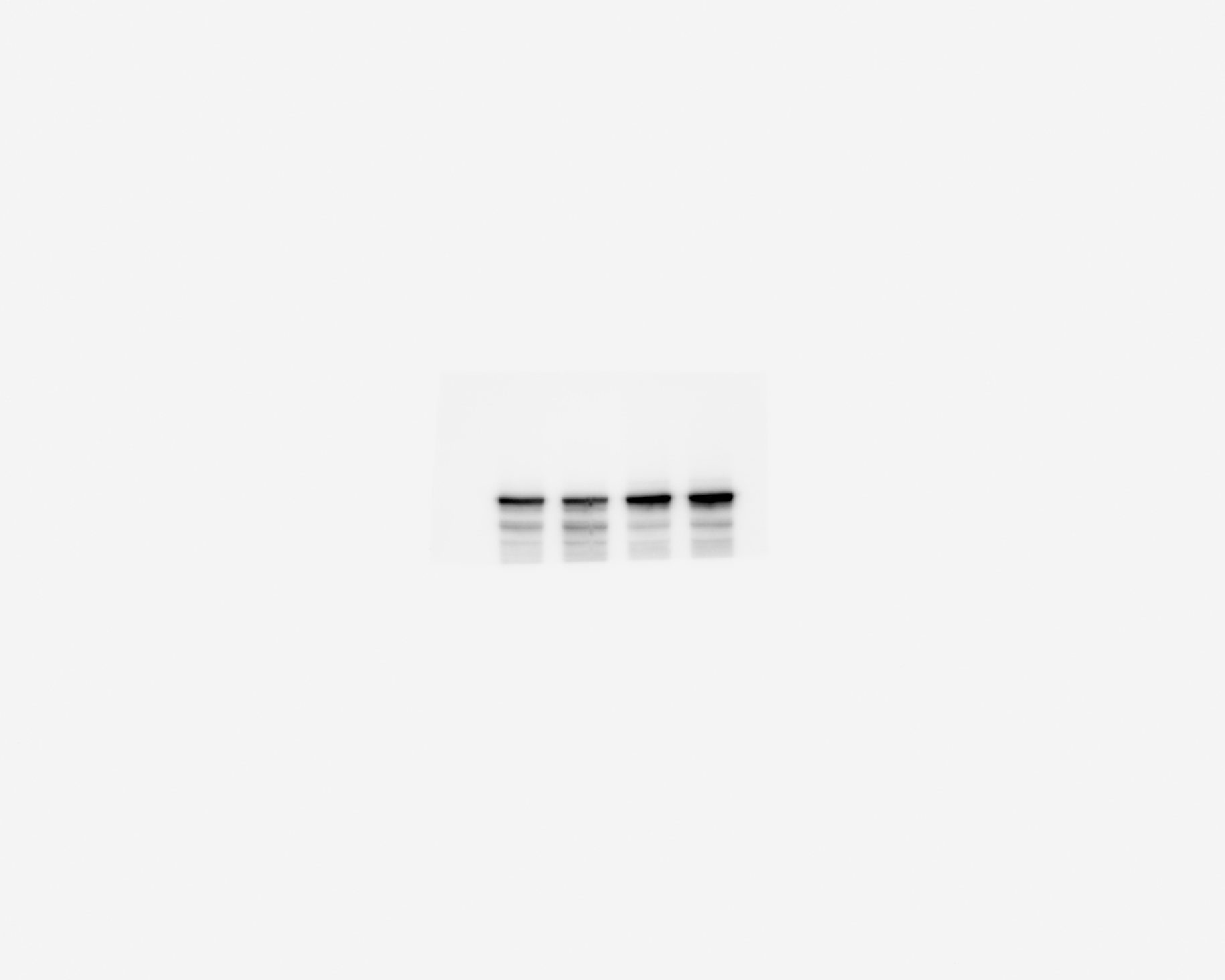

Supplement: Supplementary file 22 — EV Figure Source Data [file 44318_2024_356_MOESM22_ESM.zip › Figure EV/Fig EV3/Fig EV3B Vinculin.tif]

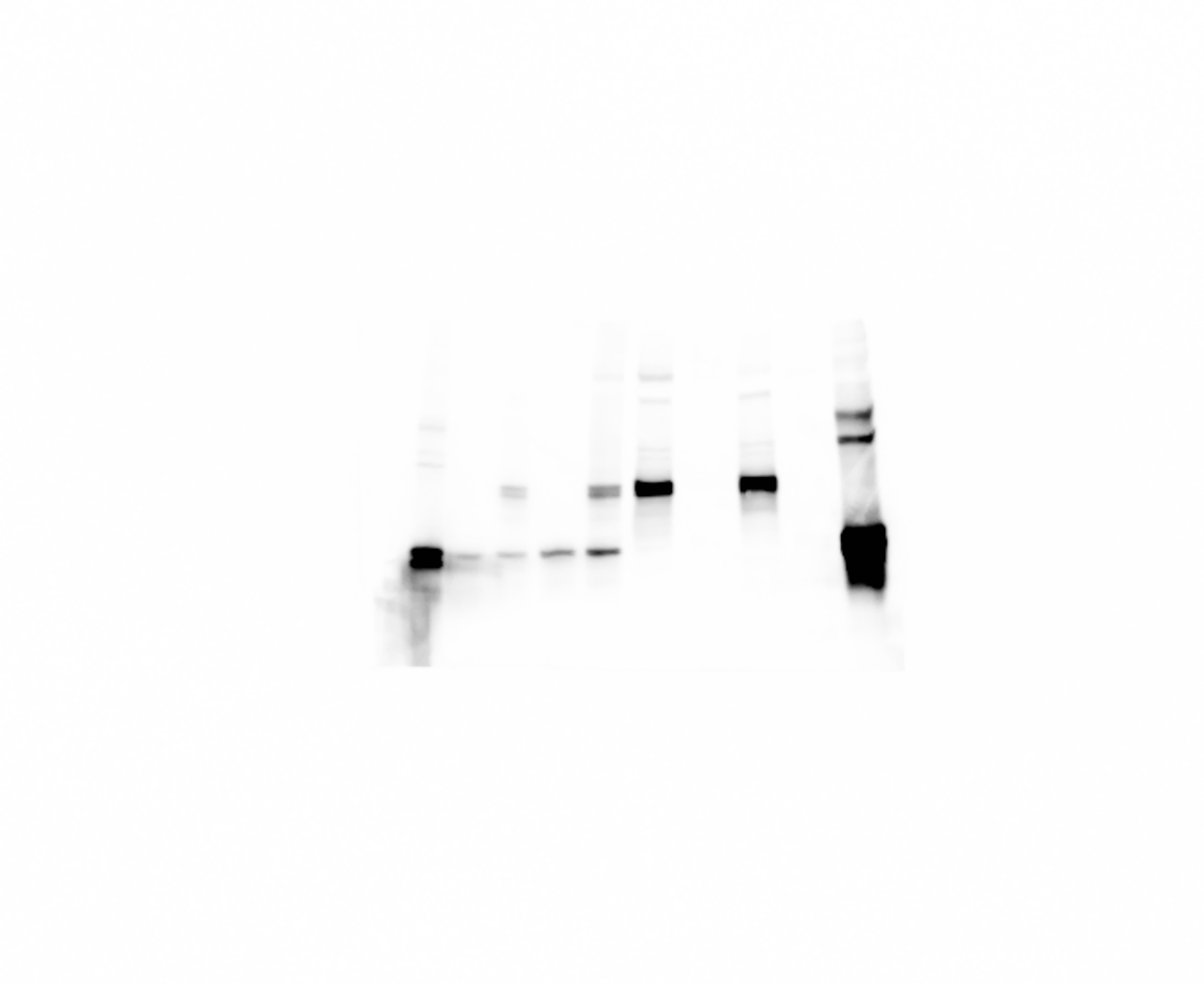

Supplement: Supplementary file 22 — EV Figure Source Data [file 44318_2024_356_MOESM22_ESM.zip › Figure EV/Fig EV3/Fig EV3G Fam134b.tif]

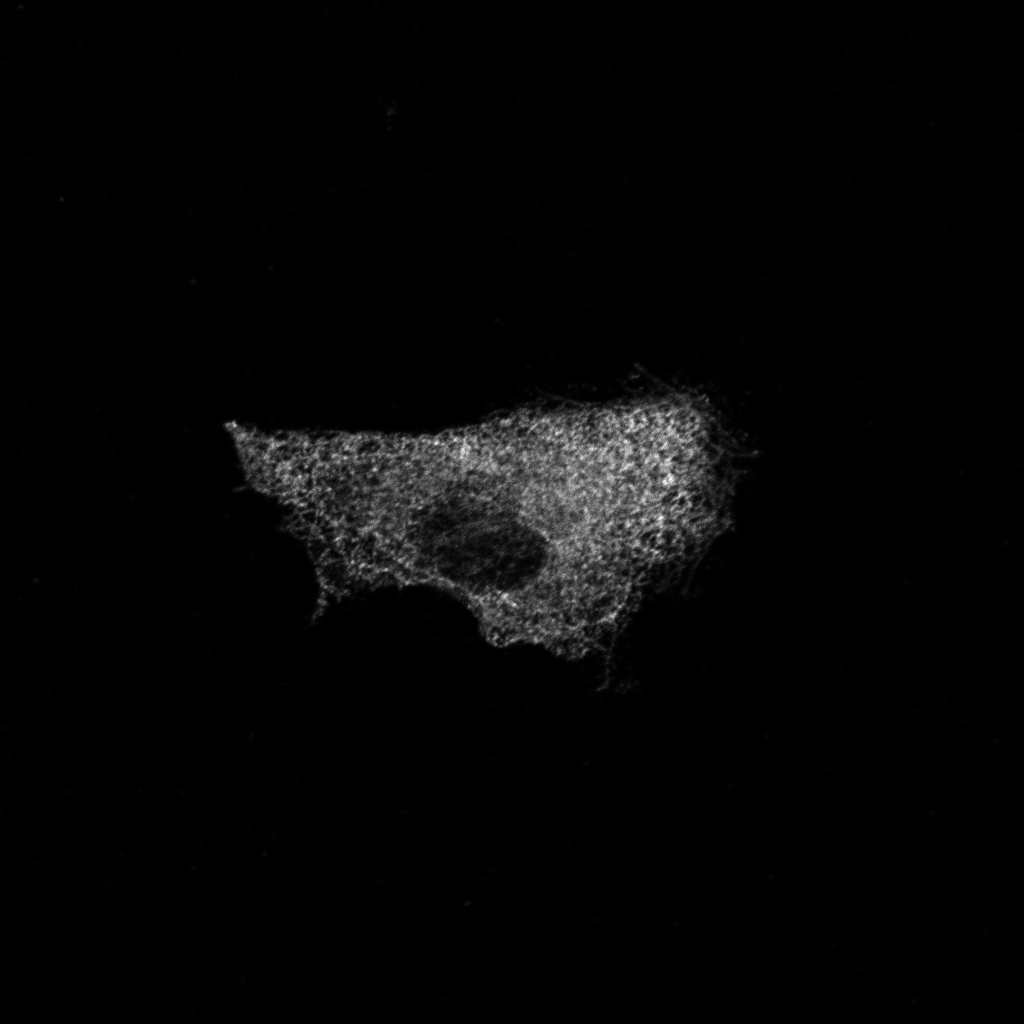

Supplement: Supplementary file 22 — EV Figure Source Data [file 44318_2024_356_MOESM22_ESM.zip › Figure EV/Fig EV3/Fig EV3A Fam134b2LIR-REEP5.tif]

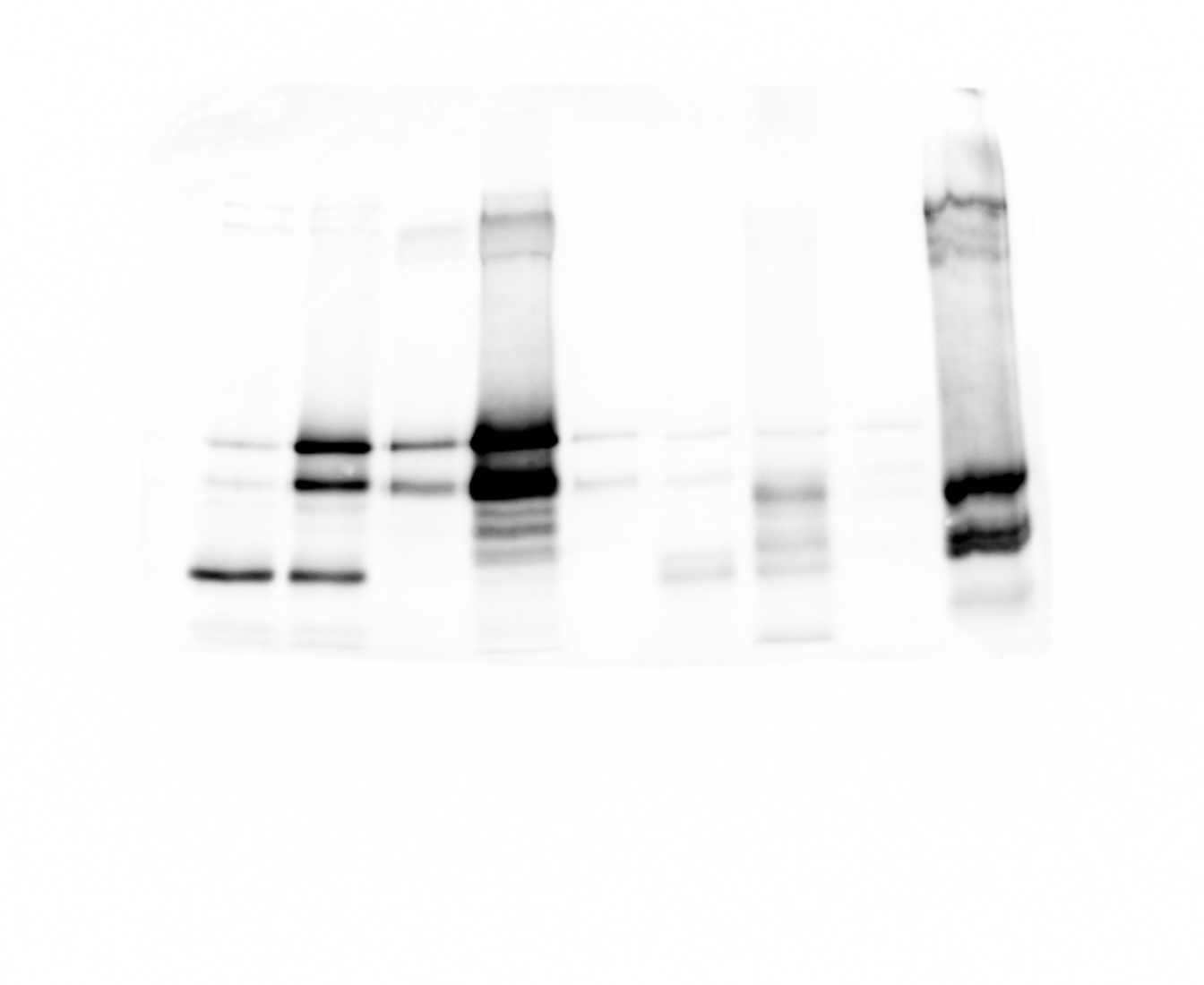

Supplement: Supplementary file 22 — EV Figure Source Data [file 44318_2024_356_MOESM22_ESM.zip › Figure EV/Fig EV3/Fig EV3D.tif]

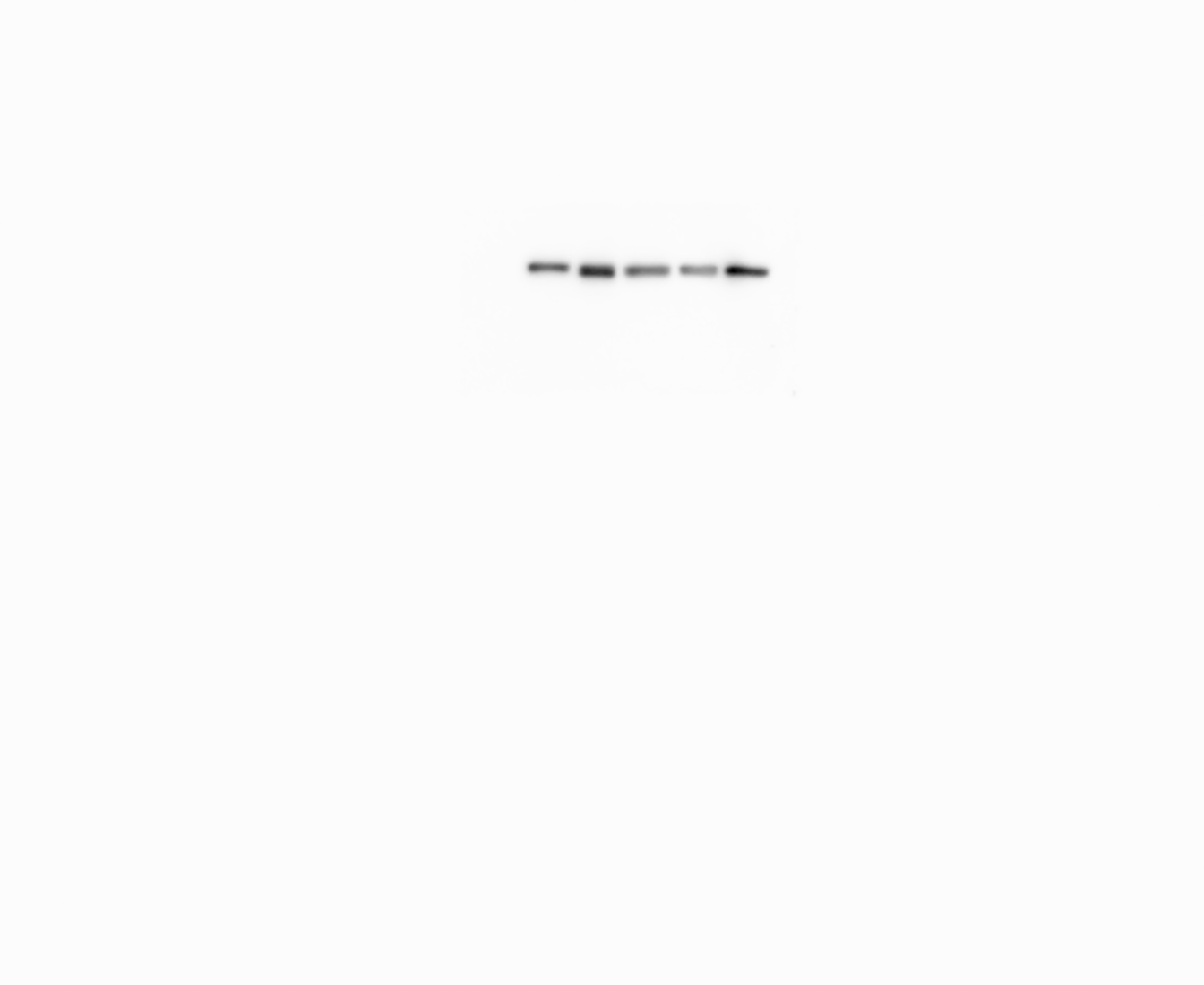

Supplement: Supplementary file 22 — EV Figure Source Data [file 44318_2024_356_MOESM22_ESM.zip › Figure EV/Fig EV4/Fig EV4A VAPA.tif]

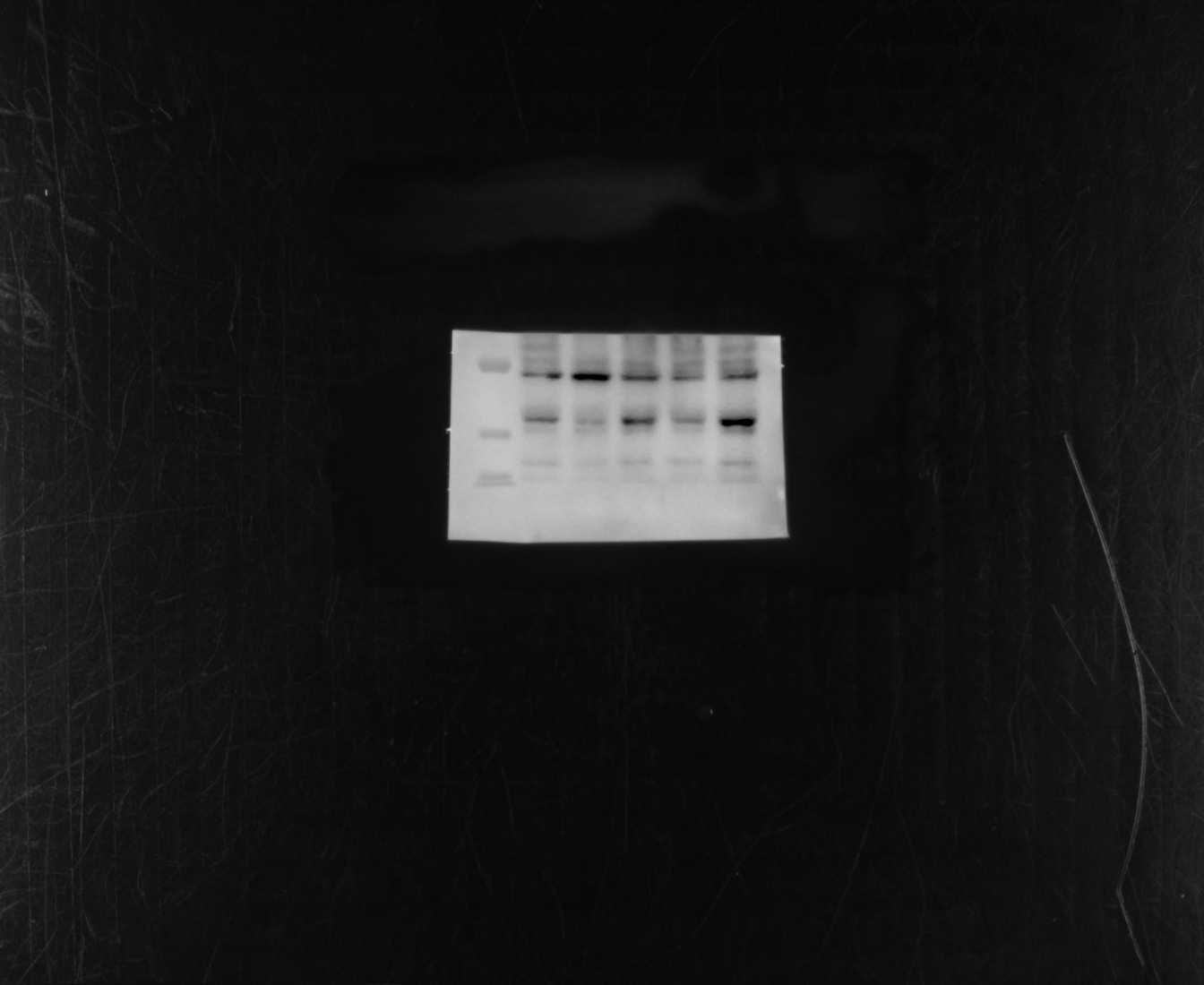

Supplement: Supplementary file 22 — EV Figure Source Data [file 44318_2024_356_MOESM22_ESM.zip › Figure EV/Fig EV4/Fig EV4A VAPB.Tif]

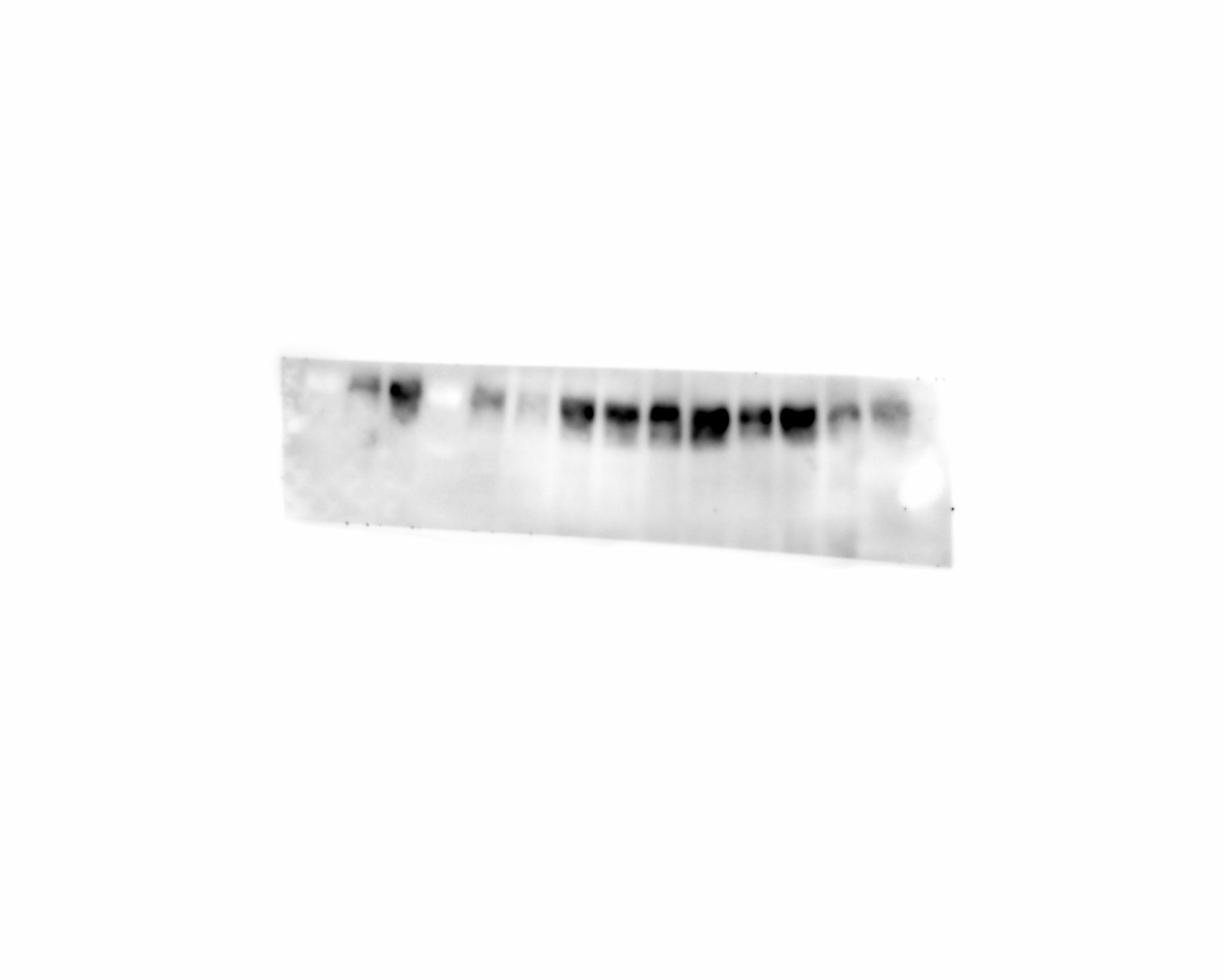

Supplement: Supplementary file 22 — EV Figure Source Data [file 44318_2024_356_MOESM22_ESM.zip › Figure EV/Fig EV4/Fig EV4F MEF2C.tif]

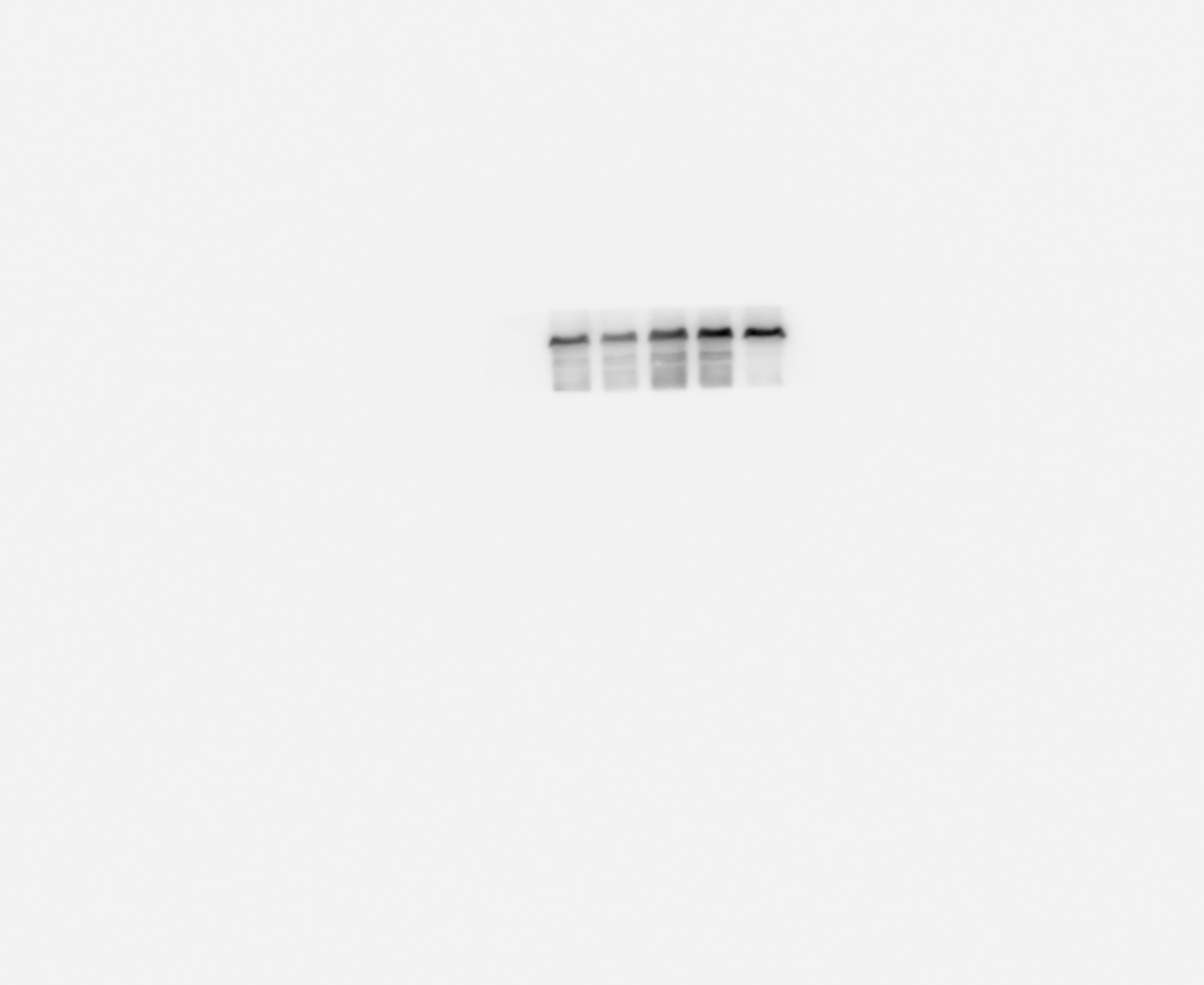

Supplement: Supplementary file 22 — EV Figure Source Data [file 44318_2024_356_MOESM22_ESM.zip › Figure EV/Fig EV4/Fig EV4A Vinculin.tif]

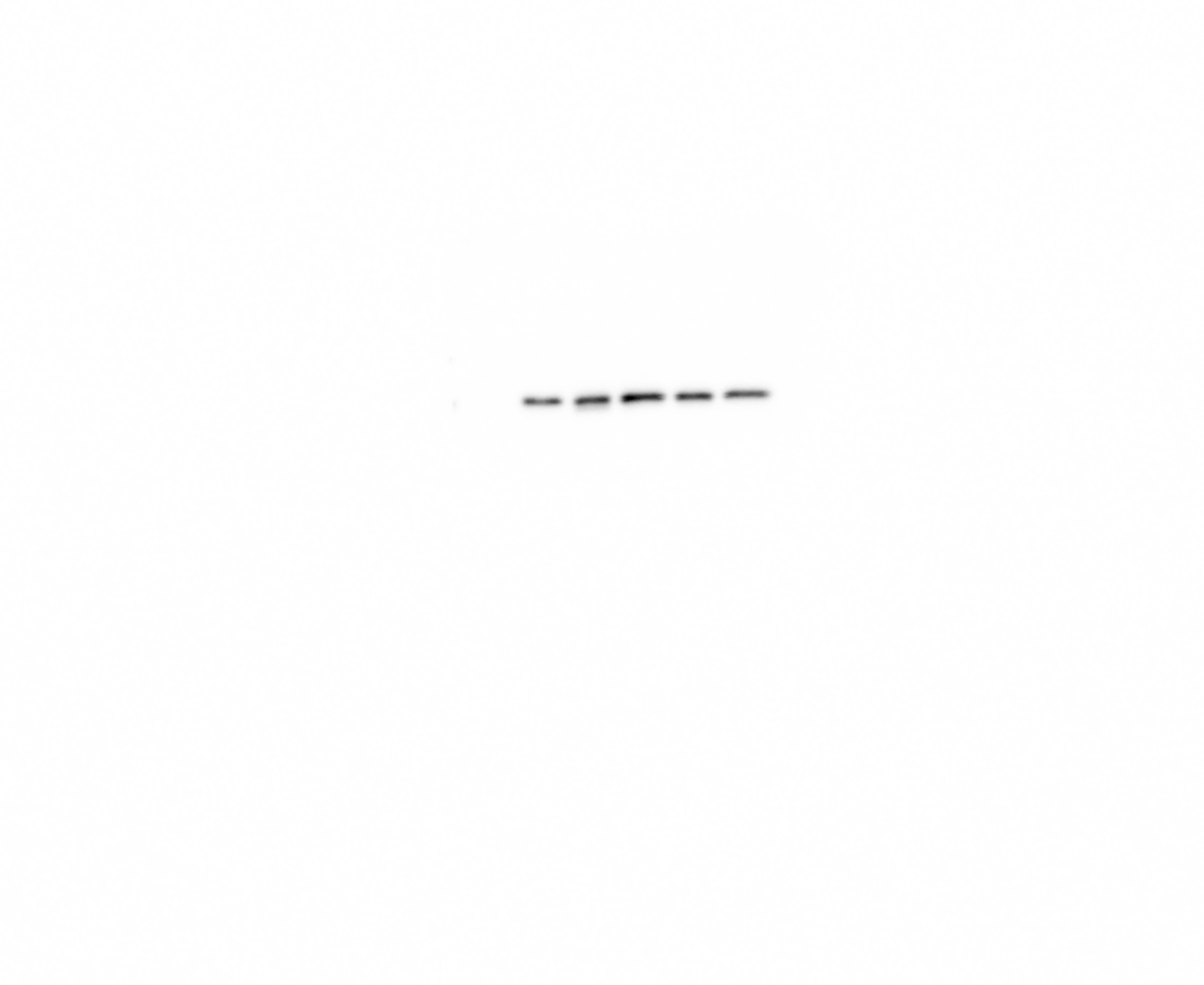

Supplement: Supplementary file 22 — EV Figure Source Data [file 44318_2024_356_MOESM22_ESM.zip › Figure EV/Fig EV4/Fig EV4A REEP5.tif]

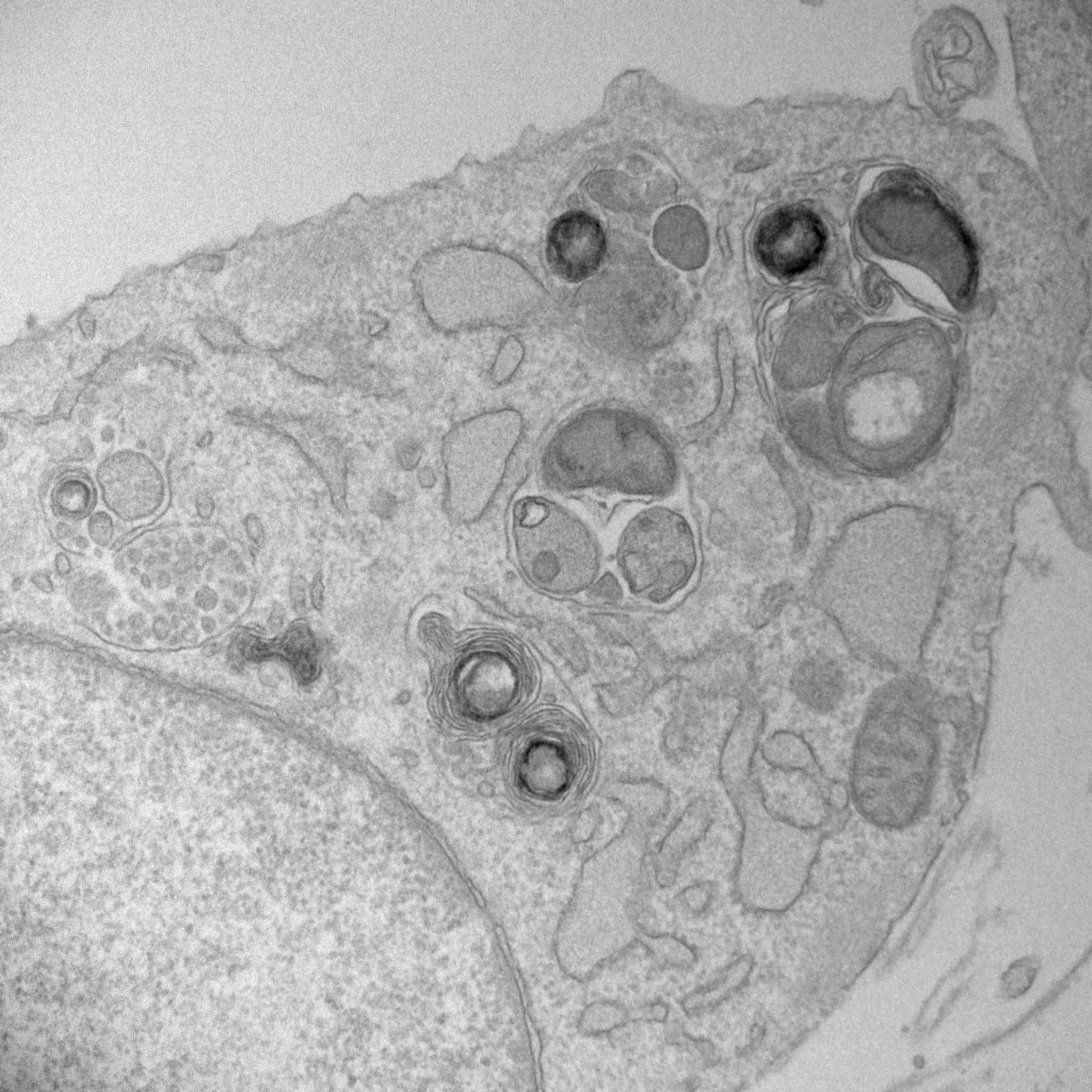

Supplement: Supplementary file 22 — EV Figure Source Data [file 44318_2024_356_MOESM22_ESM.zip › Figure EV/Fig EV4/Fig EV4D.tif]

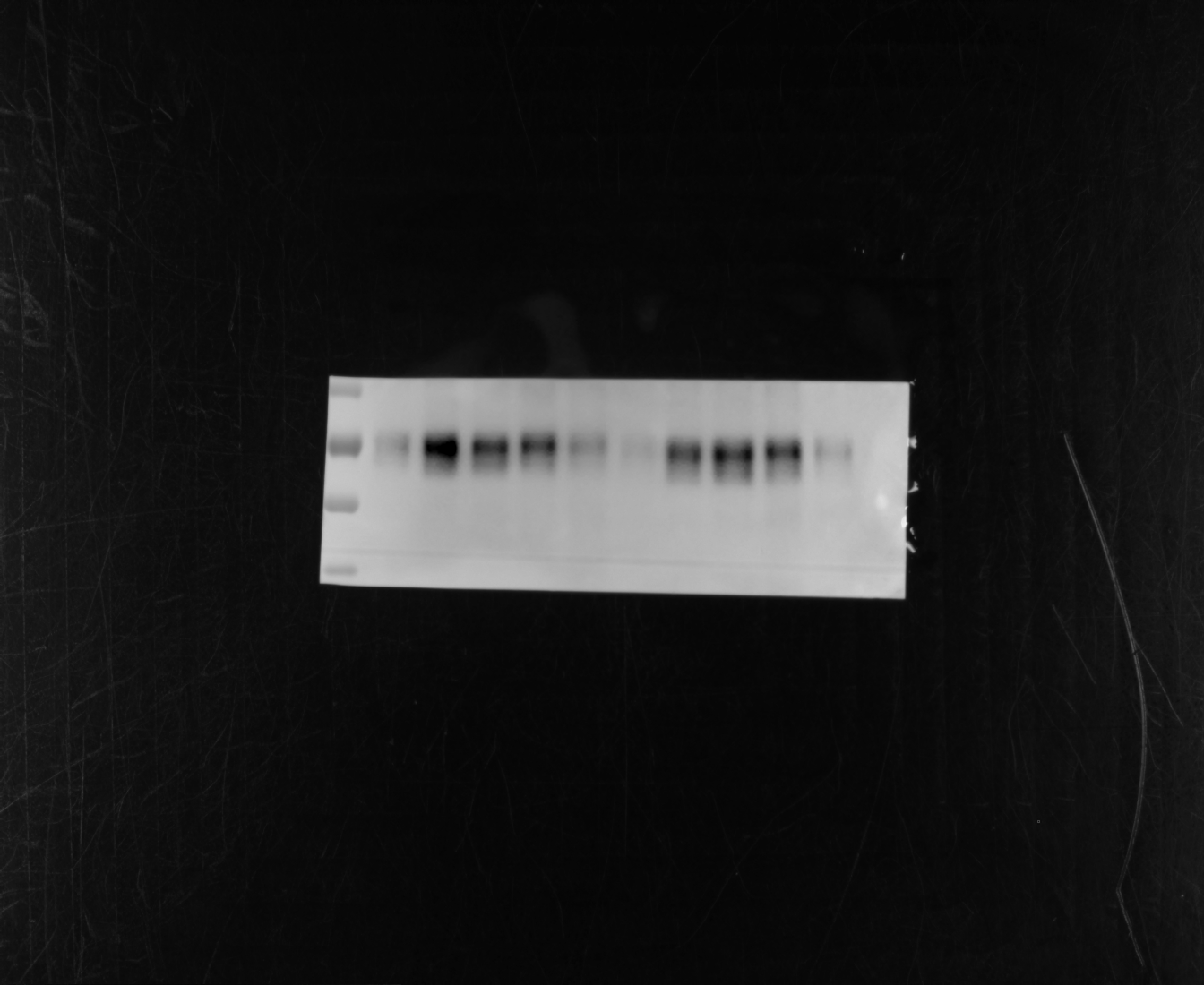

Supplement: Supplementary file 22 — EV Figure Source Data [file 44318_2024_356_MOESM22_ESM.zip › Figure EV/Fig EV4/Fig EV4B MEF2C.Tif]

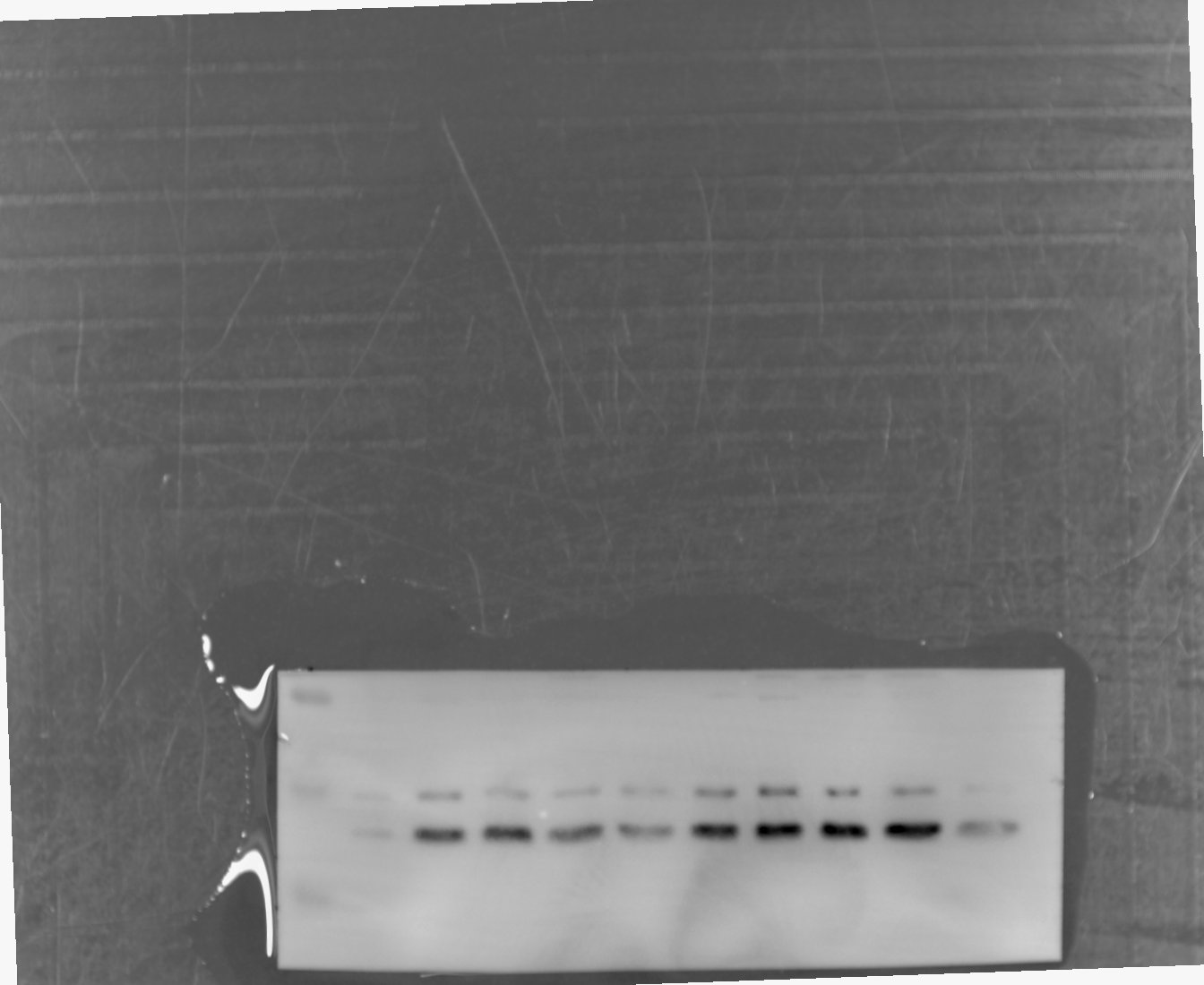

Supplement: Supplementary file 22 — EV Figure Source Data [file 44318_2024_356_MOESM22_ESM.zip › Figure EV/Fig EV4/Fig EV4B LC3B.jpg]
