# Supplementary material for: Two FAM134B isoforms differentially regulate ER dynamics during myogenesis
Source: EMBO J. 2025 Jan 6;44(4):1039–73. doi: 10.1038/s44318-024-00356-2 (PMC11832904; doi:10.1038/s44318-024-00356-2)
Supplement: Supplementary file 23 — Expanded View Figures [file 44318_2024_356_MOESM23_ESM.pdf]

Expanded View Figures

Figure EV1. Proteome profile of muscle cells during myogenesis.

(**A**) Representative WB of total cell lysate from C2C12 cells and myotubes. MF20: Myosin heavy chain; MyoG: Myogenin. Bar plots represent the densitometric quantification of WB bands. (**B–D**) Heatmap (Log_2 LFQ normalized intensity) of proteins related to the ER (**B**), lysosome (**C**) and autophagy-ubiquitin pathway (**D**) that are significantly deregulated in differentiated myotubes versus C2C12 myoblasts. (**E**, **F**) Representative WB of ER and lysosomal proteins from total cell lysate from myoblasts and myotubes. (**G**) Heatmap (Log_2 LFQ normalized intensity) of significantly regulated proteins in murine primary myoblasts and myotubes differentiated in growth medium (GM) or in differentiation medium (DM). The profile plot highlights significantly upregulated proteins. **H**) Principal components analysis (PCA) of the full proteomes from primary murine myoblasts and myotubes differentiated in growth medium (GM) or in differentiation medium (DM). (**I**) GOCC enrichment analysis from MS data with ER elements in purple and lysosomal components in green. (**J**) GOCC terms and frequencies of the upregulated proteins identified in murine myotubes. (**K**) Heatmap (LFQ normalized intensity) of significantly deregulated proteins in human myoblasts and myotubes differentiated in growth medium (GM) or in differentiation medium (DM). The profile plot highlights significantly upregulated proteins. (**L**) Principal components analysis (PCA) of the full proteomes from primary human myoblasts and myotubes differentiated in growth medium (GM) or in differentiation medium (DM). (**M**) GOCC enrichment analysis from MS data with ER elements in purple and lysosomal components in green. (**N**) GOCC terms and frequencies of the upregulated proteins identified in human myotubes. (**O**) The bar graph represents the quantification of the ER area in myoblasts and myotubes (Fig. 1A). All data are represented as mean ± s.d.; $^{***}P < 0.001$ (Student t test). Mass spectrometry was performed in triplicate. Western blots were performed in three independent experiments. Source data are available online for this figure.

© The Author(s)

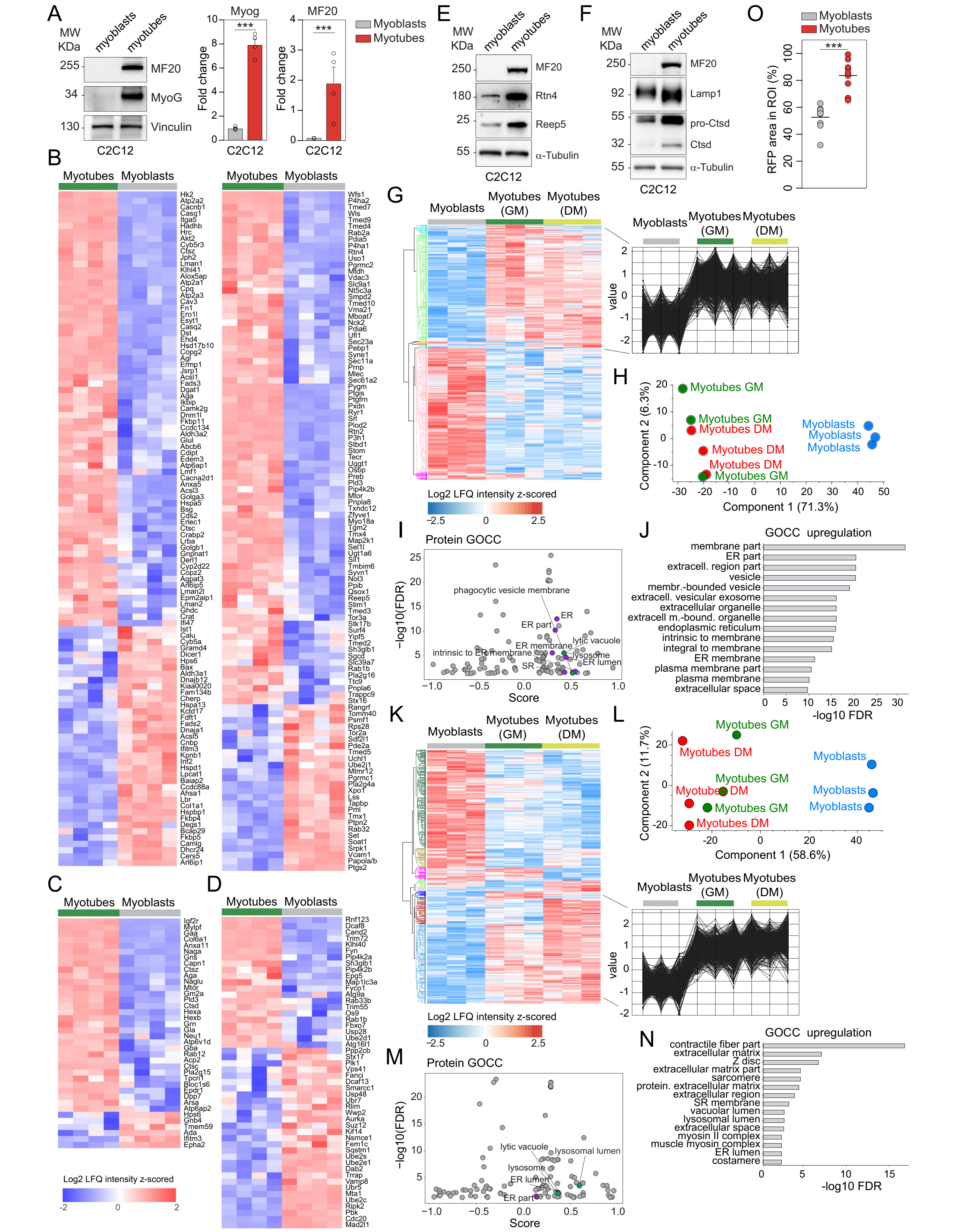
A
MW KDa
myoblasts
myotubes
255
34
130
MF20
MyoG
Vinculin
C2C12
Myog
MF20
Fold change
***
C2C12
Myoblasts
Myotubes
B
Myotubes Myoblasts
Hk2
Atp2a2
Cacnb1
Casq1
Itga5
Hadhb
Hrc
Akt2
Cyb5r3
Ctsz
Jph2
Lman1
Klhl41
Alox5ap
Atp2a1
Cpq
Atp2a3
Cav3
Fn1
Ero1l
Esyt1
Casq2
Dst
Ehd4
Hsd17b10
Copg2
Agl
Ermp1
Jsrp1
Acsl1
Fads3
Dgat1
Aga
Ikbip
Camk2g
Dnm1l
Fkbp11
Ccdc134
Aldh3a2
Glul
Abcb6
Cdipt
Edem3
Atp6ap1
Lmf1
Cacna2d1
Anxa5
Acsl3
Golga3
Hspa5
Bsg
Cds2
Erlec1
Ctsc
Crabp2
Lrba
Golgb1
Gnpnat1
Derl1
Cyp2d22
Copz2
Agpat3
Arfgap3
Epm2aip1
Lman2
Ghdc
Crat
Ifi47
Ist1
Calu
Cyb5a
Gramd4
Dicer1
Hps6
Bax
Aldh3a1
Dnajb12
Kiaa0020
Fam134b
Cherp
Hspa13
Kctd17
Fdft1
Fads2
Dnaja1
Acsl5
Cnbp
Ifitm3
Kpnb1
Inf2
Hspd1
Lpcat1
Baiap2
Ccdc88a
Ahsa1
Lbr
Col1a1
Hspbp1
Fkbp4
Degs1
Bcap29
Fkbp5
Camlg
Dhcr24
Cers5
Arl6ip1
Wfs1
P4ha2
Tmed7
Wls
Tmed9
Tmed4
Rab2a
Pdia5
P4ha1
Rtn4
Uso1
Pgrmc2
Mfdh
Vdac3
Slc9a1
Nt5c3a
Smpd2
Tmed10
Vma21
Mboat7
Nck2
Pdia6
Ufl1
Sec23a
Pebp1
Syne1
Sec11a
Prnp
Mlec
Sec61a2
Pygm
Ptgis
Ptgfrn
Pxdn
Ryr1
Srl
Plod2
Rtn2
P3h1
Stbd1
Stom
Tecr
Uggt1
Osbp
Preb
Pld3
Pip4k2b
Mtor
Pnpla8
Txndc12
Zfyve1
Myo18a
Tgm2
Tmx4
Map2k1
Sel1l
Ugt1a6
Sil1
Tmbim6
Syvn1
Nol3
Ppib
Qsox1
Reep5
Stim1
Tmed3
Tor3a
Stk17b
Surf4
Yipf5
Tmed2
Sh3glb1
Sgcd
Slc39a7
Rab1b
Pla2g16
Ttc9
Pnpla6
Trappc9
Stx16
Rangrf
Tomm40
Psmf1
Rps28
Tor2a
Sdf2l1
Pde2a
Tmed5
Uchl1
Ube2j1
Mtmr12
Pgrmc1
Pla2g4a
Xpo1
Lss
Tapbp
Pml
Tmx1
Ptpn2
Rab32
Set
Soat1
Srpk1
Vcam1
Papola/b
Ptgs2
C
Myotubes Myoblasts
Igf2r
Mylpf
Gaa
Col6a1
Anxa11
Naga
Gns
Capn1
Ctsz
Aga
Naglu
Mtor
Gm2a
Pld3
Ctsd
Hexa
Hexb
Grn
Gla
Neu1
Atp6v1d
Gba
Rab12
Acp2
Ctsc
Pla2g15
Tpcn1
Bloc1s6
Epdr1
Dpp7
Arsa
Atp6ap2
Hps6
Gnb4
Tmem59
Ada
Ifitm3
Epha2
Log2 LFQ intensity z-scored
-2 0 2
D
Myotubes Myoblasts
Rnf123
Dcaf8
Cand2
Trim72
Klhl40
Fyn
Pip4k2a
Sh3glb1
Pip4k2b
Epg5
Map1lc3a
Fyco1
Atg9a
Rab33b
Trim55
Os9
Rab1b
Fbxo7
Usp28
Ube2d1
Atg16l1
Ppp2cb
Stx17
Plk1
Vps41
Fanci
Dcaf13
Smarcc1
Usp48
Ubr7
Rlim
Wwp2
Aurka
Suz12
Kif14
Nsmce1
Fem1c
Sqstm1
Ube2s
Ube2e1
Dab2
Trrap
Ubr5
Vamp8
Mta1
Ube2c
Ripk2
Pbk
Cdc20
Mad2l1
E
MW KDa
myoblasts
myotubes
250
180
25
55
MF20
Rtn4
Reep5
α-Tubulin
C2C12
F
MW KDa
myoblasts
myotubes
250
92
55
32
55
MF20
Lamp1
pro-Ctsd
Ctsd
α-Tubulin
C2C12
O
Myoblasts
Myotubes
RFP area in ROI (%)
***
G
Myoblasts
Myotubes (GM)
Myotubes (DM)
Log2 LFQ intensity z-scored
-2.5 0 2.5
value
H
Myotubes GM
Myotubes GM
Myotubes DM
Myotubes DM
Myotubes DM
Myotubes GM
Myoblasts
Myoblasts
Myoblasts
Component 2 (6.3%)
Component 1 (71.3%)
I
Protein GOCC
phagocytic vesicle membrane
ER
ER part
ER membrane
lytic vacuole
intrinsic to ER membrane
lysosome
SR
ER lumen
-log10(FDR)
Score
J
GOCC upregulation
membrane part
ER part
extracell. region part
vesicle
membr.-bounded vesicle
extracell. vesicular exosome
extracellular organelle
extracell m.-bound. organelle
endoplasmic reticulum
intrinsic to membrane
integral to membrane
ER membrane
plasma membrane part
plasma membrane
extracellular space
-log10 FDR
K
Myoblasts
Myotubes (GM)
Myotubes (DM)
Log2 LFQ intensity z-scored
-2.5 0 2.5
value
L
Myotubes GM
Myotubes DM
Myoblasts
Myotubes GM
Myotubes DM
Myoblasts
Myotubes GM
Myoblasts
Myotubes DM
Component 2 (11.7%)
Component 1 (58.6%)
M
Protein GOCC
lytic vacuole
lysosomal lumen
lysosome
ER lumen
ER part
-log10(FDR)
Score
N
GOCC upregulation
contractile fiber part
extracellular matrix
Z disc
extracellular matrix part
sarcomere
protein. extracellular matrix
extracellular region
SR membrane
vacuolar lumen
lysosomal lumen
extracellular space
myosin II complex
muscle myosin complex
ER lumen
costamere
-log10 FDR

 © The Author(s)

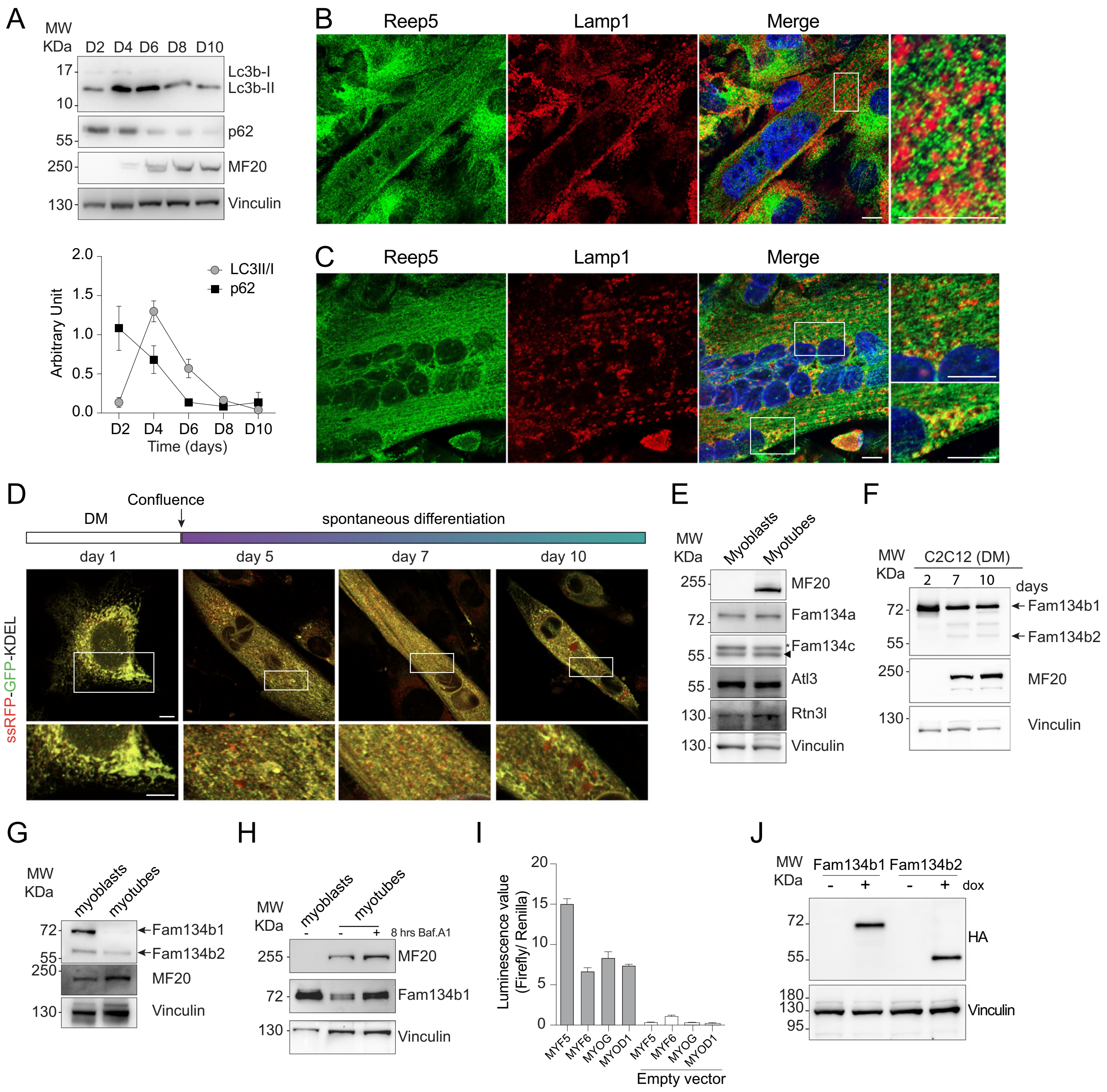

Figure EV2. Fam134b isoforms are differently modulated during myogenesis.

(**A**) WB analysis showing Lc3b, p62, and MF20 protein levels at various differentiation time points during C2C12 differentiation. Graphs represent the densitometric analysis of WB bands. (**B**) Confocal images of differentiated C2C12 cells stably expressing TMEM192-3xHA stained for HA and the ER protein REEP5. Scale bar: 10 µm. (**C**) Confocal images of differentiated human myoblasts, stained for the ER protein REEP5 and the lysosomal protein LAMP1. Scale bar: 10 µm. (**D**) Confocal images of C2C12 cells, stably overexpressing ssRFP-GFP-KDEL during their differentiation. Scale bar: 10 µm. Inset Scale bar: 5 µm. DM: differentiation medium (2% horse serum) (**E**). (**E**) WB analysis of the endogenous ER-phagy receptors in C2C12 myoblasts and myotubes. (**F**, **G**) WB analysis of endogenous FAM134B1 and FAM134B2 in C2C12 cells (**F**) and human myoblasts (**G**) differentiated into myotubes using differentiation medium (DM). (**H**) WB analysis of endogenous Fam134b1 in C2C12 myoblasts and myotubes, with and without Bafilomycin A1 (Baf.A1) treatment. MF20 was used as a muscle differentiation marker. (**I**) Graphical quantification of luciferase assays demonstrating the activity of the indicated transcription factors co-transfected, in HEK 293T), with a plasmid containing the firefly luciferase gene downstream of the Fam134b1 promoter. (**J**) WB representing the protein levels of HA-Fam134b1 and HA-Fam134b2 in C2C12 stable cell lines. Imaging, western blots and luciferase assay were performed in three independent experiments. Source data are available online for this figure.

© The Author(s)

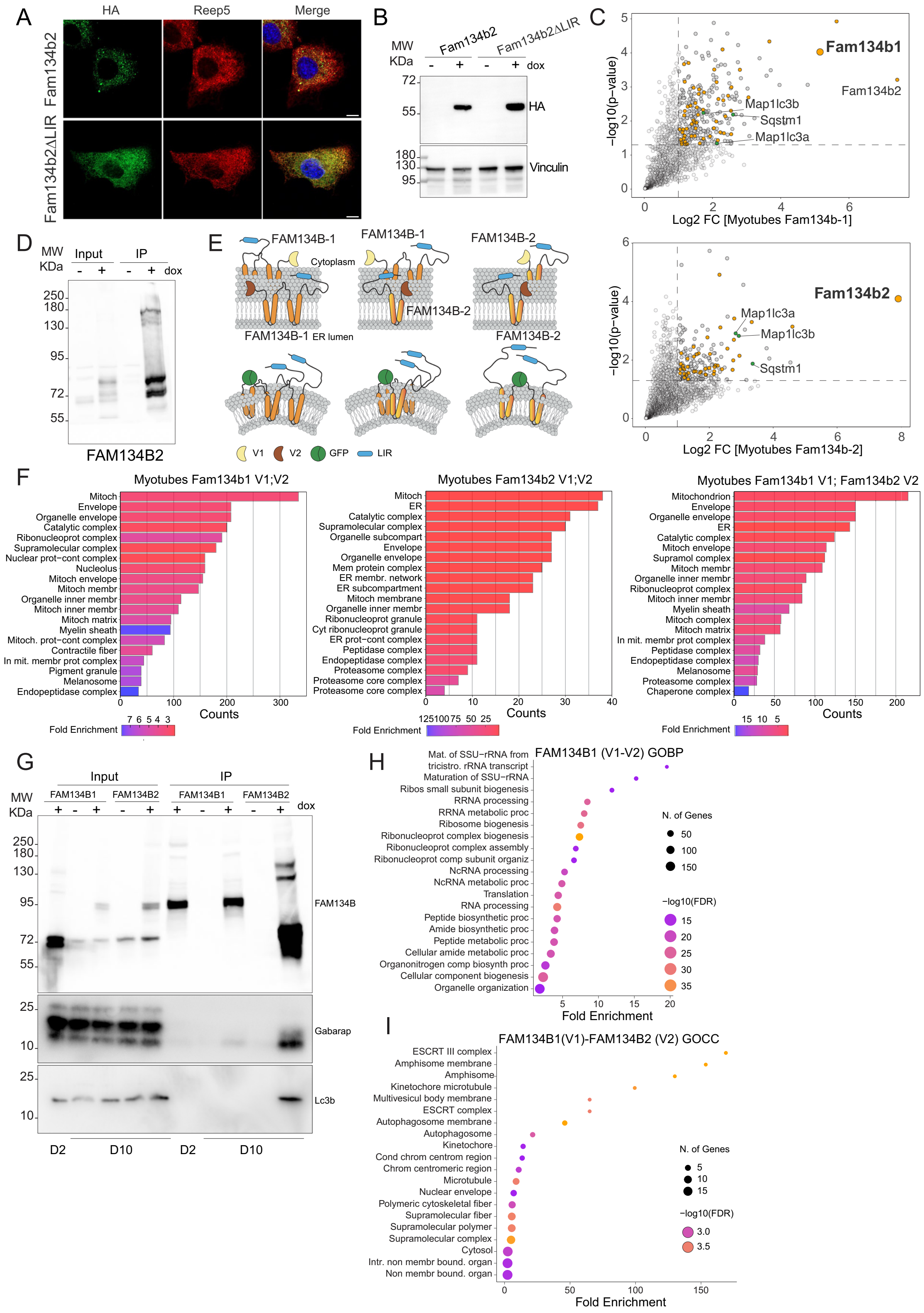
A
HA
Reep5
Merge
Fam134b2
Fam134b2ΔLIR
B
MW
KDa
Fam134b2
Fam134b2ΔLIR
- + - + dox
72
55
HA
180
130
95
Vinculin
C
Fam134b1
Fam134b2
Map1lc3b
Sqstm1
Map1lc3a
−log10(p−value)
Log2 FC [Myotubes Fam134b-1]
Fam134b2
Map1lc3a
Map1lc3b
Sqstm1
Log2 FC [Myotubes Fam134b-2]
D
MW
KDa
Input
IP
- + - + dox
250
180
130
95
72
55
FAM134B2
E
FAM134B-1
Cytoplasm
FAM134B-1 ER lumen
FAM134B-1
FAM134B-2
FAM134B-2
FAM134B-2
V1
V2
GFP
LIR
F
Myotubes Fam134b1 V1;V2
Mitoch
Envelope
Organelle envelope
Catalytic complex
Ribonucleoprot complex
Supramolecular complex
Nuclear prot−cont complex
Nucleolus
Mitoch envelope
Mitoch membr
Organelle inner membr
Mitoch inner membr
Mitoch matrix
Myelin sheath
Mitoch. prot−cont complex
Contractile fiber
In mit. membr prot complex
Pigment granule
Melanosome
Endopeptidase complex
Counts
Fold Enrichment
Myotubes Fam134b2 V1;V2
Mitoch
ER
Catalytic complex
Supramolecular complex
Organelle subcompart
Envelope
Organelle envelope
Mem protein complex
ER membr. network
ER subcompartment
Mitoch membrane
Organelle inner membr
Ribonucleoprot granule
Cyt ribonucleoprot granule
ER prot−cont complex
Peptidase complex
Endopeptidase complex
Proteasome complex
Proteasome core complex
Proteasome core complex
Counts
Fold Enrichment
Myotubes Fam134b1 V1; Fam134b2 V2
Mitochondrion
Envelope
Organelle envelope
ER
Catalytic complex
Mitoch envelope
Supramol complex
Mitoch membr
Organelle inner membr
Ribonucleoprot complex
Mitoch inner membr
Myelin sheath
Mitoch complex
Mitoch matrix
In mit. membr prot complex
Peptidase complex
Endopeptidase complex
Melanosome
Proteasome complex
Chaperone complex
Counts
Fold Enrichment
G
Input
IP
FAM134B1
FAM134B2
FAM134B1
FAM134B2
MW
KDa
dox
FAM134B
Gabarap
Lc3b
D2
D10
D2
D10
H
FAM134B1 (V1-V2) GOBP
Mat. of SSU−rRNA from
tricistro. rRNA transcript
Maturation of SSU−rRNA
Ribos small subunit biogenesis
RRNA processing
RRNA metabolic proc
Ribosome biogenesis
Ribonucleoprot complex biogenesis
Ribonucleoprot complex assembly
Ribonucleoprot comp subunit organiz
NcRNA processing
NcRNA metabolic proc
Translation
RNA processing
Peptide biosynthetic proc
Amide biosynthetic proc
Peptide metabolic proc
Cellular amide metabolic proc
Organonitrogen comp biosynth proc
Cellular component biogenesis
Organelle organization
N. of Genes
−log10(FDR)
Fold Enrichment
I
FAM134B1(V1)-FAM134B2 (V2) GOCC
ESCRT III complex
Amphisome membrane
Amphisome
Kinetochore microtubule
Multivesicul body membrane
ESCRT complex
Autophagosome membrane
Autophagosome
Kinetochore
Cond chrom centrom region
Chrom centromeric region
Microtubule
Nuclear envelope
Polymeric cytoskeletal fiber
Supramolecular fiber
Supramolecular polymer
Supramolecular complex
Cytosol
Intr. non membr bound. organ
Non membr bound. organ
N. of Genes
−log10(FDR)
Fold Enrichment

 © The Author(s)

Figure EV3. FAM134B2 mostly interacts with ER and autophagy proteins.

(**A**) Confocal images of C2C12 cells expressing HA-Fam134b2 and HA-Fam134b2ΔLIR, stained for the ER protein REEP5 and HA. Scale bar: 10 µm. (**B**) WB analysis of HA tag in C2C12 cells expressing HA-Fam134b2 and HA-Fam134b2ΔLIR. (**C**) Volcano plot illustrating the interactome of Fam134b1 (left) and Fam134b2 (right) in differentiated myotubes, highlighting ER proteins (orange dots) and the autophagy proteins (green dots). (**D**) WB analysis following RFP-tagged Fam134b2 pull-down in differentiated myotubes. (**E**) Schematic representation of the bimolecular complementation affinity purification system employed to pulldown FAM134B complexes. (**F**) GOCC terms and frequencies of the different FAM134B complexes interactors in myotubes. (**G**) IP of GFP tag in C2C12 cells expressing V1/V2-FAM134B1 or V1/V2-FAM134B2 and WB for Lc3b and Gabarap. (**H**) GOBP enrichment analysis from FAM134B1 homodimers interactors. (**I**) GOCC enrichment analysis from FAM134B1-FAM134B2 heterodimers interactors. Imagine, western blots and mass spectrometry were performed in three independent experiments. Source data are available online for this figure.

© The Author(s)

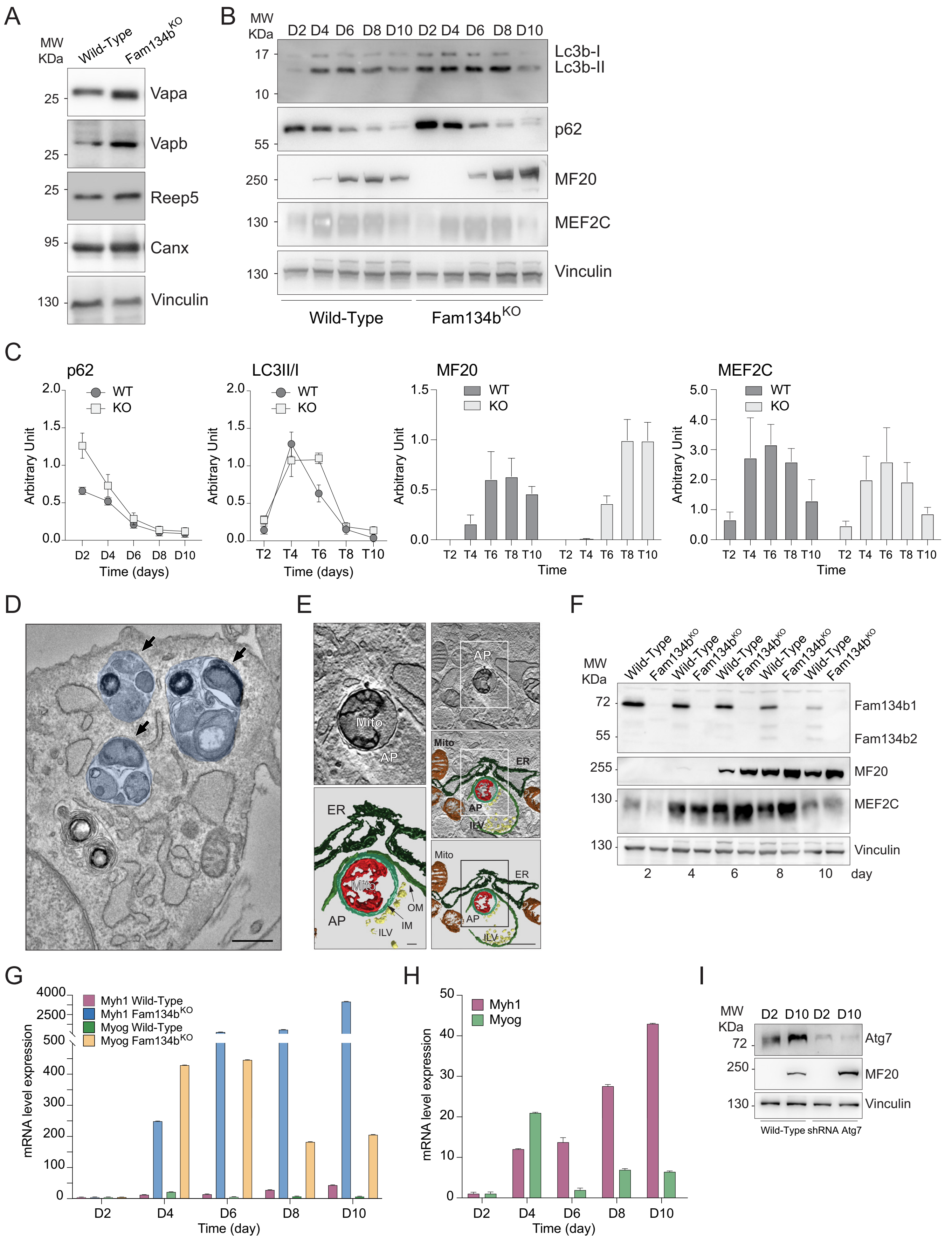
A
MW KDa
Wild-Type
Fam134b^KO
25
Vapa
25
Vapb
25
Reep5
95
Canx
130
Vinculin
B
MW KDa
D2 D4 D6 D8 D10 D2 D4 D6 D8 D10
17
10
Lc3b-I
Lc3b-II
55
p62
250
MF20
130
MEF2C
130
Vinculin
Wild-Type
Fam134b^KO
C
p62
WT
KO
Arbitrary Unit
2.0
1.5
1.0
0.5
0.0
D2 D4 D6 D8 D10
Time (days)
LC3II/I
T2 T4 T6 T8 T10
Time (days)
MF20
1.5
1.0
0.5
0.0
T2 T4 T6 T8 T10 T2 T4 T6 T8 T10
Time
MEF2C
5.0
4.0
3.0
2.0
1.0
0.0
D
E
Mito
AP
ER
OM
IM
ILV
F
MW KDa
Wild-Type
Fam134b^KO
72
55
Fam134b1
Fam134b2
255
MF20
130
MEF2C
130
Vinculin
2 4 6 8 10 day
G
4000
2500
500
400
300
200
100
0
Myh1 Wild-Type
Myh1 Fam134b^KO
Myog Wild-Type
Myog Fam134b^KO
mRNA level expression
D2 D4 D6 D8 D10
Time (day)
H
50
40
30
20
10
0
Myh1
Myog
mRNA level expression
D2 D4 D6 D8 D10
Time (day)
I
MW KDa
D2 D10 D2 D10
72
Atg7
250
MF20
130
Vinculin
Wild-Type shRNA Atg7

 © The Author(s)

Figure EV4. A proper ER-phagy flux is necessary for myogenesis.

(**A**) Representative WB analysis of endogenous ER proteins in WT and Fam134b^{KO} myotubes. (**B**) Representative WB analysis of Lc3b, p62, MF20 and MEF2C in WT and Fam134b^{KO} C2C12 cells throughout differentiation. (**C**) Densitometric analysis of WB bands in panel (**B**). (**D**) Electron microscopy images of Fam134b^{KO} myotubes, with black arrows indicating autophagosome structures. Scale bar: 400 nm. (**E**) Tomography and imagine reconstruction depicting a mitochondrion inside an autophagosome in a Fam134b^{KO} myotube. AP autophagosome, ER endoplasmic reticulum, Mito mitochondria, OM outer autophagosomal membrane (dark green), IM inner autophagosomal membranes (light green), ILV intra luminal vesicles. Red: mitochondria inside the autophagosome. Scale bar: 200 nm. (**F**) Representative WB analysis of endogenous Fam134b isoforms and the muscle differentiation markers MF20 and MEF2C in WT and Fam134b^{KO} C2C12 cells throughout differentiation. (**G**) Real time PCR analysis illustrating the expression profiles of the indicated genes during the differentiation of WT and Fam134b^{KO} C2C12 cells. (**H**) Magnified view of the bar graphs presented in panel G, focusing on WT samples. (**I**) Representative WB imagines of endogenous Atg7 and MF20 protein levels in WT and Atg7^{KD} C2C12 cells during differentiation. Data are represented as mean ± s.d. Western blots, imaging and real time pcr were performed in three independent experiments. Source data are available online for this figure.

© The Author(s)

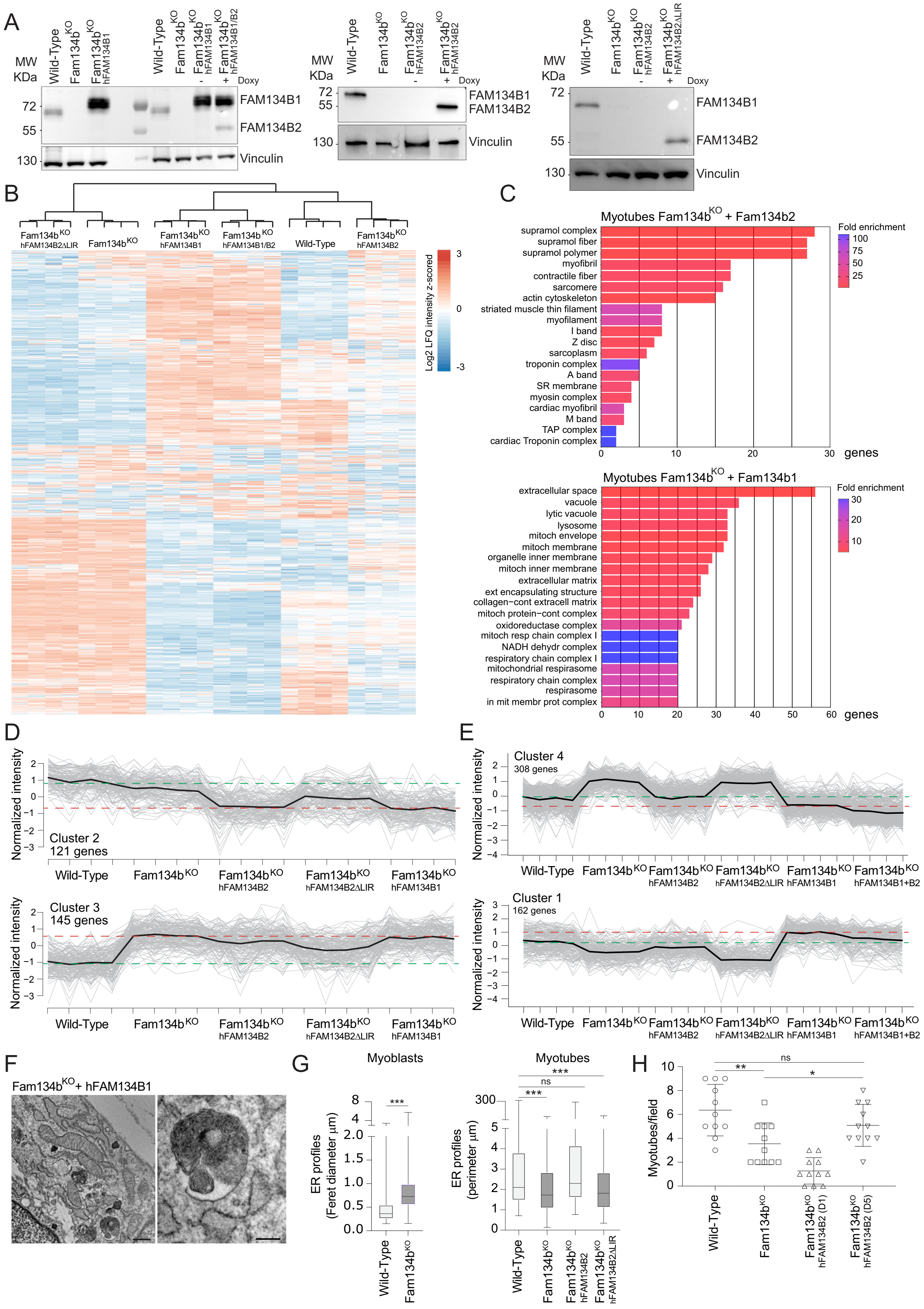
A
MW KDa
Wild-Type
Fam134b KO
Fam134b KO hFAM134B1
Fam134b KO hFAM134B2
Fam134b KO hFAM134B1/B2
Fam134b KO hFAM134B2ΔLIR
Doxy
FAM134B1
FAM134B2
Vinculin
B
Log2 LFQ intensity z-scored
C
Myotubes Fam134b KO + Fam134b2
Fold enrichment
supramol complex
supramol fiber
supramol polymer
myofibril
contractile fiber
sarcomere
actin cytoskeleton
striated muscle thin filament
myofilament
I band
Z disc
sarcoplasm
troponin complex
A band
SR membrane
myosin complex
cardiac myofibril
M band
TAP complex
cardiac Troponin complex
genes
Myotubes Fam134b KO + Fam134b1
extracellular space
vacuole
lytic vacuole
lysosome
mitoch envelope
mitoch membrane
organelle inner membrane
mitoch inner membrane
extracellular matrix
ext encapsulating structure
collagen−cont extracell matrix
mitoch protein−cont complex
oxidoreductase complex
mitoch resp chain complex I
NADH dehydr complex
respiratory chain complex I
mitochondrial respirasome
respiratory chain complex
respirasome
in mit membr prot complex
D
Normalized intensity
Cluster 2
121 genes
Cluster 3
145 genes
E
Cluster 4
308 genes
Cluster 1
162 genes
F
Fam134b KO + hFAM134B1
G
Myoblasts
Myotubes
ER profiles (Feret diameter μm)
ER profiles (perimeter μm)
H
Myotubes/field
Fam134b KO hFAM134B2 (D1)
Fam134b KO hFAM134B2 (D5)

 © The Author(s)

Figure EV5. Fam134b2 reconstitution is sufficient to rescue the proteomic abnormalities in Fam134b^{KO} myotubes.

(**A**) WB analysis of FAM134B in Fam134b^{KO} C2C12 cells reconstituted with the indicated FAM134B isoforms. (**B**) Heatmap (Log_2 LFQ normalized intensity) of the full proteomes from WT, Fam134b^{KO} and reconstituted myotubes. (**C**) GOCC terms and frequencies of the significantly deregulated proteins in the proteomic analysis of Fam134b^{KO} myotubes reconstituted with hFAM134B2 or hFAM134B1. (**D**) Profile plot showing significantly regulated proteins according to ANOVA, specifically within cluster 2 and 3 of ER associated proteins. (**E**) Profile plots of significantly altered ER proteins belonging to clusters 4 and 1, as identified by ANOVA. (**F**) Electron microscopy images of the ER in Fam134b^{KO} myotubes reconstituted with hFAM134B1. Scale bar: 500 nm. Insets highlight autophagosome structures. Scale bar: 200 nm. (**G**) Left histograms represent the Feret's diameter measures from the ER of Wild-Type and Fam134b^{KO} myoblasts. Right histograms represent the perimeter of the ER from Wild-Type, Fam134b^{KO} and reconstituted myotubes. (**H**) Quantitative analysis of myotubes number in Wild-Type and Fam134b^{KO} C2C12 after 10 days of differentiation as well as in Fam134b^{KO} C2C12 overexpressing hFAM134B1 since D1 or hFAM134B2 from D5 of differentiation. Data are represented as mean ± s.d. *$P < 0.05$; **$P < 0.01$; ***$P < 0.001$ (Mann–Witney EV5G; one-way ANOVA EV5H). Mass spectrometry was performed in quadruplicate. Western blots and imaging were performed in three independent experiments. Source data are available online for this figure.

© The Author(s)
